# Supplementary material for: Empowering consumers to PREVENT diet-related diseases through OMICS sciences (PREVENTOMICS): protocol for a parallel double-blinded randomised intervention trial to investigate biomarker-based nutrition plans for weight loss
Source: BMJ Open. 2022 Mar 29;12(3):e051285. doi: 10.1136/bmjopen-2021-051285 (PMC8966553; doi:10.1136/bmjopen-2021-051285)
Supplement: Supplementary data [file bmjopen-2021-051285supp001.pdf]

SUPPLEMENTARY ONLINE MATERIAL 1

Table 1: Biomarkers included in the carbohydrate cluster

**(1) Carbohydrates cluster**		
**Biomarker**	**Bio-fluid**	**Reference**
**Glucose**	Plasma	Cosentino et al., 2020[1]
**HOMA-IR**	-	Bloomgarden, 2003[2]<br>Govers et al., 2015[3]<br>Shashaj et al., 2016[4]
**Insulin**	Serum	Aleksandrova et al., 2018[5]<br>Bloomgarden, 2003[2]<br>Govers et al., 2015[3]
**Leptin**	Serum	Chen et al., 2014[6]<br>Finucane et al., 2019[7]<br>López-Jaramillo et al., 2014[8]
**Adiponectin**	Serum	Dastani et al., 2012[9]<br>Li et al., 2009[10]<br>Liu et al., 2016[11]<br>Wang et al., 2018[12]
**Lactate**	Serum	Berhane et al., 2015[13]<br>Choi et al., 2002[14]<br>Lovejoy et al., 1992[15]<br>Shantha et al., 2013[16]
**Glutamate**	Serum	Martin and Price, 2018[17]<br>Ottosson et al., 2018[18]
**Uric acid**	Serum	Darmawan et al., 2018[19]<br>Fabbrini et al., 2014[20]<br>Johnson et al., 2013[21]<br>van der Schaft et al., 2017[22]
**Propionylcarnitine**	Plasma	Mai et al., 2013[23]<br>Mihalik et al., 2010[24]<br>Zhang et al., 2014[25]
**BCAA (Valine, Leucine, Isoleucine)**	Serum	Chen et al., 2016[26]<br>Katagiri et al., 2018[27]<br>Lotta et al., 2016[28]<br>Newgard et al., 2009[29]<br>Okekunle et al., 2019[30]
**Phenylalanine**	Serum	Chen et al., 2019[31]<br>Suzuki et al., 2019[32]<br>Wang et al., 2011[33]
**Tyrosine**	Serum	Chen et al., 2019[31]<br>Hellmuth et al., 2016[34]<br>Wang et al., 2011[33]
**Glutamine**	Serum	Chen et al., 2019[31]<br>Guasch-Ferré et al., 2016[35]<br>Liu et al., 2019[36]<br>Rhee et al., 2018[37]

Abbreviations: HOMA-IR, homeostatic model assessment of insulin resistance; BCAA, branched chain amino acids.

Aldubayan MA, *et al. BMJ Open* 2022; 12:e051285. doi: 10.1136/bmjopen-2021-051285

Table 2: Biomarkers included in the lipid cluster

(2) Lipid cluster		
**Biomarker**	**Bio-fluid**	**Reference**
**LDL-cholesterol**	Plasma	Grundy et al., 2019[38]
**HDL-cholesterol**	Plasma	Grundy et al., 2019[38]
**Triglycerides**	Plasma	Grundy et al., 2019[38]
**Total cholesterol**	Plasma	Grundy et al., 2019[38]
**PUFAs (total)**	Serum	Koga et al., 2019[39]
**LPCs (total)**	Plasma	Law et al., 2019[40]
**Linoleic acid C18:2**	Serum	Koga et al., 2019[39]
**Oleic acid C18:1**	Serum	Griel and Kris-Etherton, 2006[41]<br>Yamagishi et al., 2013[42]
**Leptin**	Serum	Mantzoros and Flier, 2000[43]
**Adiponectin**	Serum	Abdella and Mojiminiyi, 2018[44]<br>Liu et al., 2018[45]
**SFAs**	Serum	Liu et al., 2019[46]
**3-hydroxybutyrate**	Serum	Margolis and O'Fallon, 2020[47]
**MUFAs (total)**	Serum	Griel and Kris-Etherton, 2006[41]<br>Yamagishi et al., 2013[42]
**Propionylcarnitine**	Plasma	Ottosson et al., 2018[18]
**DHA C20:3**	Serum	Koga et al., 2019[39]
**Choline**	Serum	Ottosson et al., 2018[18]

Abbreviations: LDL, low-density lipoprotein; HDL, high-density lipoprotein; PUFAs, poly unsaturated fatty acids; LPC, lysophosphatidylcholine; SFAs, saturated fatty acids; MUFAs, monounsaturated fatty acids; DHA, docosahexaenoic acid.

Table 3: Biomarkers included in the inflammation cluster

**(3) Inflammation cluster**		
**Biomarker**	**Bio-fluid**	**Reference**
**CRP**	Serum	Amsterdam, 2003[48]<br>Knight, 2015[49]<br>Liu et al., 2016[11]<br>Musunuru et al., 2008[50]<br>Wang et el., 2013[51]
**IL-6**	Plasma	Liu et al., 2016[11]<br>Wang et al., 2013[51]
**N-acetylglycoproteins**	Serum	Gruppen et al., 2019[52]<br>Ritchie et al., 2015[53]
**TNFα**	Plasma	Liu et al., 2016[11]
**MCP-1**	Plasma	Georgakis et al., 2019[54]
**IL-10**	Plasma	Charles et al., 2011[55]<br>Leon-Cabrera et al., 2015[56]<br>Meng et al., 2019[57]
**sICAM1**	Plasma	El Amine et al., 2010[58]<br>Luc et al., 2003[59]<br>Straczkowski et al., 2002[60]
**LBP**	Plasma	Moreno-Navarrete et al., 2012[61]
**sCD14**	Plasma	de Courten et al., 2016[62]
**LPCs (total)**	Plasma	Iwase et al., 2008[63]
**Linoleic acid C18:2**	Serum	Steffen et al., 2012[64]<br>Yli-Jama et al., 2002[65]
**DHA C20:3**	Serum	Steffen et al., 2012[64]<br>Yli-Jama et al., 2002[65]

Abbreviations: CRP, C-reactive protein; IL-6, interleukin-6; IL-10, interleukin-10; TNFα, tumor necrosis factor alpha; MCP-1, monocyte chemoattractant protein-1; sICAM-1, soluble intercellular adhesion molecule-1; LBP, lipopolysaccharide binding protein; sCD14, soluble CD14; LPC, lysophosphatidylcholines; DHA: docosahexaenoic acid.

Aldubayan MA, *et al. BMJ Open* 2022; 12:e051285. doi: 10.1136/bmjopen-2021-051285

Table 4: Biomarkers included in the oxidative stress cluster

**(4) Oxidative stress cluster**		
**Biomarker**	**Bio-fluid**	**Reference**
**8-iso-PGF2α**	Urine	Davies and Roberts, 2011[66]<br>Kim et al., 2012[67]<br>Milne et al., 2015[68]<br>van't Erve, 2018[69]
**8-OHdG**	Urine	Di Minno et al., 2016[70]<br>Kroese and Scheffe, 2014[71]
**LDLox**	Plasma	Barbosa et al., 2011[72]<br>Gao et al., 2017[73]
**Uric acid**	Serum	Darmawan et al., 2018[19]<br>Fabbrini et al., 2014[20]<br>van der Schaft et al., 2017[22]
**Allantoin**	Urine	Il'yasova et al., 2012[74]
**Betaine**	Urine	Svingen et al., 2016[75]<br>Walford et al., 2016[76]
**Pseudouridine**	Urine	Topp et al., 2008[77]
**Dimethylglycine**	Urine	Svingen et al., 2016[75]
**Glycine**	Serum	Sekhar et al., 2011[78]
**Methionine**	Serum	Grizales et al., 2018[79]

Abbreviations: 8-iso-PGF2α, 8-iso-prostaglandin F2 alpha; 8-OHdG, 8-hydroxydeoxyguanosine; LDLox, oxidized low density lipoprotein.

Table 5: Biomarkers included in the microbiota cluster

**(5) Microbiota cluster**		
**Biomarker**	**Bio-fluid**	**Reference**
**TMAO**	Serum	Bain et al., 2006[80]<br>Chen et al., 2016[81]<br>Ge et al., 2020[82]<br>Heianza et al., 2017[83]<br>Schiattarella et al., 2017[84]<br>Yang et el., 2019[85]<br>Yao et al., 2020[86]
**TMA**	Urine	Aragonès et al., 2019[87]<br>Bouatra et al., 2013[88]<br>Chen et al., 2016[81]<br>Palau-Rodriguez et al., 2015[89]<br>Yang et el., 2019[85]
**DMA**	Urine	Bouatra et al., 2013[88]<br>Palau-Rodriguez et al., 2015[89]
**Betaine**	Urine	Chen et al., 2016[81]<br>Palau-Rodriguez et al., 2015[89]<br>Yang et el., 2019[85]
**Choline**	Serum	Aragonès et al., 2019[87]<br>Chen et al., 2016[81]<br>Palau-Rodriguez et al., 2015[89]<br>Yang et el., 2019[85]
**Dimethylglycine**	Urine	Palau-Rodriguez et al., 2015[89]
**Succinate**	Plasma	Psychogios et al., 2011[90]<br>Serena et al., 2018[91]
**Lactate**	Serum	Psychogios et al., 2011[90]<br>Vojinovic et al., 2019[92]
**LBP**	Plasma	Awoyemi et al., 2018[93]<br>Barengolts et al., 2019[94]<br>Bouatra et al., 2013[88]<br>Citronberg et al., 2018[95]
**Acetate**	Urine	Liu et al., 2014[96]

Abbreviations: TMAO, trimethylamine N-oxide; TMA, trimethylamine; DMA, dimethylamine; LBP, lipopolysaccharide binding protein.

References:

1. Cosentino F, Grant PJ, Aboyans V, et al. 2019 ESC Guidelines on diabetes, pre-diabetes, and cardiovascular diseases developed in collaboration with the EASD. *Eur Heart J* 2020;41(2):255-323.
2. Bloomgarden ZT. American Association of Clinical Endocrinologists (AACE) consensus conference on the insulin resistance syndrome: 25-26 August 2002, Washington, DC. *Diabetes Care* 2003;26(4):1297-303.
3. Govers E, Slof E, Verkoelen H, et al. Guideline for the Management of Insulin Resistance. *Int J Endocrinol Metab Disord* 2015;1(4).
4. Shashaj B, Luciano R, Contoli B, et al. Reference ranges of HOMA-IR in normal-weight and obese young Caucasians. *Acta Diabetol* 2016;53(2):251-60.
5. Aleksandrova K, Mozaffarian D, Pischon T. Addressing the Perfect Storm: Biomarkers in Obesity and Pathophysiology of Cardiometabolic Risk. *Clin Chem* 2018;64(1):142-53.
6. Chen GC, Qin LQ, Ye JK. Leptin levels and risk of type 2 diabetes: gender-specific meta-analysis. *Obes Rev* 2014;15(2):134-42.
7. Finucane FM, Luan J, Wareham NJ, et al. Correlation of the leptin:adiponectin ratio with measures of insulin resistance in non-diabetic individuals. *Diabetologia* 2009;52(11):2345-49.
8. López-Jaramillo P, Gómez-Arbeláez D, López-López J, et al. The role of leptin/adiponectin ratio in metabolic syndrome and diabetes. *Horm Mol Biol Clin Investig* 2014;18(1):37-45.
9. Dastani Z, Hivert MF, Timpson N, et al. Novel loci for adiponectin levels and their influence on type 2 diabetes and metabolic traits: a multi-ethnic meta-analysis of 45,891 individuals. *PLoS Genet* 2012;8(3):e1002607.
10. Li S, Shin HJ, Ding EL, et al. Adiponectin levels and risk of type 2 diabetes: a systematic review and meta-analysis. *Jama* 2009;302(2):179-88.
11. Liu C, Feng X, Li Q, et al. Adiponectin, TNF-α and inflammatory cytokines and risk of type 2 diabetes: A systematic review and meta-analysis. *Cytokine* 2016;86:100-09.
12. Wang Y, Meng RW, Kunutsor SK, et al. Plasma adiponectin levels and type 2 diabetes risk: a nested case-control study in a Chinese population and an updated meta-analysis. *Sci Rep* 2018;8(1):406.
13. Berhane F, Fite A, Daboul N, et al. Plasma Lactate Levels Increase during Hyperinsulinemic Euglycemic Clamp and Oral Glucose Tolerance Test. *J Diabetes Res* 2015;2015:102054.
14. Choi CS, Kim YB, Lee FN, et al. Lactate induces insulin resistance in skeletal muscle by suppressing glycolysis and impairing insulin signaling. *Am J Physiol Endocrinol Metab* 2002;283(2):E233-40.
15. Lovejoy J, Newby FD, Gebhart SSP, et al. Insulin resistance in obesity is associated with elevated basal lactate levels and diminished lactate appearance following intravenous glucose and insulin. *Metabolism* 1992;41(1):22-27.
16. Shantha GP, Wasserman B, Astor BC, et al. Association of blood lactate with carotid atherosclerosis: the Atherosclerosis Risk in Communities (ARIC) Carotid MRI Study. *Atherosclerosis* 2013;228(1):249-55.
17. Martin AJ, Price CI. A Systematic Review and Meta-Analysis of Molecular Biomarkers Associated with Early Neurological Deterioration Following Acute Stroke. *Cerebrovasc Dis* 2018;46(5-6):230-41.
18. Ottosson F, Brunkwall L, Ericson U, et al. Connection Between BMI-Related Plasma Metabolite Profile and Gut Microbiota. *J Clin Endocrinol Metab* 2018;103(4):1491-501.
19. Darmawan G, Hamijoyo L, Hasan I. Association between Serum Uric Acid and Non-Alcoholic Fatty Liver Disease: A Meta-Analysis. *Acta Med Indones* 2017;49(2):136-47.
20. Fabbrini E, Serafini M, Colic Baric I, et al. Effect of plasma uric acid on antioxidant capacity, oxidative stress, and insulin sensitivity in obese subjects. *Diabetes* 2014;63(3):976-81.
21. Johnson RJ, Nakagawa T, Sanchez-Lozada LG, et al. Sugar, uric acid, and the etiology of diabetes and obesity. *Diabetes* 2013;62(10):3307-15.
22. van der Schaft N, Brahimaj A, Wen KX, et al. The association between serum uric acid and the incidence of prediabetes and type 2 diabetes mellitus: The Rotterdam Study. *PLoS One* 2017;12(6):e0179482.
23. Mai M, Tönjes A, Kovacs P, et al. Serum levels of acylcarnitines are altered in prediabetic conditions. *PLoS One* 2013;8(12):e82459.
24. Mihalik SJ, Goodpaster BH, Kelley DE, et al. Increased levels of plasma acylcarnitines in obesity and type 2 diabetes and identification of a marker of glucolipotoxicity. *Obesity (Silver Spring)* 2010;18(9):1695-700.
25. Zhang X, Zhang C, Chen L, et al. Human serum acylcarnitine profiles in different glucose tolerance states. *Diabetes Res Clin Pract* 2014;104(3):376-82.
26. Chen T, Ni Y, Ma X, et al. Branched-chain and aromatic amino acid profiles and diabetes risk in Chinese populations. *Sci Rep* 2016;6:20594.
27. Katagiri R, Goto A, Budhathoki S, et al. Association between plasma concentrations of branched-chain amino acids and adipokines in Japanese adults without diabetes. *Sci Rep* 2018;8(1):1043.

28. Lotta LA, Scott RA, Sharp SJ, et al. Genetic Predisposition to an Impaired Metabolism of the Branched-Chain Amino Acids and Risk of Type 2 Diabetes: A Mendelian Randomisation Analysis. *PLoS Med* 2016;13(11):e1002179.
29. Newgard CB, An J, Bain JR, et al. A branched-chain amino acid-related metabolic signature that differentiates obese and lean humans and contributes to insulin resistance. *Cell Metab* 2009;9(4):311-26.
30. Okekunle AP, Zhang M, Wang Z, et al. Dietary branched-chain amino acids intake exhibited a different relationship with type 2 diabetes and obesity risk: a meta-analysis. *Acta Diabetol* 2019;56(2):187-95.
31. Chen S, Akter S, Kuwahara K, et al. Serum amino acid profiles and risk of type 2 diabetes among Japanese adults in the Hitachi Health Study. *Sci Rep* 2019;9(1):7010.
32. Suzuki Y, Kido J, Matsumoto S, et al. Associations among amino acid, lipid, and glucose metabolic profiles in childhood obesity. *BMC Pediatr* 2019;19(1):273.
33. Wang TJ, Larson MG, Vasan RS, et al. Metabolite profiles and the risk of developing diabetes. *Nat Med* 2011;17(4):448-53.
34. Hellmuth C, Kirchberg FF, Lass N, et al. Tyrosine Is Associated with Insulin Resistance in Longitudinal Metabolomic Profiling of Obese Children. *J Diabetes Res* 2016;2016:2108909.
35. Guasch-Ferré M, Hruby A, Toledo E, et al. Metabolomics in Prediabetes and Diabetes: A Systematic Review and Meta-analysis. *Diabetes Care* 2016;39(5):833-46.
36. Liu X, Zheng Y, Guasch-Ferré M, et al. High plasma glutamate and low glutamine-to-glutamate ratio are associated with type 2 diabetes: Case-cohort study within the PREDIMED trial. *Nutr Metab Cardiovasc Dis* 2019;29(10):1040-49.
37. Rhee SY, Jung ES, Park HM, et al. Plasma glutamine and glutamic acid are potential biomarkers for predicting diabetic retinopathy. *Metabolomics* 2018;14(7):89.
38. Grundy SM, Stone NJ, Bailey AL, et al. 2018 AHA/ACC/AACVPR/AAPA/ABC/ACPM/ADA/AGS/APhA/ASPC/NLA/PCNA Guideline on the Management of Blood Cholesterol: A Report of the American College of Cardiology/American Heart Association Task Force on Clinical Practice Guidelines. *J Am Coll Cardiol* 2019;73(24):e285-e350.
39. Koga N, Ogura J, Yoshida F, et al. Altered polyunsaturated fatty acid levels in relation to proinflammatory cytokines, fatty acid desaturase genotype, and diet in bipolar disorder. *Transl Psychiatry* 2019;9(1):208.
40. Law SH, Chan ML, Marathe GK, et al. An Updated Review of Lysophosphatidylcholine Metabolism in Human Diseases. *Int J Mol Sci* 2019;20(5).
41. Griel AE, Kris-Etherton PM. Tree nuts and the lipid profile: a review of clinical studies. *Br J Nutr* 2006;96 Suppl 2:S68-78.
42. Yamagishi K, Folsom AR, Steffen LM. Plasma fatty acid composition and incident ischemic stroke in middle-aged adults: the Atherosclerosis Risk in Communities (ARIC) Study. *Cerebrovasc Dis* 2013;36(1):38-46.
43. Mantzoros CS, Flier JS. Editorial: leptin as a therapeutic agent--trials and tribulations. *J Clin Endocrinol Metab* 2000;85(11):4000-2.
44. Abdella NA, Mojiminiyi OA. Clinical Applications of Adiponectin Measurements in Type 2 Diabetes Mellitus: Screening, Diagnosis, and Marker of Diabetes Control. *Dis Markers* 2018;2018:5187940.
45. Liu Z, Liang S, Que S, et al. Meta-Analysis of Adiponectin as a Biomarker for the Detection of Metabolic Syndrome. *Front Physiol* 2018;9:1238.
46. Liu Q, Matthan NR, Manson JE, et al. Plasma Phospholipid Fatty Acids and Coronary Heart Disease Risk: A Matched Case-Control Study within the Women's Health Initiative Observational Study. *Nutrients* 2019;11(7).
47. Margolis LM, O'Fallon KS. Utility of Ketone Supplementation to Enhance Physical Performance: A Systematic Review. *Adv Nutr* 2020;11(2):412-19.
48. Amsterdam EA. C-reactive protein: a guideline for its application. *Prev Cardiol* 2003;6(2):70.
49. Knight ML. The Application of High-Sensitivity C-Reactive Protein in Clinical Practice: A 2015 Update. *US Pharm* 2015;40(2):50-53.
50. Musunuru K, Kral BG, Blumenthal RS, et al. The use of high-sensitivity assays for C-reactive protein in clinical practice. *Nat Clin Pract Cardiovasc Med* 2008;5(10):621-35.
51. Wang X, Bao W, Liu J, et al. Inflammatory markers and risk of type 2 diabetes: a systematic review and meta-analysis. *Diabetes Care* 2013;36(1):166-75.
52. Gruppen EG, Kunutsor SK, Kieneker LM, et al. GlycA, a novel pro-inflammatory glycoprotein biomarker is associated with mortality: results from the PREVEND study and meta-analysis. *J Intern Med* 2019;286(5):596-609.
53. Ritchie SC, Würtz P, Nath AP, et al. The Biomarker GlycA Is Associated with Chronic Inflammation and Predicts Long-Term Risk of Severe Infection. *Cell Syst* 2015;1(4):293-301.

54. Georgakis MK, Malik R, Björkbacka H, et al. Circulating Monocyte Chemoattractant Protein-1 and Risk of Stroke: Meta-Analysis of Population-Based Studies Involving 17 180 Individuals. *Circ Res* 2019;125(8):773-82.
55. Charles BA, Doumatey A, Huang H, et al. The roles of IL-6, IL-10, and IL-1RA in obesity and insulin resistance in African-Americans. *J Clin Endocrinol Metab* 2011;96(12):E2018-22.
56. Leon-Cabrera S, Arana-Lechuga Y, Esqueda-León E, et al. Reduced systemic levels of IL-10 are associated with the severity of obstructive sleep apnea and insulin resistance in morbidly obese humans. *Mediators Inflamm* 2015;2015:493409.
57. Meng D, Liang L, Guo X. Serum interleukin-10 level in patients with inflammatory bowel disease: A meta-analysis. *Eur J Inflamm* 2019;17:1-7.
58. El Amine M, Sohawon S, Lagneau L, et al. Plasma levels of ICAM-1 and circulating endothelial cells are elevated in unstable types 1 and 2 diabetes. *Endocr Regul* 2010;44(1):17-24.
59. Luc G, Arveiler D, Evans A, et al. Circulating soluble adhesion molecules ICAM-1 and VCAM-1 and incident coronary heart disease: the PRIME Study. *Atherosclerosis* 2003;170(1):169-76.
60. Straczkowski M, Lewczuk P, Dzienis-Straczkowska S, et al. Elevated soluble intercellular adhesion molecule-1 levels in obesity: relationship to insulin resistance and tumor necrosis factor-alpha system activity. *Metabolism* 2002;51(1):75-8.
61. Moreno-Navarrete JM, Ortega F, Serino M, et al. Circulating lipopolysaccharide-binding protein (LBP) as a marker of obesity-related insulin resistance. *Int J Obes (Lond)* 2012;36(11):1442-9.
62. de Courten B, Moreno-Navarrete JM, Lyons J, et al. Contrasting association of circulating sCD14 with insulin sensitivity in non-obese and morbidly obese subjects. *Mol Nutr Food Res* 2016;60(1):103-9.
63. Iwase M, Sonoki K, Sasaki N, et al. Lysophosphatidylcholine contents in plasma LDL in patients with type 2 diabetes mellitus: relation with lipoprotein-associated phospholipase A2 and effects of simvastatin treatment. *Atherosclerosis* 2008;196(2):931-6.
64. Steffen BT, Steffen LM, Tracy R, et al. Obesity modifies the association between plasma phospholipid polyunsaturated fatty acids and markers of inflammation: the Multi-Ethnic Study of Atherosclerosis. *Int J Obes (Lond)* 2012;36(6):797-804.
65. Yli-Jama P, Seljeflot I, Meyer HE, et al. Serum non-esterified very long-chain PUFA are associated with markers of endothelial dysfunction. *Atherosclerosis* 2002;164(2):275-81.
66. Davies SS, Roberts LJ, 2nd. F2-isoprostanes as an indicator and risk factor for coronary heart disease. *Free Radic Biol Med* 2011;50(5):559-66.
67. Kim JY, Lee JW, Youn YJ, et al. Urinary levels of 8-iso-prostaglandin f2α and 8-hydroxydeoxyguanine as markers of oxidative stress in patients with coronary artery disease. *Korean Circ J* 2012;42(9):614-7.
68. Milne GL, Dai Q, Roberts LJ, 2nd. The isoprostanes--25 years later. *Biochim Biophys Acta* 2015;1851(4):433-45.
69. van 't Erve TJ. Strategies to decrease oxidative stress biomarker levels in human medical conditions: A meta-analysis on 8-iso-prostaglandin F(2α). *Redox Biol* 2018;17:284-96.
70. Di Minno A, Turnu L, Porro B, et al. 8-Hydroxy-2-Deoxyguanosine Levels and Cardiovascular Disease: A Systematic Review and Meta-Analysis of the Literature. *Antioxid Redox Signal* 2016;24(10):548-55.
71. Kroese LJ, Scheffer PG. 8-hydroxy-2'-deoxyguanosine and cardiovascular disease: a systematic review. *Curr Atheroscler Rep* 2014;16(11):452.
72. Barbosa KB, Volp AC, Hermsdorff HH, et al. Relationship of oxidized low density lipoprotein with lipid profile and oxidative stress markers in healthy young adults: a translational study. *Lipids Health Dis* 2011;10:61.
73. Gao S, Zhao D, Wang M, et al. Association Between Circulating Oxidized LDL and Atherosclerotic Cardiovascular Disease: A Meta-analysis of Observational Studies. *Can J Cardiol* 2017;33(12):1624-32.
74. Il'yasova D, Scarbrough P, Spasojevic I. Urinary biomarkers of oxidative status. *Clin Chim Acta* 2012;413(19-20):1446-53.
75. Svingen GF, Schartum-Hansen H, Pedersen ER, et al. Prospective Associations of Systemic and Urinary Choline Metabolites with Incident Type 2 Diabetes. *Clin Chem* 2016;62(5):755-65.
76. Walford GA, Ma Y, Clish C, et al. Metabolite Profiles of Diabetes Incidence and Intervention Response in the Diabetes Prevention Program. *Diabetes* 2016;65(5):1424-33.
77. Topp H, Fusch G, Schöch G, et al. Noninvasive markers of oxidative DNA stress, RNA degradation and protein degradation are differentially correlated with resting metabolic rate and energy intake in children and adolescents. *Pediatr Res* 2008;64(3):246-50.
78. Sekhar RV, McKay SV, Patel SG, et al. Glutathione synthesis is diminished in patients with uncontrolled diabetes and restored by dietary supplementation with cysteine and glycine. *Diabetes Care* 2011;34(1):162-7.
79. Grizales AM, Patti ME, Lin AP, et al. Metabolic Effects of Betaine: A Randomized Clinical Trial of Betaine Supplementation in Prediabetes. *J Clin Endocrinol Metab* 2018;103(8):3038-49.

80. Bain MA, Faull R, Fornasini G, et al. Accumulation of trimethylamine and trimethylamine-N-oxide in end-stage renal disease patients undergoing haemodialysis. *Nephrol Dial Transplant* 2006;21(5):1300-4.
81. Chen YM, Liu Y, Zhou RF, et al. Associations of gut-flora-dependent metabolite trimethylamine-N-oxide, betaine and choline with non-alcoholic fatty liver disease in adults. *Sci Rep* 2016;6:19076.
82. Ge X, Zheng L, Zhuang R, et al. The Gut Microbial Metabolite Trimethylamine N-Oxide and Hypertension Risk: A Systematic Review and Dose-Response Meta-analysis. *Adv Nutr* 2020;11(1):66-76.
83. Heianza Y, Ma W, Manson JE, et al. Gut Microbiota Metabolites and Risk of Major Adverse Cardiovascular Disease Events and Death: A Systematic Review and Meta-Analysis of Prospective Studies. *J Am Heart Assoc* 2017;6(7)
84. Schiattarella GG, Sannino A, Toscano E, et al. Gut microbe-generated metabolite trimethylamine-N-oxide as cardiovascular risk biomarker: a systematic review and dose-response meta-analysis. *Eur Heart J* 2017;38(39):2948-56.
85. Yang S, Li X, Yang F, et al. Gut Microbiota-Dependent Marker TMAO in Promoting Cardiovascular Disease: Inflammation Mechanism, Clinical Prognostic, and Potential as a Therapeutic Target. *Front Pharmacol* 2019;10:1360.
86. Yao ME, Liao PD, Zhao XJ, et al. Trimethylamine-N-oxide has prognostic value in coronary heart disease: a meta-analysis and dose-response analysis. *BMC Cardiovasc Disord* 2020;20(1):7.
87. Aragonès G, González-García S, Aguilar C, et al. Gut Microbiota-Derived Mediators as Potential Markers in Nonalcoholic Fatty Liver Disease. *Biomed Res Int* 2019;2019:8507583.
88. Bouatra S, Aziat F, Mandal R, et al. The human urine metabolome. *PLoS One* 2013;8(9):e73076.
89. Palau-Rodriguez M, Tulipani S, Isabel Queipo-Ortuño M, et al. Metabolomic insights into the intricate gut microbial-host interaction in the development of obesity and type 2 diabetes. *Front Microbiol* 2015;6:1151.
90. Psychogios N, Hau DD, Peng J, et al. The human serum metabolome. *PLoS One* 2011;6(2):e16957.
91. Serena C, Ceperuelo-Mallafré V, Keiran N, et al. Elevated circulating levels of succinate in human obesity are linked to specific gut microbiota. *Isme j* 2018;12(7):1642-57.
92. Vojinovic D, Radjabzadeh D, Kurilshikov A, et al. Relationship between gut microbiota and circulating metabolites in population-based cohorts. *Nat Commun* 2019;10(1):5813.
93. Awoyemi A, Trøseid M, Arnesen H, et al. Markers of metabolic endotoxemia as related to metabolic syndrome in an elderly male population at high cardiovascular risk: a cross-sectional study. *Diabetol Metab Syndr* 2018;10:59.
94. Barengolts E, Green SJ, Chlipala GE, et al. Predictors of Obesity among Gut Microbiota Biomarkers in African American Men with and without Diabetes. *Microorganisms* 2019;7(9).
95. Citronberg JS, Curtis KR, White E, et al. Association of gut microbial communities with plasma lipopolysaccharide-binding protein (LBP) in premenopausal women. *Isme j* 2018;12(7):1631-41.
96. Liu X, Lu L, Yao P, et al. Lipopolysaccharide binding protein, obesity status and incidence of metabolic syndrome: a prospective study among middle-aged and older Chinese. *Diabetologia* 2014;57(9):1834-41.
