# Supplementary material for: Empowering consumers to PREVENT diet-related diseases through OMICS sciences (PREVENTOMICS): protocol for a parallel double-blinded randomised intervention trial to investigate biomarker-based nutrition plans for weight loss
Source: BMJ Open. 2022 Mar 29;12(3):e051285. doi: 10.1136/bmjopen-2021-051285 (PMC8966553; doi:10.1136/bmjopen-2021-051285)
Supplement: Supplementary data [file bmjopen-2021-051285supp002.pdf]

SUPPLEMENTARY ONLINE MATERIAL 2

Table 1: Target daily amounts of macronutrients and functional ingredients in meals provided to the personalised plan and control groups

		Personalised plan cluster				
**Meal: Breakfast + dinner**	**Control**	**Carbohydrate**	**Microbiota**	**Lipid**	**Inflammation**	**Oxidative stress**
Energy, kcal	1200 - 1500	1200 - 1500	1200 - 1500	1200 - 1500	1200 - 1500	1200 - 1500
Protein, %	10 - 14	10 - 14	10 - 14	10 - 14	10 - 14	10 - 14
Carbohydrate, %	45 - 55	45 - 55	45 - 55	45 - 55	45 - 55	45 - 55
Dietary fibre, g	22 - 28	42 – 52*	47 – 52*	22 - 28	22 - 28	22 - 28
Added sugars, g	0	0	0	0	0	0
Fat, %	30 - 40	30 - 40	30 - 40	30 - 40	30 - 40	30 - 40
**Functional ingredient**						
Fructooligosaccharides and/or Inulin		20 g	20 g			
Sunflower oil				20 g		
Turmeric powder					6-8 g	
Oleic acid enriched sunflower oil						20 g

Values are presented in ranges (minimum-maximum) for both genders.
**Including the functional ingredient*

Aldubayan MA, *et al. BMJ Open* 2022; 12:e051285. doi: 10.1136/bmjopen-2021-051285

Table 2: Example of 3-day menus provided to the personalised plan and control groups during the 10-week intervention

				Substituted or added ingredient in each cluster of the personalised plan group				
**Day**	**Breakfast meal**	**Ingredient**	**Control (female/male)**	**Carbohydrate**	**Microbiota**	**Lipid**	**Inflammation**	**Oxidative stress**
**1**	• You Go Ghurt with granola<br>• Kale salad with orange and roasted walnuts<br>• Cucumber and celery with pumpkin seeds juice	Yogurt, You Go Ghurt	160/210 g					
		Roasted walnuts	8/10 g			Not roasted	Not roasted	Not roasted
		Granola	20/25 g	+ 5 g FOS	+ 5 g FOS			
		Orange	1 piece/medium					
		Kale	40 g				100 g coleslaw	150 g red bell pepper
		Cucumber and celery with pumpkin seeds juice	250 mL				+3 g turmeric	
**2**	• Quinoa porridge with baked strawberry compote<br>• Blood orange with pine nuts and chervil<br>• Yogurt smoothie with Mint	Quinoa porridge	40/60 g	35/55 g + 10 g inulin	35/55 g + 10 g inulin	35/55 g + 5 g seed mixture	35/55 g + 5 g seed mixture	
		Oat milk with hibiscus	130/180 mL			+ 5 g sunflower oil		+ 5 g Oleic acid enriched sunflower oil
		Strawberry compote	40 g	30 g + 10 g FOS	30 g + 10 g FOS	+5 g sunflower oil		+5 g oleic oil
		Roasted pine nuts	5 g			Not roasted	Not roasted	Not roasted
		Chervil	5 g					
		Blood orange	1 piece/medium					
		Yogurt smoothie with Mint	250 mL					
**3**	• Pearl barley salad with peanuts vinaigrette topped with roasted peanuts + 1 pear<br>• Yogurt with baked blueberries<br>• Raspberry/chili/and violet cabbage juice	Roasted peanuts	6 g			Not roasted	8 g dark chocolate 70%	8 g dark chocolate 70%
		Mint	5 g					
		Lime	20 g					
		Yogurt/You Go Ghurt	70 g					
		Baked blueberries	10 g					
		Peanut vinaigrette	18/25 g			9/13 g sunflower oil	+ 3 g turmeric	9/13 g oleic oil
		Boiled pearl barley	80/110 g					
		Pear	1 piece/medium	100 g kale	100 g kale	100 g kale		
		Raspberry/chili and violet cabbage juice	250 mL					

Continued.

Concluded.

Day	Dinner meal	Ingredients	Control (female/male)	Personalised plan substituted ingredient in each cluster*				
				Carbohydrate	Microbiota	Lipid	Inflammation	Oxidative stress
**1**	Vegetarian massaman curry with cilantro/quinoa and baked tomatoes	Massaman curry	230/325 g	220/315 g with 118 g sweet potato + 10 g inulin	220/315 g + 10 g inulin		With 122 g sweet potatoes + 5 g turmeric	Extra baked carrots - 70 g
		Rapeseed oil	1 teaspoon					
		Sesame oil dressing	20 g			6.7 g sunflower oil		6.7 Oleic oil
		Sesame seeds	4 g					
		Baby spinach	5 g					
		Cilantro	5 g					
		Cooked quinoa	110/150 g			105/145 g + 5 g seed mixture	+5 g seed mixture	
		Tomato	90 g					
		Bell pepper/red	1 piece/medium	+30 g kale	+30 g kale	+20 g kale		
**2**	Baked eggplant with green hummus/bulgur salad and Turkish flatbread	Baked eggplant	65/75 g					
		Sunflower oil	1 teaspoon					
		Flatbread with nigella sativa	120/150 g			+3 g hemp seeds	+4 g turmeric	+3 g hemp seeds
		Green hummus	60/80 g	55/75 g + 5 g inulin	55/75 g + 5 g inulin	6/8 g sunflower oil		6/8 g oleic oil
		Mint cream	40 g					
		Lemon dressing	20 g	20 g lemon dressing with 5 g FOS (replaces sirup)	20 g lemon dressing with 5 g FOS (replaces sirup)	10 g sunflower oil (replaces the canola/olive oil in standard recipe)		10 g oleic oil (replaces the canola/olive oil in standard recipe)
		Parsley	5 g					
		Bulgur	30/45 g					
		Tomato	100 g					
		Courgette	150 g	150 g red cabbage	150 g red cabbage	150 g red cabbage		120 g carrot
**3**	Trofie pasta with cauliflower sauce and fried pak-choi + green bell pepper	Cauliflower sauce	140/190 g	135/185 g + 10 g FOS	135/185 g + 10 g FOS			
		Extra virgin olive oil	2.5 g					
		Mint	5 g					
		Marinated split peas	60/80 g				4 g turmeric + 4 g hemp seed	
		Lime dressing	30 g					
		Pak-choi	90 g	100 g broccoli	100 g broccoli	100 g broccoli		100 g Chinese cabbage
		Bell pepper/green	½ piece (100 g)	75 g kale	75 g kale	75 g kale		150 g red bell pepper
		Pasta/Trofie	120/150 g	Whole grain linguine	Whole grain linguine	Whole grain linguine	Whole grain linguine	Whole grain linguine

**Added or substituted ingredients from the control diet.*
Values are presented as 'female/male' unless equal for both genders.
Abbreviations: FOS, fructooligosaccharides.

Table 3: Nutritional information of the 3-days menus

Day 1, Breakfast	Control	Carbohydrate	Microbiota	Lipid	Inflammation	Oxidative stress
Energy, kcal	507/601	505/596	515/609	507/601	510/604	529/623
Protein, g (%)	14 (11)/16 (10)	13 (10)/16 (10)	14 (10)/16 (10)	14 (11)/16 (10)	13 (10)/15 (10)	14 (10)/16 (10)
Carbohydrate, g (%)	55 (43)/62 (41)	54 (42)/61 (40)	56 (43)/63 (41)	55 (43)/62 (41)	56 (44)/63 (42)	60 (45)/67 (43)
Dietary fibre, g	13/14	17/18	17/18	13/14	13/14	13/14
Sugars, g	29/31	28/29	29/31	29/31	29/30	35/35
Added sugars, g	0	0	0	0	0	0
Fat, g (%)	24 (41)/30 (44)	24 (41)/30 (44)	24 (41)/30 (43)	24 (41)/30 (44)	24 (41)/31 (44)	24 (41)/31 (44)
Saturated fat, g	4/5	4/5	4/5	4/5	4/5	4/5
Monounsaturated fat, g	3	3/4	3	3	3	3
Polyunsaturated fat, g	6/7	6/7	6/7	6/7	6/7	6/7
Salt, g	0.5	0.5	0.5	0.5	0.5	0.5
**Day 2, Breakfast**						
Energy, kcal	501/608	512/618	512/615	505/612	507/614	499/606
Protein, g (%)	12 (10)/15 (10)	12 (9)/15 (9)	12 (9)/15 (10)	12 (10)/15 (10)	13 (10)/16 (10)	12 (10)/15 (10)
Carbohydrate, g (%)	65 (53)/82 (55)	63 (50)/80 (52)	63 (50)/80 (52)	55 (44)/72 (48)	63 (50)/80 (53)	57 (46)/75 (49)
Dietary fibre, g	10/12	27/29	27/29	11/13	11/13	10/12
Sugars, g	22/24	24/33	22/24	20/23	22/24	21/23
Added sugars, g	0	0	0	0	0	0
Fat, g (%)	19 (33)/21 (31)	18 (31)/21 (29)	18 (31)/21 (29)	24 (42)/27 (38)	20 (35)/23 (33)	23 (41)/25 (37)
Saturated fat, g	3	3	3	4	3/4	3/4
Monounsaturated fat, g	2	2	1/2	3	2	3
Polyunsaturated fat, g	3	3	3	7/8	4/5	6/7
Salt, g	1	1	1	1	1	1
**Day 3, Breakfast**						
Energy, kcal	508/608	510/610	510/610	510/610	528/628	518/625
Protein, g (%)	9 (7)/10 (7)	13 (10)/15 (10)	13 (10)/15 (10)	13 (10)/15 (10)	8 (6)/10 (6)	8 (6)/10 (6)
Carbohydrate, g (%)	68 (53)/78 (52)	60 (48)/71 (47)	60 (48)/71 (47)	60 (48)/71 (47)	71 (54)/82 (52)	69 (53)/82 (54)
Dietary fibre, g	8.2/8.8	11/12	11/12	11/12	9.3/9.8	8.6/9.6
Sugars, g	29	21	21	21	31	31
Added sugars, g	0	0	0	0	0	0
Fat, g (%)	21 (37)/27 (38)	22 (38)/28 (39)	22 (38)/28 (39)	22 (38)/28 (39)	22 (37)/28 (39)	22 (37)/27 (38)

Continued.

Day 3, Breakfast	Control	Carbohydrate	Microbiota	Lipid	Inflammation	Oxidative stress
Saturated fat, g	3/4	3/4	3/4	3/4	5/6	5/6
Monounsaturated fat, g	5/6	5/6	5/6	5/6	4/6	4/6
Polyunsaturated fat, g	8/11	8/11	8/11	8/11	7/10	7/10
Salt, g	1	1	1	1	1	0.5
**Day 1, Dinner**						
Energy, kcal	697/881	712/890	724/907	725/908	729/907	697/881
Protein, g (%)	19 (11)/25 (11)	19 (11)/25 (11)	20 (11)/26 (11)	21 (11)/26 (12)	20 (11)/25 (11)	19 (11)/25 (11)
Carbohydrate, g	86 (50)/114 (52)	84 (48)/110 (50)	87 (48)/114 (50)	86 (48)/114 (50)	86 (48)/112 (50)	86 (50)/114 (52)
Dietary fibre, g	12/14	23/26	22/25	14/16	15/18	12/14
Sugars, g	24/28	27/33	25/29	24/28	27/32	24/28
Added sugars, g	0	0	0	0	0	0
Fat, g (%)	29 (36)/34 (34)	28 (35)/33 (33)	29 (35)/34 (33)	31 (37)/36 (35)	31 (37)/36 (35)	29 (36)/34 (34)
Saturated fat, g	7/9	7/9	7/9	7/9	8/10	7/9
Monounsaturated fat, g	7	7	7	7/8	7/8	7
Polyunsaturated fat, g	11/13	11/13	11/13	12/14	12/14	11/13
Salt, g	2/3	2/3	2/3	2/3	2/3	2/3
**Day 2, Dinner**						
Energy, kcal	704/871	731/897	731/897	742/912	718/884	742/901
Protein, g (%)	21 (12)/26 (12)	21 (11)/26 (12)	21 (11)/26 (12)	22 (12)/28 (12)	21 (12)/27 (12)	21 (11)/26 (12)
Carbohydrate, g	90 (54)/116 (51)	95 (52)/121 (54)	95 (52)/121 (54)	96 (52)/122 (53)	92 (51)/118 (54)	96 (52)/122 (54)
Dietary fibre, g	14/17	25/28	25/28	15/18	15/18	16/18
Sugars, g	5/6	11/12	11/12	11	5/6	12/13
Added sugars, g	0	0	0	0	0	0
Fat, g (%)	27 (33)/31 (30)	25 (30)/29 (28)	25 (30)/29 (28)	28 (32)/29 (31)	27 (33)/31 (30)	28 (33)/32 (30)
Saturated fat, g	3/4	3/4	3/4	3/4	4	4
Monounsaturated fat, g	12/15	12/14	12/14	13/15	12/15	13/15
Polyunsaturated fat, g	8/9	7/8	7/8	9/10	8/9	9/10
Salt, g	3/4	3/4	3/4	3/4	3/4	3/4
**Day 3, Dinner**						
Energy, kcal	699/877	731/909	731/909	722/900	732/911	723/901
Protein, g (%)	23 (13)/28 (13)	25 (14)/31 (14)	25 (14)/31 (14)	25 (14)/31 (14)	24 (13)/30 (13)	24 (13)/30 (13)
Carbohydrate, g (%)	105 (60)/131 (60)	106 (58)/131 (58)	106 (58)/131 (58)	106 (58)/131 (59)	107 (59)/133 (58)	109 (60)/135 (61)

Continued.

Aldubayan MA, *et al. BMJ Open* 2022; 12:e051285. doi: 10.1136/bmjopen-2021-051285

Concluded.

Day 3, Dinner	Control	Carbohydrate	Microbiota	Lipid	Inflammation	Oxidative stress
Dietary fibre, g	11/13	22/25	22/25	14/16	12/14	11/14
Sugars, g	20/23	19/22	19/22	20/23	20/23	24/27
Added sugars, g	0	0	0	0	0	0
Fat, g (%)	19 (24)/24 (24)	19 (22)/25 (23)	19 (22)/25 (23)	19 (23)/25 (24)	21 (25)/26 (25)	19 (23)/25 (24)
Saturated fat, g	3/4	3/4	3/4	3/4	3/4	3/4
Monounsaturated fat, g	7/10	7/9	7/9	7/10	8/10	7/9
Polyunsaturated fat, g	6/8	6/8	6/8	7/9	7/9	6/8
Salt, g	2/3	2/3	2/3	2/3	2/3	2/3

Values are presented as 'female/male' unless equal for both genders.

Aldubayan MA, *et al. BMJ Open* 2022; 12:e051285. doi: 10.1136/bmjopen-2021-051285
