# Supplementary material for: Empowering consumers to PREVENT diet-related diseases through OMICS sciences (PREVENTOMICS): protocol for a parallel double-blinded randomised intervention trial to investigate biomarker-based nutrition plans for weight loss
Source: BMJ Open. 2022 Mar 29;12(3):e051285. doi: 10.1136/bmjopen-2021-051285 (PMC8966553; doi:10.1136/bmjopen-2021-051285)
Supplement: Supplementary data [file bmjopen-2021-051285supp003.pdf]

SUPPLEMENTARY ONLINE MATERIAL 3: informed consent version 2.0, 18 September 2020

Title of research project: **PREVENTOMICS: Empowering consumers to PREVENT diet-related diseases through OMICS-based personalized nutrition**

Consent from the study participant:

I have obtained written and oral information about the research project and I am informed about the aim, methods, benefits and risks of participating in the study.

I know that it is completely voluntary to participate, and I have the right to withdraw the informed consent at any time and with no loss of benefits to which I am otherwise entitled.

I consent to participate in the research project and that my biological material will be stored in a research biobank. I have received a copy of this informed consent form as well as a copy of the written information.

Study participant name: ____

Date: ________________ Signature: ____

In case new information that has substantial influence on your health emerges from the research project, you will be informed. Would you prefer **not** to be informed about information that has substantial influence on your health, please mark it here _________ (insert X).

Do you wish to be informed about the final result of the research project and the potential consequences for you:

Yes _____ (insert X) No _____ (insert X)

Consent from the study staff that provided the oral information:

I declare, that the participant has received both written and oral information about the research project.

I declare to the best of my knowledge and belief that the participant has received sufficient information to decide to participate in the research project.

Study staff name: ____

Date: ________________ Signature: ____

National project identification: H-20029882
