# Supplementary material for: Empowering consumers to PREVENT diet-related diseases through OMICS sciences (PREVENTOMICS): protocol for a parallel double-blinded randomised intervention trial to investigate biomarker-based nutrition plans for weight loss
Source: BMJ Open. 2022 Mar 29;12(3):e051285. doi: 10.1136/bmjopen-2021-051285 (PMC8966553; doi:10.1136/bmjopen-2021-051285)
Supplement: Author's manuscript [file bmjopen-2021-051285.draft_revisions.pdf]

BMJ Open is committed to open peer review. As part of this commitment we make the peer review history of every article we publish publicly available.

When an article is published we post the peer reviewers' comments and the authors' responses online. We also post the versions of the paper that were used during peer review. These are the versions that the peer review comments apply to.

The versions of the paper that follow are the versions that were submitted during the peer review process. They are not the versions of record or the final published versions. They should not be cited or distributed as the published version of this manuscript.

BMJ Open is an open access journal and the full, final, typeset and author-corrected version of record of the manuscript is available on our site with no access controls, subscription charges or pay-per-view fees (http://bmjopen.bmj.com).

If you have any questions on BMJ Open's open peer review process please email info.bmjopen@bmj.com

BMJ Open

Empowering consumers to 'PREVENT' diet-related diseases through 'OMICS' sciences (PREVENTOMICS) trial: study protocol for a randomized, parallel, double-blinded, intervention study to investigate biomarker-based nutrition plans for weight loss.

Journal:	*BMJ Open*
Manuscript ID	bmjopen-2021-051285
Article Type:	Protocol
Date Submitted by the Author:	16-Mar-2021

Complete List of Authors:	Aldubayan, Mona A.; University of Copenhagen Faculty of Science, Nutrition, Exercise and Sports; King Saud bin Abdulaziz University for Health Sciences, College of Applied Medical Sciences<br>Pigsborg, Kristina; University of Copenhagen Faculty of Science, Nutrition, Exercise and Sports<br>Gormsen, Sophia; Simple Feast<br>Serra, Francisca; University of the Balearic Islands, Laboratory of Molecular Biology, Nutrition and Biotechnology - NUO group; Alimentómica S.L., Spin-off n.1 of the University of the Balearic Islands<br>Palou, Mariona; University of the Balearic Islands, Laboratory of Molecular Biology, Nutrition and Biotechnology - NUO group; Alimentómica S.L., Spin-off n.1 of the University of the Balearic Islands<br>Mena, Pedro; University of Parma, Department of Food and Drug, Human Nutrition Unit<br>Wetzels, Mart; Onmi: Behaviour Change Technology<br>Calleja , Alberto; Grupo Carinsa<br>Caimari, Antoni; Eurecat Centre Tecnològic de Catalunya, Biotechnology Area, Nutrition and Health Unit<br>Del Bas, Josep; Eurecat Centre Tecnològic de Catalunya, Biotechnology Area, Nutrition and Health Unit<br>Gutierrez, Biotza; Eurecat Centre Tecnològic de Catalunya, Biotechnology Area, Nutrition and Health Unit<br>Hjorth, Mads F.; University of Copenhagen Faculty of Science, Nutrition, Exercise and Sports

Keywords:	NUTRITION & DIETETICS, PUBLIC HEALTH, GENETICS

SCHOLARONE™
Manuscripts

UNIVERSITY OF COPENHAGEN

FACULTY OF SCIENCE

DEPARTMENT OF NUTRITION, EXERCISE AND SPORTS

Title: Empowering consumers to 'PREVENT' diet-related diseases through 'OMICS' sciences (PREVENTOMICS) trial: study protocol for a randomized, parallel, double-blinded, intervention study to investigate biomarker-based nutrition plans for weight loss.

Author names:

Mona A. Aldubayan[1,2], Kristina Pigsborg[1], Sophia M. O. Gormsen[3], Francisca Serra[4], Mariona Palou[4], Pedro Mena[5], Mart Wetzels[6], Alberto Calleja[7], Antoni Caimari[8], Josep M del Bas[8], Biotza Gutierrez[8], Mads F. Hjorth[1]

Corresponding author:

Mona Adnan Aldubayan
Department of Nutrition, Exercise and Sports,
Faculty of Science, University of Copenhagen
Rolighedsvej 26, 1958 Frederiksberg, Denmark
Phone: +45 91991923
E-mail: monal@nexs.ku.dk

Author affiliations:

[1]Department of Nutrition, Exercise and Sports, Faculty of Science, University of Copenhagen, Denmark.

[2]King Saud bin Abdulaziz University for Health Sciences, Riyadh, Saudi Arabia.

[3]Simple Feast, Copenhagen, Denmark.

[4]Laboratory of Molecular Biology, Nutrition and Biotechnology; NUO group. Universitat de les Illes Balears, Palma, Spain; Institut d'Investigació Sanitària Illes Balears (IdISBa), Palma, Spain; CIBER de Fisiopatología de la Obesidad y Nutrición (CIBEROBN), Madrid, Spain and Alimentómica S.L., Spin-off n.1 of the University of the Balearic Islands, Palma, Spain.

[5]Human Nutrition Unit, Department of Food and Drug, University of Parma, Parma, Italy.

[6]ONMI: Behaviour Change Technology, Eindhoven, Netherland.

[7]Grupo Carinsa, Sant Quirze del Valles, Barcelona, Spain.

[8]Eurecat, Centre Tecnològic de Catalunya, Biotechnology Area, Nutrition and Health Unit, Reus, Spain.

Word count: 4,518 (excluding title page, abstract, references, figures and tables).

For peer review only

ABSTRACT

Introduction: Personalized nutrition holds immense potential for preventing and treating diet-related diseases, such as obesity, over a conventional one-size-fits-all approach. The current study aims to examine whether a personalized nutritional plan is able to produce more favorable health outcomes than a standard approach based on general dietary recommendations in subjects with elevated waist circumference.

Methods and analysis: The study is a 10-week randomized, parallel, double-blinded, intervention study. We plan to include 100 adults aged 18-65 years interested in losing weight, with body mass index (BMI) ≥27 and <40 kg/m^2 and elevated waist circumference (men >94.0 cm; women >80.0 cm). Participants will be randomized into one of two groups: (1) personalized plan group will be split into five predefined clusters based on their metabolic biomarkers and genetics analysis and will receive meals accordingly. In addition, participants will be enrolled in a personalized behavioral change program via electronic push notifications; (2) control group will receive meals following general dietary recommendations as well as electronic behavioral prompts, but not in a personalized fashion. The primary outcome focuses on body fat mass differences between the two groups from baseline to the end of the intervention (week 10). Other outcome measures include changes in body weight and composition, lipid profile, fasting blood glucose, adipokines, inflammatory biomarkers, blood pressure, physical activity and sleep patterns, health-related quality of life, eating behavior, attitude to weight management diets and dietary intake. The effect of the intervention on the primary outcome will be analyzed by means of linear mixed models including the stratification variable as well as sex, age and BMI at baseline.

Ethics and dissemination: The protocol has been approved by the Ethics Committee of the Capital Region, Copenhagen, Denmark. Study findings will be disseminated widely through peer-reviewed publications and conference presentations.

Trial registration number: ClinicalTrials.gov registry (NCT04590989).

Keywords: Personalized nutrition, Precision nutrition, Nutrigenomics, Nutrigenetics, Metabolomics, Obesity, Overweight, Weight management, Body weight

For peer review only - http://bmjopen.bmj.com/site/about/guidelines.xhtml

Strengths and limitations of this study

- Applying state-of-the-art knowledge that integrates metabolomics and genetics with nutrition, the study may identify novel approaches in supporting weight loss and lasting health-promoting behaviors.
- The study is double blinded which is rarely seen in nutritional science and serves as proof of concept for personalized dietary management of obesity.
- A potential limitation is that both groups are receiving healthy foods and behavioral advices, despite not personalized in control, it may mask the proposed personalized intervention effect.
- The study is powered to detect differences in 10-week body fat loss between intervention and control and not within each of the five clusters which will only be post-hoc analysis.
- Potential long-term effects of a personalized approach cannot be evaluated from this 10-week study; however, it can constitute an interesting approach for implementation in longer obesity-management programs.

For peer review only - http://bmjopen.bmj.com/site/about/guidelines.xhtml

INTRODUCTION

The ultimate goal of nutritional research and recommendations focuses on promoting human health and preventing or treating chronic diseases.[1] Yet, the global prevalence rate of nutrition-related noncommunicable diseases (NCDs) continues to rise rapidly over the recent decades.[2] Importantly, evidence shows that obesity is considered a major risk factor for developing these NCDs including type 2 diabetes, certain types of cancers and cardiovascular diseases which are considered the leading causes of morbidity and mortality.[3 4] Therefore, obesity can place a great burden on the individual, the healthcare systems and the society.[3] In this vein, enormous efforts from health professionals to tackle this epidemic have been implemented through setting different policies and guidelines for the public, but with little success as management of such multifactorial condition remains a very challenging task. Moreover, researchers have long debated the optimal diet characteristics that can be most effective in reducing excess weight gain such as comparing diets varying in macronutrient composition (e.g. low carbohydrate, low fat diet, high protein).[5 6] Several clinical trials have demonstrated that certain individuals have benefited more from a particular dietary intervention than others in reducing their weight, while even few were able to keep those pounds off over the long-term.[7 8] This implies that there is no strong evidence that one diet is superior to others for inducing weight loss and no such thing as “perfect” diet for everyone. Such substantial inter-individual variation in response to various dietary approaches can be attributed to the phenotypic factors and genetic variants which influence how the body utilizes and metabolizes nutrients.[7] This gives rise to the demand for customizing nutritional plans and advices at the individual level rather than at a population-based perspective. Recent developments in “omics” technologies (nutrigenomics, transcriptomics, epigenomics, metabolomics, metagenomics, etc.) offered opportunities to explore the complex interplay between nutrition, genetics and metabolism.[9] By integrating these novel tools with bioinformatics in research, the potential of “personalized nutrition” can be implemented through identifying novel biomarkers utilized to predict the most effective diet for weight loss and improving nutritional status.[9-11] Therefore, the ability to give evidence-based dietary advices based on individual's genetic make-up, phenotypic information on anthropometry, biochemical and metabolic analysis, physical activity, medical history, among other things can be more impactful in changing behavior and ultimate health outcomes.

In this context, H2020 PREVENTOMICS (Empowering consumers to PREVENT diet-related diseases through OMICS sciences), coordinated by Eurecat in Spain, has developed a platform with a Decision Support System (DSS) tool that integrates individuals’ phenotypic characteristics at the metabolomic level with their genotype, lifestyle habits and preferences to improve their health status through developing personalized

For peer review only - http://bmjopen.bmj.com/site/about/guidelines.xhtml

nutrition plans. The project aims to examine the validity of PREVENTOMICS platform in terms of its potential for personalization at different levels of the food value chain. This will be achieved through three different interventional studies taking place in 1) Denmark, 2) Spain, and 3) Poland and the United Kingdom, with both healthy volunteers and volunteers with abdominal obesity. Here we deal with the specific characteristics of the Danish study protocol.

Research hypothesis and aims

The over-all aim of this 10-week randomized-controlled study, is to examine the validity of the PREVENTOMICS platform integrated in an e-commerce digital tool created for delivering personalized meals for the purpose of producing more favorable health outcomes than meals based on general dietary recommendations in overweight subjects with elevated waist circumference. In addition, the intervention group will receive personally tailored and actionable behavior change prompts whereas the control group receives general advices. Our hypothesis is that personalized dietary plan along with the behavioral change prompts will produce greater reduction in fat mass and weight, as well as promoting favorable changes in blood metabolic and inflammatory biomarkers compared to control group.

Primary and secondary objective:

The primary objective of this study is to evaluate the change in body fat mass between the personalized plan group and the control group over the 10-week intervention period. The secondary objective is to evaluate the change of the following health outcomes between the personalized and control group: (1) body composition (visceral and subcutaneous fat, body lean mass, weight, body mass index, waist circumference); (2) lipid profile (total, LDL and HDL cholesterol, triglycerides); (3) glucose homeostasis (blood glucose, insulin, HOMA-IR); (4) inflammatory markers (hs-CRP, IL-6, MCP1, TNFα, IL-10, soluble ICAM1, soluble CD14); (5) adipokines including leptin and adiponectin; (6) liver health markers (ALT, GGT); (7) renal health markers (uric acid, creatinine); and (8) blood pressure.

METHODS AND ANALYSIS

Study design

This is a randomized, single-center, parallel-group (1:1 ratio), double-blinded intervention study to be conducted at the research facilities of the Department of Nutrition, Exercise and Sports (NEXS), University of Copenhagen, Denmark. The study protocol adheres to the Standard Protocol Items: Recommendations for Interventional Trials (SPIRIT) guidelines.[12] This study is registered at ClinicalTrials.gov in October 2020 (NCT04590989) and subsequently recruitment started at NEXS in October (week 43) 2020. Information

For peer review only - http://bmjopen.bmj.com/site/about/guidelines.xhtml

needed to determine the metabolic cluster of participants were collected in January (week 2-5) 2021, the actual analysis of biological samples and clustering will be done in February and March 2021 (week 6-10). The 10-week intervention period will run from March (week 11-12) to June (week 22-23) 2021. All data collected is expected to be fully analyzed by the end of 2021. The overall study design is illustrated in figure 1.

Patient and Public Involvement: Patients and the public were not involved in the design, conduct or reporting of this study.

Study participants

Participants included will be male and female volunteers, aged 18–65 years with a BMI of ≥27.0–<40.0 kg/m^2 and elevated waist circumference (men >94.0 cm; women >80.0 cm). Furthermore, participants should possess a smartphone and be able to provide an informed consent. The exclusion criteria are as follows: (1) diagnosis of diabetes; (2) history or diagnosis of heart, liver or kidney disease; (3) chronic diseases, e.g. cancer within the past 5 years (except adequately-treated localized basal cell skin cancer); (4) use of drugs (e.g. antibiotics), that in the opinion of the medically responsible investigator, are likely to affect the primary outcomes of the study; (5) being lactating, pregnant or planning to become pregnant within the study period; (6) self-reported weight change of >5 % within two months prior to screening; (7) participation within another clinical trial; (8) other blood donation during the study; (9) having allergies or food intolerances; (10) no or limited access to the Internet. Participants unable to comply with the study protocol, judged by the investigator, will also be excluded.

Recruitment procedure

Study flow chart is summarized in figure 2. Potential participants were recruited through internet-based advertisements. Trained study personnel contacted 220 subjects interested in the study via telephone to determine initial eligibility in accordance to the inclusion/exclusion criteria (pre-screening). Written information about the study were provided to 120 potential participants who were deemed eligible from the telephone pre-screening, and scheduled for an oral information meeting (V0) at the department. If the participant signed the informed consent, either immediately following the information meeting or after days of consideration, they were screened according to the inclusion/exclusion criteria to assess eligibility along with obtaining medical history. A total of 106 participants were recruited and invited for V1 where anthropometric measurements, blood, saliva, and urine samples as well as filling various questionnaires were obtained. One hundred participants completed V1 and have their samples sent to the assigned consortium for analyzing data on subjects' metabolome and genotype in addition to lifestyle habits,

For peer review only - http://bmjopen.bmj.com/site/about/guidelines.xhtml

preferences and physiological status. These data will be utilized to determine subjects' metabolic cluster (see later) and develop the personalized dietary plans for the subsequent 10-week intervention period.

Randomization and concealment

Eligible participants will be randomly allocated to either intervention group (personalized plan) or control group prior to commencement of the trial. The allocation will be computer generated, and stratified by metabolic clusters (oxidative stress; inflammation; carbohydrate metabolism; lipid metabolism; microbiota-generated metabolites) in a 1:1 randomization between control and intervention groups. The person responsible for generating the code will not take part in the inclusion and examination of study participants. Study staff at University of Copenhagen and participants will be blinded to the treatment group and analyses of results will be performed in a blinded fashion too. Moreover, the statistical analyses of the main outcome variable will be done without breaking the code for the intervention treatment until the primary analyses are finalized.

Interventions

1. Dietary intervention:

During the 10-week study, control and personalized plan (PP) group will receive easy-to-prepare meal boxes twice a week from Simple Feast complying with the general dietary recommendations of macronutrient distribution. Each delivery will provide meal boxes of breakfast and dinner for the subsequent three days (12 meals/week). Energy of meals for all participants is calculated based on the average requirements of energy consumption for the general population, which is 2000 kcal/day for women and 2500 kcal/day for men.[13] This will enable a negative energy balance in the participants with obesity. As 25% and 35% of daily energy is commonly consumed at breakfast and dinner, respectively, meals for both intervention-groups will provide approximately 500-625 kcal/day for breakfast and 700-875 kcal/day for dinner. Food provided by Simple Feast are organic-vegetarian, however, participants are allowed to eat non-vegetarian foods in the meals not provided.

Personalized plan group

Cluster allocation

All subjects will be categorized into one of five metabolic clusters (table 1) based on their metabolic and genetic biomarkers. The randomization of subjects into the PP and control group will be stratified according to this metabolic cluster, and only subjects in PP group will be allocated into one of these metabolic clusters during the intervention. The metabolic clusters will be derived in the following manner. First,

For peer review only - http://bmjopen.bmj.com/site/about/guidelines.xhtml

collected samples of urine, plasma and serum will be analyzed in order to assess a total of 58 biomarkers used to define the five metabolic processes: 1) oxidative stress; 2) inflammation; 3) carbohydrate metabolism; 4) lipid metabolism; and 5) microbiota-generated metabolites. References for biomarkers listed in table 1 are included in online supplemental material 1.

**Carbohydrate**	**Lipid**	**Inflammation**	**Oxidative stress**	**Microbiota**
Glucose	LDL-cholesterol	C-reactive protein (CRP)	8-iso-prostaglandin F2 alpha (8-iso-PGF2α)	Trimethylamine (TMA)
HOMA-IR	Total cholesterol	N-acetylglycoproteins	8-hydroxy 2-deoxyguanosine (8-OHdG)	Trimethylamine N-oxide (TMAO)
Glutamate	Total polyunsaturated fatty acids (PUFAs)	Monocyte chemoattractant protein (MCP-1)	Oxidized low density lipoprotein (LDLox)	Betaine
Uric acid	HDL-cholesterol	Tumor necrosis factor alpha (TNFα)	Uric acid	Choline
Leptin	Saturated fatty acids (SFAs)	Interleukin-6 (IL-6)	Allantoin	Dimethylamine (DMA)
Adiponectin	Triglycerides	Interleukin-10 (IL-10)	Betaine	Dimethylglycine
Insulin	Total monounsaturated fatty acids (MUFAs)	Saturated fatty acids (SFAs)	Pseudouridine	Lipopolysacchari de binding protein (LBP)
Tyrosine	Total lysophosphatidylcholines (LPC)	Soluble intercellular adhesion molecule-1 (sICAM-1)	Dimethylglycine	Succinate
Propionylcarnitine	Linoleic acid	Total lysophosphatidylcholines (LPC)	Methionine	Lactate
Lactate	Docosahexaenoic acid (DHA)	Lipopolysaccharide binding protein (LBP)	Glycine	Acetate
Valine	Oleic acid	DHA C20:3		
Leucine	Choline	Soluble CD14 (sCD14)		
Isoleucine	3-hydroxybutyrate	Linoleic acid C18:2		
Phenylalanine	Propionylcarnitine	Polyunsaturated fatty acids (PUFAs)		
Glutamine	Adiponectin			
	Leptin			

For peer review only - http://bmjopen.bmj.com/site/about/guidelines.xhtml

Table 1: Full list of biomarkers in relation with the metabolic clusters. Some biomarkers help defining more than one cluster

For peer review only

For peer review only - http://bmjopen.bmj.com/site/about/guidelines.xhtml

Secondly, a core of 35 different single nucleotide polymorphisms (SNPs) associated with the five metabolic processes and able to modulate the biomarkers levels reported in table 1 have been identified by Alimentomica (Spain) and will be analyzed from the saliva samples (see table 2). The biomarkers of the lipid cluster are able to be modulated, at different degree, by a set of 18 SNPs involved in 14 genes[14-21], the carbohydrate cluster is represented by a panel of 12 SNPs related to 11 genes[15 16 22-25] and the inflammation cluster by 8 SNPs in 7 genes.[26-29] Concerning the oxidative stress cluster, parse scientific data deal with the genetic impact on the specific PREVENTOMICS biomarkers of this cluster. Therefore, the genetic risk score is composed by 7 genetic variants (6 genes) associated to reduced ability to buffer the oxidative stress associated with low levels of plasma antioxidant agents.[30-34] In relation with the microbiota cluster, current references are not providing enough level of scientific evidence to give any specific recommendation to support the role of SNPs (included in the panel or not). Evidence are not sufficiently characterized concerning the host genetic influence on microbiota response and/or associated with their metabolite production. No genetic influence in this cluster is adopted at present. Finally, these specific SNPs will in combination with the biomarkers in the five metabolic processes be used to calculate individual scores for each of the five metabolic clusters. The metabolic cluster with the highest score will be assigned to each subject. The specific algorithm cannot be disclosed due to intellectual property rights (IPR).

**Lipid**		**Carbohydrate**		**Oxidative**		**Inflammation**	
**Gene**	**SNP**	**Gene**	**SNP**	**Gene**	**SNP**	**Gene**	**SNP**
ADIPOQ	rs182052	ADIPOQ	rs182052	COMT	rs4680	APOE	rs429358
APOA5	rs12272004	ASCL1	rs17450122	CPS1	rs1047891	CADM3-AS1	rs12075
APOA5	rs662799	FADS1, FADS2	rs174550	CPS1	rs715	CUX1	rs409224
APOE	rs7412	GCKR	rs1260326	FGF21	rs838133	FADS1	rs174547
APOE	rs429358	GCKR	rs780093	GSTP1	rs1695	GCKR	rs1260326
CUX1	rs409224	GLS2, SPRYD4	rs2657879	MTHFR	rs1801133	GCKR	rs780093
FADS1	rs174547	LEP	rs10487505	SOD2	rs4880	ICAM1	rs5498
GCKR	rs780093	PPARG	rs1801282			IL-6	rs1800795
GCKR	rs1260326	SLC16A10	rs14399				
HFE	rs1800562	SLC16A9	rs1171614				
LEP	rs10487505	SLC2A2	rs8192675				
LPL	rs268	TCF7L2	rs7903146				
LPL	rs326						
PNPLA3	rs738409						
PPID	rs8396						
SLC16A9	rs1171614						
TIMP3	rs12678919						
TRIM58	rs3811444						

Table 2: List of SNPs in relation with the metabolic clusters.

eview only

For peer review only - http://bmjopen.bmj.com/site/about/guidelines.xhtml

Meals tailoring:

Eurecat Nutrition Team has prepared a list of recommended food items to increase as well as food to exclude or limit from the diet for each cluster to improve cluster's health status. The list will be adopted by Simple Feast in creating five different menus that will encompass the 12 meals/week for the five different metabolic clusters in the intervention group (n=50). In addition, the food will also include some bioactive compounds provided by CARINSA. Each participant assigned to one of the PP clusters will receive approximately 20 g of functional ingredient per day, except for inflammation cluster where they will receive 6-8 g per day (table 3). The macronutrient distribution between clusters will only differ in regard to the amount of fiber. The diets fiber content will be higher in the Carbohydrate and Microbiota clusters as they receive fructooligosaccharide (FOS) and inulin as the functional ingredient(s).

Cluster	Functional ingredient and<br>specification of recommended food that are predominant in the different clusters.
**Carbohydrate**	Functional ingredient: *FOS and †Inulin<br><br>Prebiotics: fiber-rich plants (Jerusalem artichoke, onion, leek, asparagus, kale)
**Microbiota**	Functional ingredient: *FOS and †Inulin<br><br>Prebiotics: fiber-rich plants (Jerusalem artichoke, onion, leek, asparagus, kale)<br>Fermented vegetables.<br>Vegetables rich in fiber.
**Lipid**	Functional ingredient: sunflower oil<br><br>Raw nuts and seeds.<br>Omega 3 and 6: chia seeds, hemp seeds, walnuts, flax seeds.<br>Vegetables rich in fiber.
**Inflammation**	Functional ingredient: turmeric powder<br><br>Raw nuts and seeds.<br>Omega 3 and 6: chia seeds, hemp seeds, walnuts, flax seeds.<br>Dark Chocolate
**Oxidative stress**	Functional ingredient: oleic acid enriched sunflower oil.<br><br>Raw nuts and seeds<br>Orange, yellow, red colored vegetables (rich in vitamin A, C, E).<br>Dark chocolate<br>Vegetables rich in fiber

*Table 3: functional ingredients from CARINSA. *Fructooligosaccharide originates from partial hydrolysis of chicory roots. †Inulin is extracted from chicory roots.*

As mentioned, the combination of the five metabolic processes (table 1) and the relevant genotype variants (table 2) determine the metabolic cluster of the participants which will be visible to Simple Feast to deliver the meals accordingly. Recipe recommendations for other meals that are not provided (e.g. Saturday +

For peer review only - http://bmjopen.bmj.com/site/about/guidelines.xhtml

lunch) will be presented through the Simple Feast Recipe App. Recipes are also complied with the nutritional recommendations list to restore the most compromised metabolic process of subjects.

Control intervention

The control group (n=50) will receive 12 meals/week from Simple Feast based on general dietary recommendations of macronutrient composition and suggestions for non-personalized recipes through Simple Feast Recipe App when meals are not provided (Saturday and lunches).

2. Behavioral assessment and intervention

All participants will be asked to fill out a behavioral questionnaire at V2, to provide information of certain actions or behaviors that affect the physical, emotional, or mental well-being of the subjects. Subjects in the PP group will receive electronic "Do's" as push notifications from the predefined ONMI's evidence-based behavioral change program, for the purpose of general behavioral change and improving adherence to the nutritional intervention.[35] The messages that will be sent during the 10-week period are personalized based on user reports from the behavioral questionnaire at V2 in addition to inputs from the nutritional recommendations of food to increase via the PREVENTOMICS platform. If someone for example is recommended to eat kale and brussels sprouts, they could get a message like: “Our analysis shows kale and brussels sprouts should be part of your diet, they are good for you. Find out how much you should be consuming. Do it now”. Subjects in control group will also be enrolled in the behavioral program, but the program will not be personalized nor based on the same behavior change methodology as in the PP group. In other words, the control group will get information more than is triggered to take actual action (i.e. general guidelines that is available from the National Health Service and World Health Organization).

Compliance and food intake biomarkers

Assessing dietary adherence will be done twice a week through an electronic questionnaire to report food consumed from the meals provided by Simple Feast in the previous three days. An example of such a question in the questionnaire would be: “How much of your breakfast did you eat on day 1?” The questions can be answered as follows: a) Nothing/very little (0-30%); b) Approximately half (30-70%); c) Almost everything/everything (70-100%). In addition, a six point Likert scale question that ranges from; 1 (not at all) to 6 (completely) will be included at the end of the intervention to measure an overall compliance of the diet and the Do’s messages. Moreover, objective measurements will be carried out to assess participants’ adherence to the nutritional recommendations. In particular, urine will be collected and analyzed for selected biomarkers of food intake through a target UPLC-IMS-HRMS approach at the

For peer review only - http://bmjopen.bmj.com/site/about/guidelines.xhtml

University of Parma (UNIPR, Italy). Information on biomarkers of food intake will also serve to assert the validity of the information collected through 3-day food diaries at V1 and V3.

Data collection

Data will be collected at screening, pre-baseline (V1), at baseline (V2), and 10 weeks after initiating the intervention (V3) as displayed in table 3. Data will be obtained from self-reported questionnaires, biospecimens collection, accelerometers, and physical examinations conducted by trained local research staff in accordance with the standard operating procedures (SOPs). All primary and secondary endpoints are based on measurements obtained from baseline and at the end of the intervention. Measurements taken pre-baseline are used for clustering assignment. Reminder text messages will be sent to the participants before visit days. It is expected that we will be able to maintain a high retention rate of participants due to the nature of the study. Table 4 outlines an overview of different data being collected throughout the trial.

Primary outcome:

The primary endpoint is the difference in body fat mass (kg) from baseline to end of trial between the two intervention groups. Body composition and body fat mass is evaluated by use of DXA scans (iDXA, Lunar Radiation Co., Madison, Wisconsin, USA) which is performed at V2 and V3. The participant is scanned in a fasted state, lying on their back wearing lightweight clothes without jewelry and other metallic objects.

For peer review only - http://bmjopen.bmj.com/site/about/guidelines.xhtml

	Screening visit (V0)	Pre-baseline visit (V1)	Baseline visit (V2)	End of trial visit (V3)
**Week**	**-20 to -13**	**-9 to -6**	**0**	**10**
**Visit day**	**0**	**1**	**2**	**3**
Informed consent	X			
Review of inclusion and exclusion criteria	X			
Medical history & examination	X			
Registration of medication and adverse events	X	X	X	X
**Anthropometry**				
Body weight	X	X	X	X
Height	X			
Waist circumference	X	X	X	X
Body composition (DXA)			X	X
**Biological Samples**				
Fasting blood sample		X	X	X
Saliva sample (SNPs)		X		
Urine sample		X	X	X
Fecal sample			X*	X*
**Nutritional Assessment**				
Food frequency questionnaire (FFQ)		X		X
3-day dietary records		X*		X*
**Other measurements**				
Blood Pressure/heart rate		X	X	X
Three factor eating questionnaire (TFEQ)			X	X
Perceived stress scale (PSS)			X	X
Behavioral questionnaire (by ONMI)			X	X
Quality of life questionnaires			X	X
Diet satisfaction questionnaire (DSat-28)			X	X
Money spent on food questions			X	X
Accelerometer (sleep and PA)		X**	X**	

Table 4: Procedures and activities during the study period.
X: At home activity prior to visit.*
*X**: At home activity following the visit.*

For peer review only - http://bmjopen.bmj.com/site/about/guidelines.xhtml

Secondary outcomes:

Anthropometry

Body weight will be measured at all visits using a calibrated digital scale to the nearest 0.1 kg with participants wearing lightweight clothes and no shoes, as well as after voiding their bladder. Height will be measured at the screening visit using a wall-mounted stadiometer to the nearest 0.5 cm while participants are not wearing shoes. Body mass index (kg/m^2) will be calculated as weight in kilogram, divided by height in meters squared. Waist circumference (cm) will be measured for all visits in a fasted state (non-fasted in screening) with an empty bladder and after having participants wearing lightweight clothes. It will be measured with a stretch-resistant tape at the midpoint between the lower margin of the last palpable ribs and the top of the iliac crest. Each measurement is taken twice to the nearest 0.5 cm and thereafter an average will be calculated.

Biological Samples

Fasting blood samples will be taken at V1, V2 and V3. Samples will be analyzed for plasma glucose, insulin, cholesterols, triglycerides, adipokines (leptin and adiponectin), inflammatory biomarkers (hs-CRP, IL-6, IL-10, TNFα, MCP1, soluble ICAM1, soluble CD14), lipid profile (total cholesterol, LDL-cholesterol, HDL-cholesterol, non-HDL cholesterol, triglycerides, liver biomarkers (ALT and GGT), renal biomarkers (uric acid, creatinine, eGFR). Blood samples will be sent to Eurecat for metabolomics analysis while biochemical markers will be measured at University of Copenhagen.

Blood pressure

Systolic and diastolic blood pressure and heart rate will be measured using a validated automatic device on the arm after 5-10 min rest in a resting position. The measurement is taken three times (four times if the last two measurements deviate >5 mmHg) with approximately two minutes apart where the reading is recorded to the nearest 1 mmHg. A mean value of the last two readings is used. The same arm will be used for all measurements at all visits.

For peer review only - http://bmjopen.bmj.com/site/about/guidelines.xhtml

Other outcome measures

Saliva

Saliva samples will be collected pre-baseline (V1) and will be sent to Alimentomica (University of the Balearic Islands, Palma de Mallorca) for analysis of genetic variants, mainly single nucleotide polymorphisms (SNPs), in genes related to metabolism, inflammation, and oxidative stress for clustering assignment. Currently, there are 188 candidate SNPs being analyzed and validated in an ongoing trial and a minimum of 35 and maximum of 150 SNPs of those are expected to be used in the present study.

Urine sample

Participants will be asked to deliver a urine spot sample in the morning at the Department at pre-baseline (V1), at baseline (V2), and at the end of the study (V3). Urine samples from V1 and V3 will be sent to UNIPR for food intake biomarkers analysis and to Eurecat for analyzing markers of oxidative stress utilized in the cluster assignment. Furthermore, urine samples from V1, V2 and V3 will be stored in a biobank for future analysis.

Nutritional assessment

Dietary intake will be assessed pre-baseline and at the end of the study using a self-administered electronic Food frequency questionnaire (FFQ) while supervised by trained staff. Furthermore, participants are instructed to complete 3-day weighed dietary records (DRs) twice; one in the week before the pre-baseline visit and another one on the third week of the intervention. The DRs will be recorded on a two non-consecutive weekdays and one weekend day.

Questionnaires

Eating behavioral assessment at baseline and week 10 will be done by administrating the three factor-eating questionnaire (TFEQ).[36] It is a 51-item self-report questionnaire which measures three domains of eating behavior: (1) 'cognitive restraint of eating', (2) 'disinhibition' and (3) 'hunger'.

Stress assessment will be conducted through the perceived stress scale – 10 item (PSS)[37] at baseline and week 10. The PSS is one of the most widely used psychological instruments. It measures the degree to which participants perceive events in their life as being stressful by asking about thoughts and feelings over the last month using a response scale from 0 (never) to 4 (very often).

For peer review only - http://bmjopen.bmj.com/site/about/guidelines.xhtml

Quality of life questionnaires will be collected via the following two instruments at baseline and week 10:

EQ-5D-5L[38]: a standardized instrument developed by EuroQol Group for measuring health-related quality of life on five dimensions (5D); mobility, self-care, usual activities, pain/discomfort, and anxiety/depression, with five response levels per dimension. The instrument also includes a visual analogue scale (EQ-VAS), by which respondents report their perceived health status.

Obesity and Weight-Loss Quality of Life Instrument (OWLQOL)[39]: an instrument consists of 17 statements about weight-related feelings and emotions which are rated on a seven-point scale. OWLQOL primarily measures emotions and feelings resulting from being obese and trying to lose weight.

Diet Satisfaction Questionnaire (DSat-28)[40] involves 28 statements grouped into 5-scales (healthy lifestyle, eating out, cost, preoccupation with food, and planning and preparation) to evaluate individual's satisfaction with weight-management diets. DSat-28 will be completed at baseline and week 10.

Questions about expenditures: two questions will be completed at baseline and week 10 in regard to the amount of money expended on food per household.

Average physical activity level and sleep patterns will be measured by ActiGraph GT3X+ accelerometer (ActiGraph, LLC, Pensacola, FL, USA) for 7 days/8 nights immediately following the pre-baseline visit (V1) and again for 7 days/8 nights in the third week of the intervention. During these wear-periods, a self-administered sleep-log to assess bed times will be obtained.

Microbiota composition

Collection of feces will be performed within 24 hours prior to the clinical investigation days at baseline and week 10. These will not be used in the cluster assignment but will be collected and stored for future analysis. Potential analysis of interest will be to look at baseline composition [i.e. Enterotype] as diet-specific predictor of weight/fat loss and diet-specific change in composition from baseline to week 10.

For peer review only - http://bmjopen.bmj.com/site/about/guidelines.xhtml

Data management

Study investigators will have access to the data collection forms and protocols using a secure shared drive. All collected data will be pseudo-anonymized in which participants will be identified only by a study ID number on documents and on any electronic database, with personal identifiers kept separately under strict access control limited to investigators and study staff directly involved in data collection and entry. All samples of biological material that will be sent to the consortium will be pseudo-anonymized and encrypted. Upon completion of the study, data will be stored in a password-protected database, accessible only by study investigators, in anonymous form for a minimum of 10 years.

Data Monitoring

As the intervention risks to participants are minimal, a Data and Safety Monitoring Board is not needed. However, in case of any unexpected adverse events arise during the study period, they will be recorded and brought to the principal investigator for appropriate decision-making. We do not plan to conduct interim analysis for safety as it is not anticipated that any serious adverse events would require trial discontinuation.

Power and sample size calculation

To detect a 1.25 kg difference in fat mass between the two intervention groups with 80% power with a two-tailed α of 0.05 and assuming a standard deviation (SD) of 2, a sample size of 41 per group (i.e. general dietary recommendations vs. personalized plan) is needed (total N for two groups, 82). Thus, to allow for an 18% drop out rate, 50 subjects per group will needed to be recruited (total N, 100). Expected difference and SD is based on observed values in the SHOPUS study among participants with elevated waist circumference (Men>94.0 cm; Females>80.0 cm) and BMI>27 after 12 weeks of intervention.[41]

Statistical analysis

Data analysis will be conducted using SPSS 27, R or similar statistical software. Before statistical analyses are conducted, all continuous variables will be tested for normality and homogeneity of variance.
The differences in body fat change from baseline to end of trial (week 10) between the two intervention groups (primary objective) will be analyzed by means of linear mixed models including the stratification variables of metabolic clusters as fixed effects, as well as sex, age and BMI at baseline.

For peer review only - http://bmjopen.bmj.com/site/about/guidelines.xhtml

Ethics and dissemination

The study is approved by the Regional Committees on Health Research Ethics, Region Hovedstaden in Denmark (H-20029882) and will be conducted in accordance with the Helsinki Declaration. Any future amendments will be submitted to Research Ethics Board for approval and communicated to study participants and the trial registry once approved.

All personal data is handled confidentially and stored in accordance with applicable law, GDPR and Danish Data Protection Agency. All participants will receive a written and oral information on the study procedures, and only trained study-personnel will provide information, monitor and attest signing of the informed consent form (see online supplemental material 2). In addition, an optional GDPR- consent to provide excess sample materials to study biobank will be signed. The Research biobank has been approved by the Danish Data Protection Agency.

A manuscript with the results of the primary study will be submitted for publication to an international, peer-reviewed journal, regardless of whether the results are positive, negative or inconclusive in relation to the study hypothesis. Authorship eligibility will be based on the recommendations from the International Committee of Medical Journal Editors (ICMJE). Upon completion of the trial, and after publication of the primary manuscript, data requests can be submitted to the principle investigator at the Department of Nutrition, Exercise and sports at University of Copenhagen, Denmark.

Perspectives of the study: The results of this study will serve as proof of concept for the efficacy of using metabolic and genetic biomarkers to deliver personalized nutrition plan for reducing body fat mass and subsequently improve metabolic and inflammatory health. Moreover, the findings will provide recommendations regarding the efficacy of using web-based decision support system for personalizing dietary plans to support and maintain such health-enhancing behaviors.

For peer review only - http://bmjopen.bmj.com/site/about/guidelines.xhtml

Author affiliations

[1]Department of Nutrition, Exercise and Sports, Faculty of Sciences, University of Copenhagen, Denmark.

[2]King Saud bin Abdulaziz University for Health Sciences, Riyadh, Saudi Arabia.

[3]Simple Feast, Copenhagen, Denmark.

[4]Laboratory of Molecular Biology, Nutrition and Biotechnology; NUO group. Universitat de les Illes Balears, Palma, Spain; Institut d'Investigació Sanitària Illes Balears (IdISBa), Palma, Spain; CIBER de Fisiopatología de la Obesidad y Nutrición (CIBEROBN), Madrid, Spain and Alimentómica S.L., Spin-off n.1 of the University of the Balearic Islands, Palma, Spain.

[5]Human Nutrition Unit, Department of Food and Drug, University of Parma, Parma, Italy.

[6]ONMI: Behaviour Change Technology, Eindhoven, Netherland.

[7]Grupo Carinsa, Sant Quirze del Valles, Barcelona, Spain.

[8]Eurecat, Centre Tecnològic de Catalunya, Biotechnology Area, Nutrition and Health Unit, Reus, Spain.

Contributors

The overall framework of the EU-project PREVENTOMICS was initiated by BG, AC and JMDB. The overall design of the present Danish study involved all co-authors. Detailed planning, implementation and daily management of the project was carried out by MAA, KP and MFH. MAA and MFH drafted the initial manuscript. KP, SMOG, PM, FS, MP, MW, JMDB, BG critically reviewed and edited the manuscript. All authors approved the final version for publication.

Trial sponsor: University of Copenhagen

Contact name: Mads Fiil Hjorth

Email: madsfiil@nexs.ku.dk

Address: 1958 Frederiksberg C, Denmark

Funding

This work and the European partners in the PREVENTOMICS project are supported by the European Union's Horizon 2020 research and innovation program under grant agreement No. 818318.

Competing interests None declared.

Patient consent for publication Not required.

Provenance and peer review Not commissioned; externally peer reviewed

Ethics approval

The study is approved by the Scientific Ethics Committee Region H in Denmark with journal number H-20029882, and registered at Clinicaltrials.gov with: NCT04590989.

For peer review only

For peer review only - http://bmjopen.bmj.com/site/about/guidelines.xhtml

Figure 1: study design and timeline. *The analysis results on subjects' metabolome and genotypes will be integrated into PREVENTOMICS platform. This will take around 5 weeks from the date of sending samples from University of Copenhagen to the assigned partners (Eurecat, Alimentomica). SF: Simple feast.

Figure 2: schematic diagram of the intervention. CARB: carbohydrate cluster, LIPID: lipid cluster, INFL: inflammation cluster, OXIS: oxidative stress cluster, MB: microbiota cluster.

For peer review only

For peer review only - http://bmjopen.bmj.com/site/about/guidelines.xhtml

References:

1. Ohlhorst SD, Russell R, Bier D, et al. Nutrition research to affect food and a healthy lifespan. *Adv Nutr* 2013;4(5):579-84.
2. Singhal A. The global epidemic of noncommunicable disease: the role of early-life factors. *Nestle Nutr Inst Workshop Ser* 2014;78:123-32.
3. Hruby A, Hu FB. The Epidemiology of Obesity: A Big Picture. *Pharmacoeconomics* 2015;33(7):673-89.
4. Nyberg ST, Batty GD, Pentti J, et al. Obesity and loss of disease-free years owing to major non-communicable diseases: a multicohort study. *Lancet Public Health* 2018;3(10):e490-e97.
5. Sacks FM, Bray GA, Carey VJ, et al. Comparison of weight-loss diets with different compositions of fat, protein, and carbohydrates. *N Engl J Med* 2009;360(9):859-73.
6. Shai I, Schwarzfuchs D, Henkin Y, et al. Weight loss with a low-carbohydrate, Mediterranean, or low-fat diet. *N Engl J Med* 2008;359(3):229-41.
7. Stanton MV, Robinson JL, Kirkpatrick SM, et al. DIETFITS study (diet intervention examining the factors interacting with treatment success) - Study design and methods. *Contemp Clin Trials* 2017;53:151-61.
8. Hjorth MF, Corella D, Astrup A, et al. High fat diets for weight loss among subjects with elevated fasting glucose levels: The PREDIMED study. *Obesity Medicine* 2020;18:100210.
9. Aleksandrova K, Egea Rodrigues C, Floegel A, et al. Omics Biomarkers in Obesity: Novel Etiological Insights and Targets for Precision Prevention. *Curr Obes Rep* 2020;9(3):219-30.
10. Verma M, Hontecillas R, Tubau-Juni N, et al. Challenges in Personalized Nutrition and Health. *Front Nutr* 2018;5:117.
11. Odriozola L, Corrales FJ. Discovery of nutritional biomarkers: future directions based on omics technologies. *Int J Food Sci Nutr* 2015;66 Suppl 1:S31-40.
12. Chan AW, Tetzlaff JM, Altman DG, et al. SPIRIT 2013 statement: defining standard protocol items for clinical trials. *Ann Intern Med* 2013;158(3):200-7.
13. Salmon J. Excerpts from Dietary Reference Values for Food Energy and Nutrients for the United Kingdom: introduction to the Guide and Summary Tables. *Nutr Rev* 1992;50(3):90-3.
14. Aulchenko YS, Ripatti S, Lindqvist I, et al. Loci influencing lipid levels and coronary heart disease risk in 16 European population cohorts. *Nat Genet* 2009;41(1):47-55.

For peer review only - http://bmjopen.bmj.com/site/about/guidelines.xhtml

15. Dastani Z, Hivert MF, Timpson N, et al. Novel loci for adiponectin levels and their influence on type 2 diabetes and metabolic traits: a multi-ethnic meta-analysis of 45,891 individuals. *PLoS Genet* 2012;8(3):e1002607.

16. Draisma HHM, Pool R, Kobl M, et al. Genome-wide association study identifies novel genetic variants contributing to variation in blood metabolite levels. *Nat Commun* 2015;6:7208.

17. Guan W, Steffen BT, Lemaitre RN, et al. Genome-wide association study of plasma N6 polyunsaturated fatty acids within the cohorts for heart and aging research in genomic epidemiology consortium. *Circ Cardiovasc Genet* 2014;7(3):321-31.

18. Hu Y, Liu W, Huang R, et al. A systematic review and meta-analysis of the relationship between lipoprotein lipase Asn291Ser variant and diseases. *J Lipid Res* 2006;47(9):1908-14.

19. Kathiresan S, Willer CJ, Peloso GM, et al. Common variants at 30 loci contribute to polygenic dyslipidemia. *Nat Genet* 2009;41(1):56-65.

20. Kilpeläinen TO, Carli JF, Skowronski AA, et al. Genome-wide meta-analysis uncovers novel loci influencing circulating leptin levels. *Nat Commun* 2016;7:10494.

21. Tintle NL, Pottala JV, Lacey S, et al. A genome-wide association study of saturated, mono- and polyunsaturated red blood cell fatty acids in the Framingham Heart Offspring Study. *Prostaglandins Leukot Essent Fatty Acids* 2015;94:65-72.

22. Dupuis J, Langenberg C, Prokopenko I, et al. New genetic loci implicated in fasting glucose homeostasis and their impact on type 2 diabetes risk. *Nat Genet* 2010;42(2):105-16.

23. Mahajan A, Sim X, Ng HJ, et al. Identification and functional characterization of G6PC2 coding variants influencing glycemic traits define an effector transcript at the G6PC2-ABCB11 locus. *PLoS Genet* 2015;11(1):e1004876.

24. Manning AK, Hivert MF, Scott RA, et al. A genome-wide approach accounting for body mass index identifies genetic variants influencing fasting glycemic traits and insulin resistance. *Nat Genet* 2012;44(6):659-69.

25. Shin SY, Fauman EB, Petersen AK, et al. An atlas of genetic influences on human blood metabolites. *Nat Genet* 2014;46(6):543-50.

26. Ahola-Olli AV, Würtz P, Havulinna AS, et al. Genome-wide Association Study Identifies 27 Loci Influencing Concentrations of Circulating Cytokines and Growth Factors. *Am J Hum Genet* 2017;100(1):40-50.

27. Ligthart S, Vaez A, Võsa U, et al. Genome Analyses of >200,000 Individuals Identify 58 Loci for Chronic Inflammation and Highlight Pathways that Link Inflammation and Complex Disorders. *Am J Hum Genet* 2018;103(5):691-706.

For peer review only - http://bmjopen.bmj.com/site/about/guidelines.xhtml

28. Ljungman P, Bellander T, Nyberg F, et al. DNA variants, plasma levels and variability of interleukin-6 in myocardial infarction survivors: results from the AIRGENE study. *Thromb Res* 2009;124(1):57-64.

29. Qi L, van Dam RM, Meigs JB, et al. Genetic variation in IL6 gene and type 2 diabetes: tagging-SNP haplotype analysis in large-scale case-control study and meta-analysis. *Hum Mol Genet* 2006;15(11):1914-20.

30. Galmés S, Cifre M, Palou A, et al. A Genetic Score of Predisposition to Low-Grade Inflammation Associated with Obesity May Contribute to Discern Population at Risk for Metabolic Syndrome. *Nutrients* 2019;11(2)

31. Jerotic D, Matic M, Suvakov S, et al. Association of Nrf2, SOD2 and GPX1 Polymorphisms with Biomarkers of Oxidative Distress and Survival in End-Stage Renal Disease Patients. *Toxins (Basel)* 2019;11(7)

32. van Meurs JB, Pare G, Schwartz SM, et al. Common genetic loci influencing plasma homocysteine concentrations and their effect on risk of coronary artery disease. *Am J Clin Nutr* 2013;98(3):668-76.

33. Jia Q, Han Y, Huang P, et al. Genetic Determinants of Circulating Glycine Levels and Risk of Coronary Artery Disease. *J Am Heart Assoc* 2019;8(10):e011922.

34. Wittemans LBL, Lotta LA, Oliver-Williams C, et al. Assessing the causal association of glycine with risk of cardio-metabolic diseases. *Nat Commun* 2019;10(1):1060.

35. Habibović M, Broers E, Piera-Jimenez J, et al. Enhancing Lifestyle Change in Cardiac Patients Through the Do CHANGE System ("Do Cardiac Health: Advanced New Generation Ecosystem"): Randomized Controlled Trial Protocol. *JMIR Res Protoc* 2018;7(2):e40.

36. Stunkard AJ, Messick S. The three-factor eating questionnaire to measure dietary restraint, disinhibition and hunger. *J Psychosom Res* 1985;29(1):71-83.

37. Cohen S, Kamarck T, Mermelstein R. A global measure of perceived stress. *J Health Soc Behav* 1983;24(4):385-96.

38. EuroQol--a new facility for the measurement of health-related quality of life. *Health Policy* 1990;16(3):199-208.

39. Niero M, Martin M, Finger T, et al. A new approach to multicultural item generation in the development of two obesity-specific measures: the Obesity and Weight Loss Quality of Life (OWLQOL) questionnaire and the Weight-Related Symptom Measure (WRSM). *Clin Ther* 2002;24(4):690-700.

40. James BL, Loken E, Roe LS, et al. Validation of the Diet Satisfaction Questionnaire: a new measure of satisfaction with diets for weight management. *Obes Sci Pract* 2018;4(6):506-14.

For peer review only - http://bmjopen.bmj.com/site/about/guidelines.xhtml

41. Poulsen SK, Due A, Jordy AB, et al. Health effect of the New Nordic Diet in adults with increased waist circumference: a 6-mo randomized controlled trial. *Am J Clin Nutr* 2014;99(1):35-45.

For peer review only

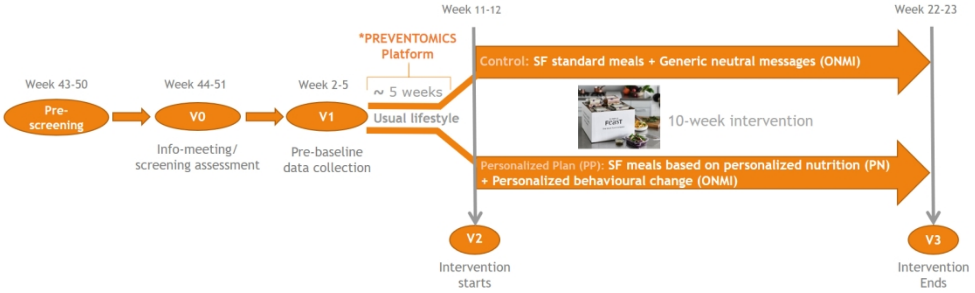

Figure 1: study design and timeline.*The analysis results on subjects' metabolome and genotypes will be integrated into PREVENTOMICS platform. This will take around 5 weeks from the date of sending samples from University of Copenhagen to the assigned partners (Eurecat, Alimentomica). SF: Simple feast.

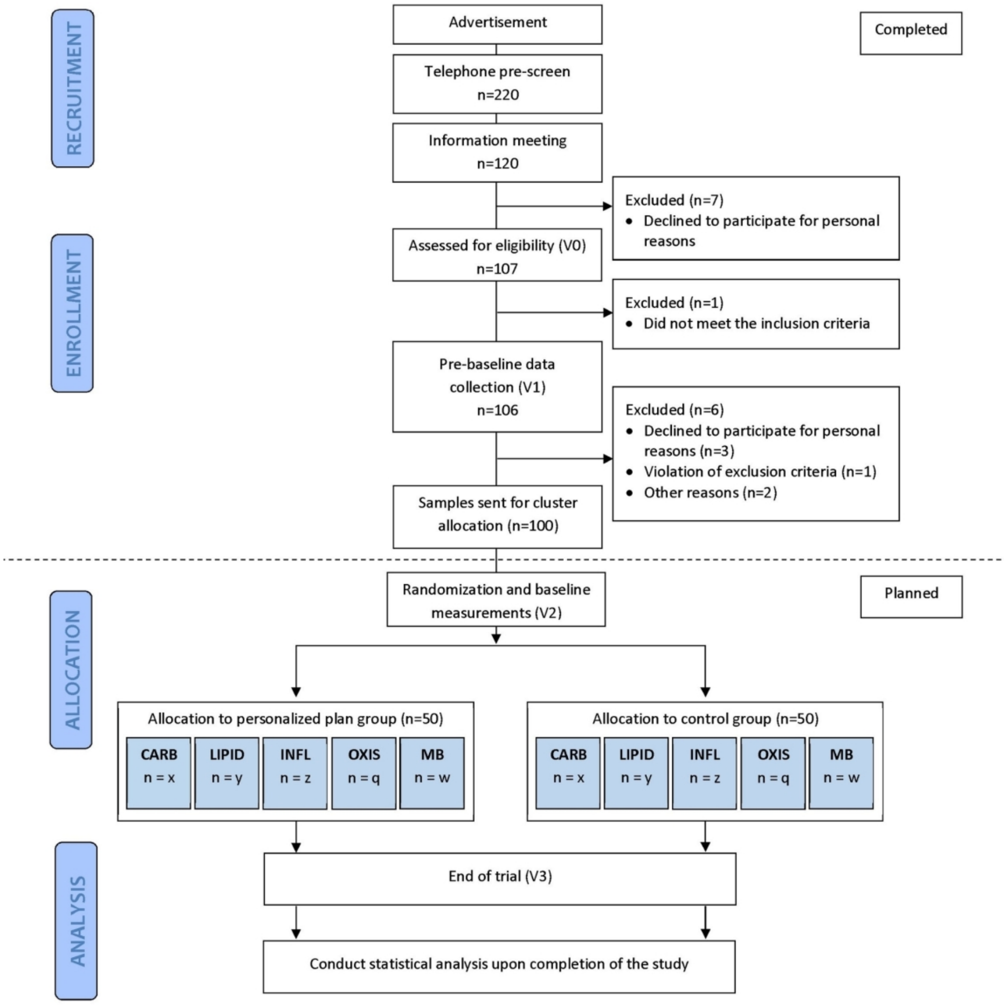

Figure 2: schematic diagram of the intervention. CARB: carbohydrate cluster, LIPID: lipid cluster, INFL: inflammation cluster, OXIS: oxidative stress cluster, MB: microbiota cluster.

170x175mm (300 x 300 DPI)

SUPPLEMENTARY ONLINE MATERIAL 1: References for biomarkers included in each cluster

(1) Carbohydrates cluster		
**Biomarker**	**Bio-fluid**	**Reference**
**Glucose**	Plasma	Cosentino et al., 2020[1]
**HOMA-IR**	-	Bloomgarden, 2003[2]<br>Govers et al., 2015[3]<br>Shashaj et al., 2016[4]
**Insulin**	Serum	Aleksandrova et al., 2018[5]<br>Bloomgarden, 2003[2]<br>Govers et al., 2015[3]
**Leptin**	Serum	Chen et al., 2014[6]<br>Finucane et al., 2019[7]<br>López-Jaramillo et al., 2014[8]
**Adiponectin**	Serum	Dastani et al., 2012[9]<br>Li et al., 2009[10]<br>Liu et al., 2016[11]<br>Wang et al., 2018[12]
**Lactate**	Serum	Berhane et al., 2015[13]<br>Choi et al., 2002[14]<br>Lovejoy et al., 1992[15]<br>Shantha et al., 2013[16]
**Glutamate**	Serum	Martin and Price, 2018[17]<br>Ottosson et al., 2018[18]
**Uric acid**	Serum	Darmawan et al., 2018[19]<br>Fabbrini et al., 2014[20]<br>Johnson et al., 2013[21]<br>van der Schaft et al., 2017[22]
**Propionylcarnitine**	Plasma	Mai et al., 2013[23]<br>Mihalik et al., 2010[24]<br>Zhang et al., 2014[25]
**BCAA (Valine, Leucine, Isoleucine)**	Serum	Chen et al., 2016[26]<br>Katagiri et al., 2018[27]

For peer review only

		Lotta et al., 2016[28]
		Newgard et al., 2009[29]
		Okekunle et al., 2019[30]
**Phenylalanine**	Serum	Chen et al., 2019[31]
		Suzuki et al., 2019[32]
		Wang et al., 2011[33]
**Tyrosine**	Serum	Chen et al., 2019[31]
		Hellmuth et al., 2016[34]
		Wang et al., 2011[33]
**Glutamine**	Serum	Chen et al., 2019[31]
		Guasch-Ferré et al., 2016[35]
		Liu et al., 2019[36]
		Rhee et al., 2018[37]

Table 1: *biomarkers included in carbohydrate cluster. BCAA: branched chain amino acids.*

For peer review only

(2) Lipid cluster		
**Biomarker**	**Bio-fluid**	**Reference**
**LDL-cholesterol**	Plasma	Grundy et al., 2019[38]
**HDL-cholesterol**	Plasma	Grundy et al., 2019[38]
**Triglycerides**	Plasma	Grundy et al., 2019[38]
**Total cholesterol**	Plasma	Grundy et al., 2019[38]
**PUFAs (total)**	Serum	Koga et al., 2019[39]
**LPCs (total)**	Plasma	Law et al., 2019[40]
**Linoleic acid C18:2**	Serum	Koga et al., 2019[39]
**Oleic acid C18:1**	Serum	Griel and Kris-Etherton, 2006[41]<br>Yamagishi et al., 2013[42]
**Leptin**	Serum	Mantzoros and Flier, 2000[43]
**Adiponectin**	Serum	Abdella and Mojiminiyi, 2018[44]<br>Liu et al., 2018[45]
**Saturated fatty acids (SFAs)**	Serum	Liu et al., 2019[46]
**3-hydroxybutyrate**	Serum	Margolis and O'Fallon, 2020[47]
**MUFAs (total)**	Serum	Griel and Kris-Etherton, 2006[41]<br>Yamagishi et al., 2013[42]
**Propionylcarnitine**	Plasma	Ottosson et al., 2018[18]
**DHA C20:3**	Serum	Koga et al., 2019[39]
**Choline**	Serum	Ottosson et al., 2018[18]

Table 2: *biomarkers included in lipid cluster. LDL: low-density lipoprotein, HDL: high-density lipoprotein, PUFAs: poly unsaturated fatty acids. LPC: lysophosphatidylcholines, SFAs: saturated fatty acids, MUFAs: monounsaturated fatty acids. DHA: docosahexaenoic acid.*

For peer review only

(3) Inflammation cluster		
**Biomarker**	**Bio-fluid**	**Reference**
**CRP**	Serum	Amsterdam, 2003[48]
		Knight, 2015[49]
		Liu et al., 2016[11]
		Musunuru et al., 2008[50]
		Wang et el., 2013[51]
**IL-6**	Plasma	Liu et al., 2016[11]
		Wang et al., 2013[51]
**N-acetylglycoproteins**	Serum	Gruppen et al., 2019[52]
		Ritchie et al., 2015[53]
**TNFα**	Plasma	Liu et al., 2016[11]
**MCP-1**	Plasma	Georgakis et al., 2019[54]
**IL-10**	Plasma	Charles et al., 2011[55]
		Leon-Cabrera et al., 2015[56]
		Meng et al., 2019[57]
**sICAM1**	Plasma	El Amine et al., 2010[58]
		Luc et al., 2003[59]
		Straczkowski et al., 2002[60]
**LBP**	Plasma	Moreno-Navarrete et al., 2012[61]
**sCD14**	Plasma	de Courten et al., 2016[62]
**LPCs (total)**	Plasma	Iwase et al., 2008[63]
**Linoleic acid C18:2**	Serum	Steffen et al., 2012[64]
		Yli-Jama et al., 2002[65]
**DHA C20:3**	Serum	Steffen et al., 2012[64]
		Yli-Jama et al., 2002[65]

Table 3: *biomarkers included in inflammation cluster. CRP: C-reactive protein, IL-6: interleukin-6, IL-10: interleukin-10, TNFα: tumor necrosis factor alpha, MCP-1: monocyte chemoattractant protein-1, sICAM-1: soluble intercellular adhesion molecule-1. LBP: lipopolysaccharide binding protein, sCD14: soluble CD14, LPC: lysophosphatidylcholines, DHA: docosahexaenoic acid.*

For peer review only

(4) Oxidative stress cluster		
**Biomarker**	**Bio-fluid**	**Reference**
**8-iso-PGF2α**	Urine	Davies and Roberts, 2011[66]
		Kim et al., 2012[67]
		Milne et al., 2015[68]
		van't Erve, 2018[69]
**8-OHdG**	Urine	Di Minno et al., 2016[70]
		Kroese and Scheffe, 2014[71]
**LDLox**	Plasma	Barbosa et al., 2011[72]
		Gao et al., 2017[73]
**Uric acid**	Serum	Darmawan et al., 2018[19]
		Fabbrini et al., 2014[20]
		van der Schaft et al., 2017[22]
**Allantoin**	Urine	Il'yasova et al., 2012[74]
**Betaine**	Urine	Svingen et al., 2016[75]
		Walford et al., 2016[76]
**Pseudouridine**	Urine	Topp et al., 2008[77]
**Dimethylglycine**	Urine	Svingen et al., 2016[75]
**Glycine**	Serum	Sekhar et al., 2011[78]
**Methionine**	Serum	Grizales et al., 2018[79]

Table 4: *biomarkers included in oxidative stress cluster. 8-iso-PGF2α: 8-iso-prostaglandin F2 alpha, 8-OHdG: 8-hydroxy 2-deoxyguanosine, LDLox: oxidized low density lipoprotein.*

For peer review only

(5) Microbiota cluster		
**Biomarker**	**Bio-fluid**	**Reference**
**Trimethylamine N-oxide (TMAO)**	Serum	Bain et al., 2006[80]<br>Chen et al., 2016[81]<br>Ge et al., 2020[82]<br>Heianza et al., 2017[83]<br>Schiattarella et al., 2017[84]<br>Yang et el., 2019[85]<br>Yao et al., 2020[86]
**Trimethylamine (TMA)**	Urine	Aragonès et al., 2019[87]<br>Bouatra et al., 2013[88]<br>Chen et al., 2016[81]<br>Palau-Rodriguez et al., 2015[89]<br>Yang et el., 2019[85]
**Dimethylamine (DMA)**	Urine	Bouatra et al., 2013[88]<br>Palau-Rodriguez et al., 2015[89]
**Betaine**	Urine	Chen et al., 2016[81]<br>Palau-Rodriguez et al., 2015[89]<br>Yang et el., 2019[85]
**Choline**	Serum	Aragonès et al., 2019[87]<br>Chen et al., 2016[81]<br>Palau-Rodriguez et al., 2015[89]<br>Yang et el., 2019[85]
**Dimethylglycine**	Urine	Palau-Rodriguez et al., 2015[89]
**Succinate**	Plasma	Psychogios et al., 2011[90]<br>Serena et al., 2018[91]
**Lactate**	Serum	Psychogios et al., 2011[90]<br>Vojinovic et al., 2019[92]
**LBP**	Plasma	Awoyemi et al., 2018[93]<br>Barengolts et al., 2019[94]<br>Bouatra et al., 2013[88]<br>Citronberg et al., 2018[95]
**Acetate**	Urine	Liu et al., 2014[96]

Table 5: biomarkers included in microbiota cluster. LBP: lipopolysaccharide binding protein.

For peer review only

References:

1. Cosentino F, Grant PJ, Aboyans V, et al. 2019 ESC Guidelines on diabetes, pre-diabetes, and cardiovascular diseases developed in collaboration with the EASD. *Eur Heart J* 2020;41(2):255-323.
2. Bloomgarden ZT. American Association of Clinical Endocrinologists (AACE) consensus conference on the insulin resistance syndrome: 25-26 August 2002, Washington, DC. *Diabetes Care* 2003;26(4):1297-303.
3. Govers E, Slof E, Verkoelen H, et al. Guideline for the Management of Insulin Resistance. *Int J Endocrinol Metab Disord* 2015;1(4).
4. Shashaj B, Luciano R, Contoli B, et al. Reference ranges of HOMA-IR in normal-weight and obese young Caucasians. *Acta Diabetol* 2016;53(2):251-60.
5. Aleksandrova K, Mozaffarian D, Pischon T. Addressing the Perfect Storm: Biomarkers in Obesity and Pathophysiology of Cardiometabolic Risk. *Clin Chem* 2018;64(1):142-53.
6. Chen GC, Qin LQ, Ye JK. Leptin levels and risk of type 2 diabetes: gender-specific meta-analysis. *Obes Rev* 2014;15(2):134-42.
7. Finucane FM, Luan J, Wareham NJ, et al. Correlation of the leptin:adiponectin ratio with measures of insulin resistance in non-diabetic individuals. *Diabetologia* 2009;52(11):2345-49.
8. López-Jaramillo P, Gómez-Arbeláez D, López-López J, et al. The role of leptin/adiponectin ratio in metabolic syndrome and diabetes. *Horm Mol Biol Clin Investig* 2014;18(1):37-45.
9. Dastani Z, Hivert MF, Timpson N, et al. Novel loci for adiponectin levels and their influence on type 2 diabetes and metabolic traits: a multi-ethnic meta-analysis of 45,891 individuals. *PLoS Genet* 2012;8(3):e1002607.
10. Li S, Shin HJ, Ding EL, et al. Adiponectin levels and risk of type 2 diabetes: a systematic review and meta-analysis. *Jama* 2009;302(2):179-88.
11. Liu C, Feng X, Li Q, et al. Adiponectin, TNF-α and inflammatory cytokines and risk of type 2 diabetes: A systematic review and meta-analysis. *Cytokine* 2016;86:100-09.
12. Wang Y, Meng RW, Kunutsor SK, et al. Plasma adiponectin levels and type 2 diabetes risk: a nested case-control study in a Chinese population and an updated meta-analysis. *Sci Rep* 2018;8(1):406.
13. Berhane F, Fite A, Daboul N, et al. Plasma Lactate Levels Increase during Hyperinsulinemic Euglycemic Clamp and Oral Glucose Tolerance Test. *J Diabetes Res* 2015;2015:102054.
14. Choi CS, Kim YB, Lee FN, et al. Lactate induces insulin resistance in skeletal muscle by suppressing glycolysis and impairing insulin signaling. *Am J Physiol Endocrinol Metab* 2002;283(2):E233-40.
15. Lovejoy J, Newby FD, Gebhart SSP, et al. Insulin resistance in obesity is associated with elevated basal lactate levels and diminished lactate appearance following intravenous glucose and insulin. *Metabolism* 1992;41(1):22-27.

For peer review only

16. Shantha GP, Wasserman B, Astor BC, et al. Association of blood lactate with carotid atherosclerosis: the Atherosclerosis Risk in Communities (ARIC) Carotid MRI Study. *Atherosclerosis* 2013;228(1):249-55.

17. Martin AJ, Price CI. A Systematic Review and Meta-Analysis of Molecular Biomarkers Associated with Early Neurological Deterioration Following Acute Stroke. *Cerebrovasc Dis* 2018;46(5-6):230-41.

18. Ottosson F, Brunkwall L, Ericson U, et al. Connection Between BMI-Related Plasma Metabolite Profile and Gut Microbiota. *J Clin Endocrinol Metab* 2018;103(4):1491-501.

19. Darmawan G, Hamijoyo L, Hasan I. Association between Serum Uric Acid and Non-Alcoholic Fatty Liver Disease: A Meta-Analysis. *Acta Med Indones* 2017;49(2):136-47.

20. Fabbrini E, Serafini M, Colic Baric I, et al. Effect of plasma uric acid on antioxidant capacity, oxidative stress, and insulin sensitivity in obese subjects. *Diabetes* 2014;63(3):976-81.

21. Johnson RJ, Nakagawa T, Sanchez-Lozada LG, et al. Sugar, uric acid, and the etiology of diabetes and obesity. *Diabetes* 2013;62(10):3307-15.

22. van der Schaft N, Brahimaj A, Wen KX, et al. The association between serum uric acid and the incidence of prediabetes and type 2 diabetes mellitus: The Rotterdam Study. *PLoS One* 2017;12(6):e0179482.

23. Mai M, Tönjes A, Kovacs P, et al. Serum levels of acylcarnitines are altered in prediabetic conditions. *PLoS One* 2013;8(12):e82459.

24. Mihalik SJ, Goodpaster BH, Kelley DE, et al. Increased levels of plasma acylcarnitines in obesity and type 2 diabetes and identification of a marker of glucolipotoxicity. *Obesity (Silver Spring)* 2010;18(9):1695-700.

25. Zhang X, Zhang C, Chen L, et al. Human serum acylcarnitine profiles in different glucose tolerance states. *Diabetes Res Clin Pract* 2014;104(3):376-82.

26. Chen T, Ni Y, Ma X, et al. Branched-chain and aromatic amino acid profiles and diabetes risk in Chinese populations. *Sci Rep* 2016;6:20594.

27. Katagiri R, Goto A, Budhathoki S, et al. Association between plasma concentrations of branched-chain amino acids and adipokines in Japanese adults without diabetes. *Sci Rep* 2018;8(1):1043.

28. Lotta LA, Scott RA, Sharp SJ, et al. Genetic Predisposition to an Impaired Metabolism of the Branched-Chain Amino Acids and Risk of Type 2 Diabetes: A Mendelian Randomisation Analysis. *PLoS Med* 2016;13(11):e1002179.

29. Newgard CB, An J, Bain JR, et al. A branched-chain amino acid-related metabolic signature that differentiates obese and lean humans and contributes to insulin resistance. *Cell Metab* 2009;9(4):311-26.

For peer review only

30. Okekunle AP, Zhang M, Wang Z, et al. Dietary branched-chain amino acids intake exhibited a different relationship with type 2 diabetes and obesity risk: a meta-analysis. *Acta Diabetol* 2019;56(2):187-95.
31. Chen S, Akter S, Kuwahara K, et al. Serum amino acid profiles and risk of type 2 diabetes among Japanese adults in the Hitachi Health Study. *Sci Rep* 2019;9(1):7010.
32. Suzuki Y, Kido J, Matsumoto S, et al. Associations among amino acid, lipid, and glucose metabolic profiles in childhood obesity. *BMC Pediatr* 2019;19(1):273.
33. Wang TJ, Larson MG, Vasan RS, et al. Metabolite profiles and the risk of developing diabetes. *Nat Med* 2011;17(4):448-53.
34. Hellmuth C, Kirchberg FF, Lass N, et al. Tyrosine Is Associated with Insulin Resistance in Longitudinal Metabolomic Profiling of Obese Children. *J Diabetes Res* 2016;2016:2108909.
35. Guasch-Ferré M, Hruby A, Toledo E, et al. Metabolomics in Prediabetes and Diabetes: A Systematic Review and Meta-analysis. *Diabetes Care* 2016;39(5):833-46.
36. Liu X, Zheng Y, Guasch-Ferré M, et al. High plasma glutamate and low glutamine-to-glutamate ratio are associated with type 2 diabetes: Case-cohort study within the PREDIMED trial. *Nutr Metab Cardiovasc Dis* 2019;29(10):1040-49.
37. Rhee SY, Jung ES, Park HM, et al. Plasma glutamine and glutamic acid are potential biomarkers for predicting diabetic retinopathy. *Metabolomics* 2018;14(7):89.
38. Grundy SM, Stone NJ, Bailey AL, et al. 2018 AHA/ACC/AACVPR/AAPA/ABC/ACPM/ADA/AGS/APhA/ASPC/NLA/PCNA Guideline on the Management of Blood Cholesterol: A Report of the American College of Cardiology/American Heart Association Task Force on Clinical Practice Guidelines. *J Am Coll Cardiol* 2019;73(24):e285-e350.
39. Koga N, Ogura J, Yoshida F, et al. Altered polyunsaturated fatty acid levels in relation to proinflammatory cytokines, fatty acid desaturase genotype, and diet in bipolar disorder. *Transl Psychiatry* 2019;9(1):208.
40. Law SH, Chan ML, Marathe GK, et al. An Updated Review of Lysophosphatidylcholine Metabolism in Human Diseases. *Int J Mol Sci* 2019;20(5).
41. Griel AE, Kris-Etherton PM. Tree nuts and the lipid profile: a review of clinical studies. *Br J Nutr* 2006;96 Suppl 2:S68-78.
42. Yamagishi K, Folsom AR, Steffen LM. Plasma fatty acid composition and incident ischemic stroke in middle-aged adults: the Atherosclerosis Risk in Communities (ARIC) Study. *Cerebrovasc Dis* 2013;36(1):38-46.
43. Mantzoros CS, Flier JS. Editorial: leptin as a therapeutic agent--trials and tribulations. *J Clin Endocrinol Metab* 2000;85(11):4000-2.

For peer review only

44. Abdella NA, Mojiminiyi OA. Clinical Applications of Adiponectin Measurements in Type 2 Diabetes Mellitus: Screening, Diagnosis, and Marker of Diabetes Control. *Dis Markers* 2018;2018:5187940.

45. Liu Z, Liang S, Que S, et al. Meta-Analysis of Adiponectin as a Biomarker for the Detection of Metabolic Syndrome. *Front Physiol* 2018;9:1238.

46. Liu Q, Matthan NR, Manson JE, et al. Plasma Phospholipid Fatty Acids and Coronary Heart Disease Risk: A Matched Case-Control Study within the Women's Health Initiative Observational Study. *Nutrients* 2019;11(7).

47. Margolis LM, O'Fallon KS. Utility of Ketone Supplementation to Enhance Physical Performance: A Systematic Review. *Adv Nutr* 2020;11(2):412-19.

48. Amsterdam EA. C-reactive protein: a guideline for its application. *Prev Cardiol* 2003;6(2):70.

49. Knight ML. The Application of High-Sensitivity C-Reactive Protein in Clinical Practice: A 2015 Update. *US Pharm* 2015;40(2):50-53.

50. Musunuru K, Kral BG, Blumenthal RS, et al. The use of high-sensitivity assays for C-reactive protein in clinical practice. *Nat Clin Pract Cardiovasc Med* 2008;5(10):621-35.

51. Wang X, Bao W, Liu J, et al. Inflammatory markers and risk of type 2 diabetes: a systematic review and meta-analysis. *Diabetes Care* 2013;36(1):166-75.

52. Gruppen EG, Kunutsor SK, Kieneker LM, et al. GlycA, a novel pro-inflammatory glycoprotein biomarker is associated with mortality: results from the PREVEND study and meta-analysis. *J Intern Med* 2019;286(5):596-609.

53. Ritchie SC, Würtz P, Nath AP, et al. The Biomarker GlycA Is Associated with Chronic Inflammation and Predicts Long-Term Risk of Severe Infection. *Cell Syst* 2015;1(4):293-301.

54. Georgakis MK, Malik R, Björkbacka H, et al. Circulating Monocyte Chemoattractant Protein-1 and Risk of Stroke: Meta-Analysis of Population-Based Studies Involving 17 180 Individuals. *Circ Res* 2019;125(8):773-82.

55. Charles BA, Doumatey A, Huang H, et al. The roles of IL-6, IL-10, and IL-1RA in obesity and insulin resistance in African-Americans. *J Clin Endocrinol Metab* 2011;96(12):E2018-22.

56. Leon-Cabrera S, Arana-Lechuga Y, Esqueda-León E, et al. Reduced systemic levels of IL-10 are associated with the severity of obstructive sleep apnea and insulin resistance in morbidly obese humans. *Mediators Inflamm* 2015;2015:493409.

57. Meng D, Liang L, Guo X. Serum interleukin-10 level in patients with inflammatory bowel disease: A meta-analysis. *Eur J Inflamm* 2019;17:1-7.

58. El Amine M, Sohawon S, Lagneau L, et al. Plasma levels of ICAM-1 and circulating endothelial cells are elevated in unstable types 1 and 2 diabetes. *Endocr Regul* 2010;44(1):17-24.

59. Luc G, Arveiler D, Evans A, et al. Circulating soluble adhesion molecules ICAM-1 and VCAM-1 and incident coronary heart disease: the PRIME Study. *Atherosclerosis* 2003;170(1):169-76.

For peer review only

60. Straczkowski M, Lewczuk P, Dzienis-Straczkowska S, et al. Elevated soluble intercellular adhesion molecule-1 levels in obesity: relationship to insulin resistance and tumor necrosis factor-alpha system activity. *Metabolism* 2002;51(1):75-8.

61. Moreno-Navarrete JM, Ortega F, Serino M, et al. Circulating lipopolysaccharide-binding protein (LBP) as a marker of obesity-related insulin resistance. *Int J Obes (Lond)* 2012;36(11):1442-9.

62. de Courten B, Moreno-Navarrete JM, Lyons J, et al. Contrasting association of circulating sCD14 with insulin sensitivity in non-obese and morbidly obese subjects. *Mol Nutr Food Res* 2016;60(1):103-9.

63. Iwase M, Sonoki K, Sasaki N, et al. Lysophosphatidylcholine contents in plasma LDL in patients with type 2 diabetes mellitus: relation with lipoprotein-associated phospholipase A2 and effects of simvastatin treatment. *Atherosclerosis* 2008;196(2):931-6.

64. Steffen BT, Steffen LM, Tracy R, et al. Obesity modifies the association between plasma phospholipid polyunsaturated fatty acids and markers of inflammation: the Multi-Ethnic Study of Atherosclerosis. *Int J Obes (Lond)* 2012;36(6):797-804.

65. Yli-Jama P, Seljeflot I, Meyer HE, et al. Serum non-esterified very long-chain PUFA are associated with markers of endothelial dysfunction. *Atherosclerosis* 2002;164(2):275-81.

66. Davies SS, Roberts LJ, 2nd. F2-isoprostanes as an indicator and risk factor for coronary heart disease. *Free Radic Biol Med* 2011;50(5):559-66.

67. Kim JY, Lee JW, Youn YJ, et al. Urinary levels of 8-iso-prostaglandin f2α and 8-hydroxydeoxyguanine as markers of oxidative stress in patients with coronary artery disease. *Korean Circ J* 2012;42(9):614-7.

68. Milne GL, Dai Q, Roberts LJ, 2nd. The isoprostanes--25 years later. *Biochim Biophys Acta* 2015;1851(4):433-45.

69. van 't Erve TJ. Strategies to decrease oxidative stress biomarker levels in human medical conditions: A meta-analysis on 8-iso-prostaglandin F(2α). *Redox Biol* 2018;17:284-96.

70. Di Minno A, Turnu L, Porro B, et al. 8-Hydroxy-2-Deoxyguanosine Levels and Cardiovascular Disease: A Systematic Review and Meta-Analysis of the Literature. *Antioxid Redox Signal* 2016;24(10):548-55.

71. Kroese LJ, Scheffer PG. 8-hydroxy-2'-deoxyguanosine and cardiovascular disease: a systematic review. *Curr Atheroscler Rep* 2014;16(11):452.

72. Barbosa KB, Volp AC, Hermsdorff HH, et al. Relationship of oxidized low density lipoprotein with lipid profile and oxidative stress markers in healthy young adults: a translational study. *Lipids Health Dis* 2011;10:61.

73. Gao S, Zhao D, Wang M, et al. Association Between Circulating Oxidized LDL and Atherosclerotic Cardiovascular Disease: A Meta-analysis of Observational Studies. *Can J Cardiol* 2017;33(12):1624-32.

For peer review only

74. Il'yasova D, Scarbrough P, Spasojevic I. Urinary biomarkers of oxidative status. *Clin Chim Acta* 2012;413(19-20):1446-53.

75. Svingen GF, Schartum-Hansen H, Pedersen ER, et al. Prospective Associations of Systemic and Urinary Choline Metabolites with Incident Type 2 Diabetes. *Clin Chem* 2016;62(5):755-65.

76. Walford GA, Ma Y, Clish C, et al. Metabolite Profiles of Diabetes Incidence and Intervention Response in the Diabetes Prevention Program. *Diabetes* 2016;65(5):1424-33.

77. Topp H, Fusch G, Schöch G, et al. Noninvasive markers of oxidative DNA stress, RNA degradation and protein degradation are differentially correlated with resting metabolic rate and energy intake in children and adolescents. *Pediatr Res* 2008;64(3):246-50.

78. Sekhar RV, McKay SV, Patel SG, et al. Glutathione synthesis is diminished in patients with uncontrolled diabetes and restored by dietary supplementation with cysteine and glycine. *Diabetes Care* 2011;34(1):162-7.

79. Grizales AM, Patti ME, Lin AP, et al. Metabolic Effects of Betaine: A Randomized Clinical Trial of Betaine Supplementation in Prediabetes. *J Clin Endocrinol Metab* 2018;103(8):3038-49.

80. Bain MA, Faull R, Fornasini G, et al. Accumulation of trimethylamine and trimethylamine-N-oxide in end-stage renal disease patients undergoing haemodialysis. *Nephrol Dial Transplant* 2006;21(5):1300-4.

81. Chen YM, Liu Y, Zhou RF, et al. Associations of gut-flora-dependent metabolite trimethylamine-N-oxide, betaine and choline with non-alcoholic fatty liver disease in adults. *Sci Rep* 2016;6:19076.

82. Ge X, Zheng L, Zhuang R, et al. The Gut Microbial Metabolite Trimethylamine N-Oxide and Hypertension Risk: A Systematic Review and Dose-Response Meta-analysis. *Adv Nutr* 2020;11(1):66-76.

83. Heianza Y, Ma W, Manson JE, et al. Gut Microbiota Metabolites and Risk of Major Adverse Cardiovascular Disease Events and Death: A Systematic Review and Meta-Analysis of Prospective Studies. *J Am Heart Assoc* 2017;6(7)

84. Schiattarella GG, Sannino A, Toscano E, et al. Gut microbe-generated metabolite trimethylamine-N-oxide as cardiovascular risk biomarker: a systematic review and dose-response meta-analysis. *Eur Heart J* 2017;38(39):2948-56.

85. Yang S, Li X, Yang F, et al. Gut Microbiota-Dependent Marker TMAO in Promoting Cardiovascular Disease: Inflammation Mechanism, Clinical Prognostic, and Potential as a Therapeutic Target. *Front Pharmacol* 2019;10:1360.

86. Yao ME, Liao PD, Zhao XJ, et al. Trimethylamine-N-oxide has prognostic value in coronary heart disease: a meta-analysis and dose-response analysis. *BMC Cardiovasc Disord* 2020;20(1):7.

87. Aragonès G, González-García S, Aguilar C, et al. Gut Microbiota-Derived Mediators as Potential Markers in Nonalcoholic Fatty Liver Disease. *Biomed Res Int* 2019;2019:8507583.

For peer review only

88. Bouatra S, Aziat F, Mandal R, et al. The human urine metabolome. *PLoS One* 2013;8(9):e73076.

89. Palau-Rodriguez M, Tulipani S, Isabel Queipo-Ortuño M, et al. Metabolomic insights into the intricate gut microbial-host interaction in the development of obesity and type 2 diabetes. *Front Microbiol* 2015;6:1151.

90. Psychogios N, Hau DD, Peng J, et al. The human serum metabolome. *PLoS One* 2011;6(2):e16957.

91. Serena C, Ceperuelo-Mallafré V, Keiran N, et al. Elevated circulating levels of succinate in human obesity are linked to specific gut microbiota. *Isme j* 2018;12(7):1642-57.

92. Vojinovic D, Radjabzadeh D, Kurilshikov A, et al. Relationship between gut microbiota and circulating metabolites in population-based cohorts. *Nat Commun* 2019;10(1):5813.

93. Awoyemi A, Trøseid M, Arnesen H, et al. Markers of metabolic endotoxemia as related to metabolic syndrome in an elderly male population at high cardiovascular risk: a cross-sectional study. *Diabetol Metab Syndr* 2018;10:59.

94. Barengolts E, Green SJ, Chlipala GE, et al. Predictors of Obesity among Gut Microbiota Biomarkers in African American Men with and without Diabetes. *Microorganisms* 2019;7(9).

95. Citronberg JS, Curtis KR, White E, et al. Association of gut microbial communities with plasma lipopolysaccharide-binding protein (LBP) in premenopausal women. *Isme j* 2018;12(7):1631-41.

96. Liu X, Lu L, Yao P, et al. Lipopolysaccharide binding protein, obesity status and incidence of metabolic syndrome: a prospective study among middle-aged and older Chinese. *Diabetologia* 2014;57(9):1834-41.

For peer review only

SUPPLEMENTARY ONLINE MATERIAL 2: informed consent version 2.0, 18 September 2020 related to PREVENTOMICS Participant Information version 3.0.

Title of research project: **PREVENTOMICS: Empowering consumers to PREVENT diet-related diseases through OMICS-based personalized nutrition**

Consent from the study participant:

I have obtained written and oral information about the research project and I am informed about the aim, methods, benefits and risks of participating in the study.

I know that it is completely voluntary to participate, and I have the right to withdraw the informed consent at any time and with no loss of benefits to which I am otherwise entitled.

I consent to participate in the research project and that my biological material will be stored in a research biobank. I have received a copy of this informed consent form as well as a copy of the written information.

Study participant name: ____

Date: _______________ Signature: ____

In case new information that has substantial influence on your health emerges from the research project, you will be informed. Would you prefer **not** to be informed about information that has substantial influence on your health, please mark it here _________ (insert X).

Do you wish to be informed about the final result of the research project and the potential consequences for you:

Yes _____ (insert X) No _____ (insert X)

Consent from the study staff that provided the oral information:

I declare, that the participant has received both written and oral information about the research project.

I declare to the best of my knowledge and belief that the participant has received sufficient information to decide to participate in the research project.

Study staff name: ____

Date: _______________ Signature: ____

National project identification: H-20029882

For peer review only

Reporting checklist for protocol of a clinical trial

STANDARD PROTOCOL ITEMS: RECOMMENDATIONS FOR INTERVENTIONAL TRIALS

		Reporting Item	Page Number
**Administrative information**			
Title	#1	Descriptive title identifying the study design, population, interventions, and, if applicable, trial acronym	1
Trial registration	#2a	Trial identifier and registry name. If not yet registered, name of intended registry	2
Trial registration: data set	#2b	All items from the World Health Organization Trial Registration Data Set	N/A
Protocol version	#3	Date and version identifier	18,19
Funding	#4	Sources and types of financial, material, and other support	19
Roles and responsibilities: contributorship	#5a	Names, affiliations, and roles of protocol contributors	1,19
Roles and responsibilities: sponsor contact information	#5b	Name and contact information for the trial sponsor	19
Roles and responsibilities: sponsor and funder	#5c	Role of study sponsor and funders, if any, in study design; collection, management, analysis, and interpretation of data; writing of the report; and the decision to submit the report for publication, including whether they will have ultimate authority over any of these activities	18,19
Roles and responsibilities: committees	#5d	Composition, roles, and responsibilities of the coordinating centre, steering committee, endpoint adjudication committee, data management team, and other individuals	N/A

For peer review only

For peer review only - http://bmjopen.bmj.com/site/about/guidelines.xhtml

		or groups overseeing the trial, if applicable (see Item 21a for data monitoring committee)	
**Introduction**			
Background and rationale	#6a	Description of research question and justification for undertaking the trial, including summary of relevant studies (published and unpublished) examining benefits and harms for each intervention	4, 5
Background and rationale: choice of comparators	#6b	Explanation for choice of comparators	4, 5
Objectives	#7	Specific objectives or hypotheses	5
Trial design	#8	Description of trial design including type of trial (eg, parallel group, crossover, factorial, single group), allocation ratio, and framework (eg, superiority, equivalence, non-inferiority, exploratory)	5,6, Figure 1
**Methods: Participants, interventions, and outcomes**			
Study setting	#9	Description of study settings (eg, community clinic, academic hospital) and list of countries where data will be collected. Reference to where list of study sites can be obtained	5
Eligibility criteria	#10	Inclusion and exclusion criteria for participants. If applicable, eligibility criteria for study centres and individuals who will perform the interventions (eg, surgeons, psychotherapists)	6
Interventions: description	#11a	Interventions for each group with sufficient detail to allow replication, including how and when they will be administered	7-11
Interventions: modifications	#11b	Criteria for discontinuing or modifying allocated interventions for a given trial participant (eg, drug dose	6, 17

For peer review only

		change in response to harms, participant request, or improving / worsening disease)	
Interventions: adherance	#11c	Strategies to improve adherence to intervention protocols, and any procedures for monitoring adherence (eg, drug tablet return; laboratory tests)	11, 12
Interventions: concomitant care	#11d	Relevant concomitant care and interventions that are permitted or prohibited during the trial	6, 7, 11
Outcomes	#12	Primary, secondary, and other outcomes, including the specific measurement variable (eg, systolic blood pressure), analysis metric (eg, change from baseline, final value, time to event), method of aggregation (eg, median, proportion), and time point for each outcome. Explanation of the clinical relevance of chosen efficacy and harm outcomes is strongly recommended	5,14,15,16
Participant timeline	#13	Time schedule of enrolment, interventions (including any run-ins and washouts), assessments, and visits for participants. A schematic diagram is highly recommended (see Figure)	6, Figure 2
Sample size	#14	Estimated number of participants needed to achieve study objectives and how it was determined, including clinical and statistical assumptions supporting any sample size calculations	17
Recruitment	#15	Strategies for achieving adequate participant enrolment to reach target sample size	6
**Methods: Assignment of interventions (for controlled trials)**			
Allocation: sequence generation	#16a	Method of generating the allocation sequence (eg, computer-generated random numbers), and list of any factors for stratification. To reduce predictability of a random sequence, details of any planned restriction (eg, blocking) should be provided in a separate document that	7

For peer review only

		is unavailable to those who enrol participants or assign interventions	
Allocation concealment mechanism	#16b	Mechanism of implementing the allocation sequence (eg, central telephone; sequentially numbered, opaque, sealed envelopes), describing any steps to conceal the sequence until interventions are assigned	7
Allocation: implementation	#16c	Who will generate the allocation sequence, who will enrol participants, and who will assign participants to interventions	7
Blinding (masking)	#17a	Who will be blinded after assignment to interventions (eg, trial participants, care providers, outcome assessors, data analysts), and how	7
Blinding (masking): emergency unblinding	#17b	If blinded, circumstances under which unblinding is permissible, and procedure for revealing a participant's allocated intervention during the trial	N/A
**Methods: Data collection, management, and analysis**			
Data collection plan	#18a	Plans for assessment and collection of outcome, baseline, and other trial data, including any related processes to promote data quality (eg, duplicate measurements, training of assessors) and a description of study instruments (eg, questionnaires, laboratory tests) along with their reliability and validity, if known. Reference to where data collection forms can be found, if not in the protocol	12-16, Table 4
Data collection plan: retention	#18b	Plans to promote participant retention and complete follow-up, including list of any outcome data to be collected for participants who discontinue or deviate from intervention protocols	12

For peer review only

Data management	#19	Plans for data entry, coding, security, and storage, including any related processes to promote data quality (eg, double data entry; range checks for data values). Reference to where details of data management procedures can be found, if not in the protocol	17
Statistics: outcomes	#20a	Statistical methods for analysing primary and secondary outcomes. Reference to where other details of the statistical analysis plan can be found, if not in the protocol	17
Statistics: additional analyses	#20b	Methods for any additional analyses (eg, subgroup and adjusted analyses)	N/A
Statistics: analysis population and missing data	#20c	Definition of analysis population relating to protocol non-adherence (eg, as randomised analysis), and any statistical methods to handle missing data (eg, multiple imputation)	N/A
**Methods: Monitoring**			
Data monitoring: formal committee	#21a	Composition of data monitoring committee (DMC); summary of its role and reporting structure; statement of whether it is independent from the sponsor and competing interests; and reference to where further details about its charter can be found, if not in the protocol. Alternatively, an explanation of why a DMC is not needed	17
Data monitoring: interim analysis	#21b	Description of any interim analyses and stopping guidelines, including who will have access to these interim results and make the final decision to terminate the trial	17
Harms	#22	Plans for collecting, assessing, reporting, and managing solicited and spontaneously reported adverse events and other unintended effects of trial interventions or trial conduct	17
Auditing	#23	Frequency and procedures for auditing trial conduct, if any, and whether the process will be independent from investigators and the sponsor	N/A

For peer review only

**Ethics and dissemination**			
Research ethics approval	#24	Plans for seeking research ethics committee / institutional review board (REC / IRB) approval	18
Protocol amendments	#25	Plans for communicating important protocol modifications (eg, changes to eligibility criteria, outcomes, analyses) to relevant parties (eg, investigators, REC / IRBs, trial participants, trial registries, journals, regulators)	18
Consent or assent	#26a	Who will obtain informed consent or assent from potential trial participants or authorised surrogates, and how (see Item 32)	18
Consent or assent: ancillary studies	#26b	Additional consent provisions for collection and use of participant data and biological specimens in ancillary studies, if applicable	18
Confidentiality	#27	How personal information about potential and enrolled participants will be collected, shared, and maintained in order to protect confidentiality before, during, and after the trial	17, 18
Declaration of interests	#28	Financial and other competing interests for principal investigators for the overall trial and each study site	19
Data access	#29	Statement of who will have access to the final trial dataset, and disclosure of contractual agreements that limit such access for investigators	17, 18
Ancillary and post trial care	#30	Provisions, if any, for ancillary and post-trial care, and for compensation to those who suffer harm from trial participation	N/A
Dissemination policy: trial results	#31a	Plans for investigators and sponsor to communicate trial results to participants, healthcare professionals, the public, and other relevant groups (eg, via publication, reporting in results databases, or other data sharing arrangements), including any publication restrictions	18

For peer review only

Dissemination policy: authorship	#31b	Authorship eligibility guidelines and any intended use of professional writers	18
Dissemination policy: reproducible research	#31c	Plans, if any, for granting public access to the full protocol, participant-level dataset, and statistical code	N/A
**Appendices**			
Informed consent materials	#32	Model consent form and other related documentation given to participants and authorised surrogates	Supplemental Material
Biological specimens	#33	Plans for collection, laboratory evaluation, and storage of biological specimens for genetic or molecular analysis in the current trial and for future use in ancillary studies, if applicable	15, 16

The SPIRIT Explanation and Elaboration paper is distributed under the terms of the Creative Commons Attribution License CC-BY-NC. This checklist was completed on 02. March 2021 using https://www.goodreports.org/, a tool made by the EQUATOR Network in collaboration with Penelope.ai

For peer review only

BMJ Open

Empowering consumers to PREVENT diet-related diseases through OMICS sciences (PREVENTOMICS): Protocol for a parallel double-blinded randomised intervention trial to investigate biomarker-based nutrition plans for weight loss

Journal:	*BMJ Open*
Manuscript ID	bmjopen-2021-051285.R1
Article Type:	Protocol
Date Submitted by the Author:	29-Oct-2021

Complete List of Authors:	Aldubayan, Mona A.; University of Copenhagen Faculty of Science, Nutrition, Exercise and Sports; King Saud bin Abdulaziz University for Health Sciences, College of Applied Medical Sciences<br>Pigsborg, Kristina; University of Copenhagen Faculty of Science, Nutrition, Exercise and Sports<br>Gormsen, Sophia; Simple Feast<br>Serra, Francisca; University of the Balearic Islands, Laboratory of Molecular Biology, Nutrition and Biotechnology - NUO group; Alimentómica S.L., Spin-off n.1 of the University of the Balearic Islands<br>Palou, Mariona; University of the Balearic Islands, Laboratory of Molecular Biology, Nutrition and Biotechnology - NUO group; Alimentómica S.L., Spin-off n.1 of the University of the Balearic Islands<br>Mena, Pedro; University of Parma, Department of Food and Drug, Human Nutrition Unit<br>Wetzels, Mart; Onmi: Behaviour Change Technology<br>Calleja , Alberto; Grupo Carinsa<br>Caimari, Antoni; Eurecat Centre Tecnològic de Catalunya, Biotechnology Area, Nutrition and Health Unit<br>Del Bas, Josep; Eurecat Centre Tecnològic de Catalunya, Biotechnology Area, Nutrition and Health Unit<br>Gutierrez, Biotza; Eurecat Centre Tecnològic de Catalunya, Biotechnology Area, Nutrition and Health Unit<br>Magkos, Faidon ; University of Copenhagen, Faculty of Science, Nutrition, Exercise and Sports<br>Hjorth, Mads; Novo Nordisk Foundation, Healthy Weight Center

**Primary Subject Heading**:	Nutrition and metabolism
Secondary Subject Heading:	Public health
Keywords:	NUTRITION & DIETETICS, PUBLIC HEALTH, GENETICS

SCHOLARONE™
Manuscripts

Empowering consumers to PREVENT diet-related diseases through OMICS sciences (PREVENTOMICS): Protocol for a parallel double-blinded randomised intervention trial to investigate biomarker-based nutrition plans for weight loss

Mona A. Aldubayan[1,2], Kristina Pigsborg[1], Sophia M. O. Gormsen[3], Francisca Serra[4], Mariona Palou[4], Pedro Mena[5], Mart Wetzels[6], Alberto Calleja[7], Antoni Caimari[8], Josep M del Bas[8], Biotza Gutierrez[8], Faidon Magkos[1], Mads F. Hjorth[9]

[1]Department of Nutrition, Exercise and Sports, Faculty of Science, University of Copenhagen, Denmark.

[2]King Saud bin Abdulaziz University for Health Sciences, Riyadh, Saudi Arabia.

[3]Simple Feast, Copenhagen, Denmark.

[4]Laboratory of Molecular Biology, Nutrition and Biotechnology; NUO group. Universitat de les Illes Balears, Palma, Spain; Institut d'Investigació Sanitària Illes Balears (IdISBa), Palma, Spain; CIBER de Fisiopatología de la Obesidad y Nutrición (CIBEROBN), Madrid, Spain and Alimentómica S.L., Spin-off n.1 of the University of the Balearic Islands, Palma, Spain.

[5]Human Nutrition Unit, Department of Food and Drug, University of Parma, Parma, Italy.

[6]ONMI: Behaviour Change Technology, Eindhoven, The Netherlands.

[7]Grupo Carinsa, Sant Quirze del Valles, Barcelona, Spain.

[8]Eurecat, Centre Tecnològic de Catalunya, Biotechnology Area, Nutrition and Health Unit, Reus, Spain.

[9]Healthy Weight Centre, Novo Nordisk Foundation, Tuborg Havnevej 19, 2900 Hellerup, Denmark.

Corresponding author:

Mona Adnan Aldubayan
Department of Nutrition, Exercise and Sports,
Faculty of Science, University of Copenhagen
Rolighedsvej 26, 1958 Frederiksberg, Denmark
Phone: +45 91991923
E-mail: monal@nexs.ku.dk

Word count: 4947 (excluding title page, abstract, references, figures and tables).

For peer review only

ABSTRACT

Introduction: Personalised nutrition holds immense potential over conventional one-size-fits-all approaches for preventing and treating diet-related diseases, such as obesity. The current study aims to examine whether a personalised nutritional plan produces more favourable health outcomes than a standard approach based on general dietary recommendations in subjects with overweight or obesity and elevated waist circumference.

Methods and analysis: This project is a 10-week parallel, double-blinded randomised intervention trial. We plan to include 100 adults aged 18-65 years interested in losing weight, with body mass index ≥27 but <40 kg/m^2 and elevated waist circumference (males >94 cm; females >80 cm). Participants will be categorized into one of five predefined 'clusters' based on their individual metabolic biomarker profile and genetic background, and will be randomised in a 1:1 ratio to one of two groups: (1) personalised plan group that will receive cluster-specific meals every day for 6 days a week, in conjunction with a personalised behavioural change program via electronic push notifications; or (2) control group that will receive meals following the general dietary recommendations in conjunction with generic health behaviour prompts. The primary outcome is the difference between groups (personalised vs. control) in the change in fat mass from baseline. Other outcome measures include changes in body weight and waist circumference, lipid profile, fasting blood glucose, adipokines, inflammatory biomarkers, blood pressure, physical activity and sleep patterns, health-related quality of life, eating behaviour, attitude to weight management diets and dietary intake. The effect of the intervention on the primary outcome will be analysed by means of linear mixed models.

Ethics and dissemination: The protocol has been approved by the Ethics Committee of the Capital Region, Copenhagen, Denmark. Study findings will be disseminated through peer-reviewed publications, conference presentations and media outlets.

Trial registration number: ClinicalTrials.gov registry (NCT04590989).

Keywords: Personalised nutrition, Precision nutrition, Nutrigenomics, Nutrigenetics, Metabolomics, Obesity, Overweight, Weight management, Body weight

For peer review only - http://bmjopen.bmj.com/site/about/guidelines.xhtml

Strengths and limitations of this study

- This study may identify novel approaches in facilitating weight loss and health-promoting behaviours by applying state-of-the-art knowledge that integrates metabolomics and genetics with nutrition.
- The trial is double-blinded, which is rare in nutritional science, and serves as proof of concept for the personalised dietary management of obesity.
- A potential limitation is that both groups are receiving healthy foods and behavioural advice, which may mask the hypothesised intervention effect of the personalised plan.
- The study is powered to detect differences in 10-week body fat loss between intervention and control arms and not within each of the five clusters; differences between the latter will be assessed by post-hoc analysis.
- Potential long-term effects of a personalised approach cannot be evaluated from this 10-week study; however, results will provide a basis for implementation in longer obesity-management programs.

For peer review only - http://bmjopen.bmj.com/site/about/guidelines.xhtml

INTRODUCTION

The ultimate goal of nutrition research and dietary recommendations is the promotion of human health and the prevention or treatment of chronic diseases.[1] Still, the global prevalence rate of nutrition-related non-communicable diseases (NCDs) continues to rise rapidly.[2] There is considerable evidence indicating that obesity is a major risk factor for developing NCDs including type 2 diabetes, cardiovascular diseases, and certain types of cancers, which are the leading causes of morbidity and mortality.[3 4] Therefore, obesity puts a great burden on the individual, the healthcare system, and society.[3] Accordingly, enormous efforts to tackle this epidemic have been implemented from health professionals through setting different policies and guidelines for the public, but with little success, as management of obesity remains a very challenging task. Moreover, the optimal diet characteristics—particularly with respect to dietary macronutrient composition (e.g., low-carbohydrate, low-fat, high-protein diets)—that are most effective in reducing excess weight gain or promoting weight loss have long been debated.[5 6] Clinical trials have demonstrated that certain individuals benefit more from a particular dietary intervention than others in reducing body weight, while only a small number are able to keep the weight off in the long-term.[7 8] This implies there is no strong evidence that one diet is superior to others for inducing weight loss, and there is no such thing as a “perfect” diet for everyone. Such substantial interindividual variation in response to any given dietary treatment can be attributed to multiple phenotypic factors and genetic variants which influence how the body utilises and metabolises nutrients.[7] This gives rise to the demand for customising diet plans and nutrition advice at the individual or small group level, rather than at the population level. Recent developments in “omics” technologies (nutrigenomics, transcriptomics, epigenomics, metabolomics, metagenomics) offer exciting opportunities to explore the complex interplay between nutrition, genetics and metabolism.[9] By integrating these novel tools with bioinformatics, the potential of “personalised nutrition” can be implemented through identifying novel biomarkers that can predict the most effective diet for weight loss and improved health outcomes for any given individual.[9-11] Therefore, the ability to provide evidence-based dietary advice based on individual genetic make-up, phenotypic information on anthropometry, biochemical and metabolic profiles, physical activity habits, and medical history—among others—may lead to changed behaviours and ultimately, improved health.

In this context, H2020 PREVENTOMICS (Empowering consumers to PREVENT diet-related diseases through OMICS sciences), coordinated by Eurecat in Spain, has developed a platform with a Decision Support System (DSS) tool that integrates individual phenotypic characteristics at the metabolome level with their genotype, lifestyle habits, and preferences to improve their health status through personalised nutrition management plans. The project aims to examine the validity of the PREVENTOMICS platform in terms of its

For peer review only - http://bmjopen.bmj.com/site/about/guidelines.xhtml

potential for personalisation at different levels of the food value chain. This will be achieved through different intervention studies in Denmark, Spain, and Poland and the United Kingdom, with both healthy volunteers and volunteers with abdominal obesity. Here we report the specific characteristics of the Danish study protocol.

Research hypothesis and aims

The overall aim of this 10-week randomised trial is to examine the efficacy of the PREVENTOMICS platform, integrated in an e-commerce digital tool created for delivering personalised meals for producing more favourable health outcomes than meals based on general dietary recommendations, in subjects with overweight or obesity and elevated waist circumference. In addition, the intervention group will receive tailored and actionable behaviour change prompts whereas the control group will receive general nutrition and lifestyle advice. Our hypothesis is that the personalised dietary and behavioural treatment plan will produce greater reductions in fat mass and body weight, and will promote more favourable changes in circulating metabolic and inflammatory biomarkers compared to the control dietary and behavioural treatment plan.

Primary and secondary objectives

The primary goal of this study is to evaluate the change in body fat mass between the personalised plan group and the control group over the 10-week intervention period. The secondary goal is to evaluate the change in the following health outcomes between the personalised and control groups: (1) body composition (visceral and subcutaneous fat, lean body mass, weight, body mass index [BMI], waist circumference); (2) lipid profile (total cholesterol, low-density lipoprotein [LDL], high-density lipoprotein [HDL], oxidized LDL [oxLDL], triglycerides); (3) glucose homeostasis (glucose, insulin, homeostatic model assessment of insulin resistance [HOMA-IR]); (4) inflammatory markers (C-reactive protein [CRP], interleukin 6 [IL-6], interleukin 10 [IL-10], monocyte chemoattractant protein-1 [MCP-1], tumour necrosis factor alpha [TNFα], soluble amino acid residue glycosylated peptide-1 [ICAM-1], soluble cluster of differentiation-14 [CD-14]); (5) adipokines (leptin, adiponectin); (6) liver health markers (alanine transaminase [ALT], gamma-glutamyl transferase [GGT]); (7) renal health markers (uric acid, creatinine); and (8) blood pressure.

For peer review only - http://bmjopen.bmj.com/site/about/guidelines.xhtml

METHODS AND ANALYSIS

Study design

This is a randomised, single-centre, parallel-group (1:1 ratio), double-blinded intervention study conducted at the research facilities of the Department of Nutrition, Exercise and Sports (NEXS), University of Copenhagen, Denmark. The study protocol adheres to the SPIRIT (Standard Protocol Items: Recommendations for Interventional Trials) guidelines.[12] This study was registered at ClinicalTrials.gov in October 2020 (NCT04590989), and recruitment started at NEXS immediately after. Information needed to determine the 'cluster' of participants was collected in January 2021, and the actual analysis of biological samples and clustering was performed in February-March 2021. All data collected during the 10-week intervention period (March-June 2021) are expected to be fully analysed by the end of 2021. The overall study design is illustrated in Figure 1.

Patient and Public Involvement: Patients and the public were not involved in the design, conduct or reporting of this study.

Study participants

Participants are males and females aged 18–65 years with a BMI of ≥27 but <40 kg/m^2 and elevated waist circumference (males >94 cm; females >80 cm). Participants should possess a smartphone and be able to provide an informed consent. The exclusion criteria are as follows: (1) diagnosis of diabetes; (2) history or diagnosis of heart, liver or kidney diseases; (3) chronic diseases, e.g., cancer within the past 5 years (except adequately-treated localized basal cell skin cancer); (4) use of drugs (e.g., antibiotics) that, in the opinion of the medically responsible investigator, are likely to affect the primary outcomes of the study; (5) being lactating, pregnant or planning to become pregnant within the study period; (6) self-reported weight change of >5 % within two months prior to screening; (7) participation in another clinical trial; (8) other blood donation during the study; (9) having allergies or food intolerances; (10) no or limited access to the internet. Participants unable to comply with the study protocol, as judged by the investigator, are also excluded.

Recruitment procedure

The study flow chart is summarized in Figure 2. Potential participants were recruited through internet-based advertisements. Trained study personnel contacted 220 subjects who expressed interest in the study via telephone to determine initial eligibility (pre-screening). Written information about the study was provided to 120 potential participants who were deemed eligible from the telephone pre-screening and

For peer review only - http://bmjopen.bmj.com/site/about/guidelines.xhtml

scheduled for an oral information meeting (visit 0, V0) at the department (NEXS). If the subject signed the informed consent, either immediately following the information meeting or after a few days of consideration, they were screened according to the inclusion/exclusion criteria to assess final eligibility. A total of 106 participants were recruited and invited for the pre-baseline visit (V1) where anthropometric measurements, blood, saliva, and urine samples were collected, and various questionnaires were filled out. One hundred participants completed V1 and had their samples sent to the assigned consortium for analysing data on subjects' metabolome and genotype in addition to lifestyle habits, preferences, and physiological status. These data are utilized to determine subjects' cluster (see later) and develop the personalised dietary plans for the subsequent 10-week intervention period.

Cluster allocation

All subjects were categorized into one of five predefined 'clusters' (Table 1) based on their metabolic and genetic biomarkers collected at V1, according to the following procedure:

First, collected samples of urine, plasma and serum were analysed to assess a total of 58 biomarkers relevant to the following five metabolic processes: 1) oxidative stress; 2) inflammation; 3) carbohydrate metabolism; 4) lipid metabolism; and 5) gut microbiota metabolism. References supporting the rationale for using the biomarkers listed in Table 1 are included in the online supplemental material 1.

Table 1: Full list of biomarkers in relation to the metabolic clusters.

Carbohydrate	Lipid	Inflammation	Oxidative stress	Microbiota
Glucose	LDL-cholesterol	CRP	8-iso-PGF2α	TMA
HOMA-IR	Total cholesterol	N-acetylglycoproteins	8-OHdG	TMAO
Glutamate	PUFAs	MCP-1	oxidized LDL	Betaine
Uric acid	HDL-cholesterol	TNFα	Uric acid	Choline
Leptin	SFAs	IL-6	Allantoin	DMA
Adiponectin	Triglycerides	IL-10	Betaine	Dimethylglycine
Insulin	MUFAs	SFAs	Pseudouridine	LBP
Tyrosine	LPC	sICAM-1	Dimethylglycine	Succinate
Propionylcarnitine	Linoleic acid	LPC	Methionine	Lactate
Lactate	DHA	LBP	Glycine	Acetate
Valine	Oleic acid	DHA C20:3		
Leucine	Choline	sCD14		
Isoleucine	3-hydroxybutyrate	Linoleic acid C18:2		
Phenylalanine	Propionylcarnitine	PUFAs		

For peer review only - http://bmjopen.bmj.com/site/about/guidelines.xhtml

Glutamine	Adiponectin
	Leptin

Some biomarkers help define more than one cluster. Abbreviations: HOMA-IR, homeostatic model assessment of insulin resistance; LDL, low-density lipoprotein; HDL, high-density lipoprotein; PUFAs, polyunsaturated fatty acids; MUFAs, monounsaturated fatty acids; SFAs, saturated fatty acids; LPC, lysophosphatidylcholine; DHA, docosahexaenoic acid; CRP, C-reactive protein; MCP-1, monocyte chemoattractant protein-1; TNFα, tumour necrosis factor alpha; IL-6, interleukin-6; IL-10, interleukin-10; sICAM-1, soluble amino acid residue glycosylated peptide-1; sCD14, soluble cluster of differentiation-14; LBP, lipopolysaccharide binding protein; 8-iso-PGF2α, prostaglandin, 8-iso-prostaglandin F2α; 8-OHdG, 8-hydroxydeoxyguanosine; TMA, trimethylamine; TMAO, trimethylamine N-oxide; DMA, dimethylamine.

Second, a core of 35 different single nucleotide polymorphisms (SNPs) which are associated with the five metabolic processes and are able to modulate the biomarker levels reported in Table 1 have been identified by Alimentomica (Spain) to be analysed in saliva samples (Table 2). The biomarkers of the lipid cluster are able to be modulated, at different degree, by a set of 18 SNPs in 14 genes[13-20], the carbohydrate cluster is represented by a panel of 12 SNPs in 11 genes[14 15 21-24] and the inflammation cluster by 8 SNPs in 7 genes.[25-28] Parse scientific data deal with the genetic impact on the specific biomarkers of the oxidative stress cluster; the corresponding genetic risk score comprises 7 genetic variants in 6 genes associated with reduced ability to buffer the oxidative stress associated with low levels of plasma antioxidants.[29-33] In relation to the microbiota cluster, the current evidence base does not provide enough data in support of the role of SNPs (included in the panel or not) and some evidence concerning the host genetic influence on microbiota response and on microbiota metabolite production is neither robust nor sufficient; hence no genetic influence was adopted in this cluster.

Finally, the specific SNPs and the biomarkers in the five metabolic processes will be used—by means of a proprietary algorithm—to calculate individual scores for each of the five metabolic clusters for any given participant. Each subject is then assigned to the metabolic cluster with the highest score. Briefly, the individual biomarkers, both metabolic and genetic, are combined into metabolic clusters considering both the absolute value of the biomarker in the biofluid and the biological relevance of the biomarker within the metabolic cluster. Whereas the first value is directly obtained from blood and urine measurements (metabolomics and proteomics biomarkers) and saliva (genotyping), the second is obtained from different approaches combining artificial intelligence applied to measurements of different biobank samples and literature review.[34] Therefore, the resulting score for each cluster is not based on the definition of thresholds but on the contribution of all individual biomarkers analysed. The specifics of the algorithm cannot be disclosed due to a pending intellectual property rights (IPR) application.

For peer review only - http://bmjopen.bmj.com/site/about/guidelines.xhtml

Table 2: List of SNPs in relation to the metabolic clusters

Lipid		Carbohydrate		Oxidative		Inflammation	
**Gene**	**SNP**	**Gene**	**SNP**	**Gene**	**SNP**	**Gene**	**SNP**
ADIPOQ	rs182052	ADIPOQ	rs182052	COMT	rs4680	APOE	rs429358
APOA5	rs12272004	ASCL1	rs17450122	CPS1	rs1047891	CADM3-AS1	rs12075
APOA5	rs662799	FADS1, FADS2	rs174550	CPS1	rs715	CUX1	rs409224
APOE	rs7412	GCKR	rs1260326	FGF21	rs838133	FADS1	rs174547
APOE	rs429358	GCKR	rs780093	GSTP1	rs1695	GCKR	rs1260326
CUX1	rs409224	GLS2, SPRYD4	rs2657879	MTHFR	rs1801133	GCKR	rs780093
FADS1	rs174547	LEP	rs10487505	SOD2	rs4880	ICAM1	rs5498
GCKR	rs780093	PPARG	rs1801282			IL-6	rs1800795
GCKR	rs1260326	SLC16A10	rs14399				
HFE	rs1800562	SLC16A9	rs1171614				
LEP	rs10487505	SLC2A2	rs8192675				
LPL	rs268	TCF7L2	rs7903146				
LPL	rs326						
PNPLA3	rs738409						
PPID	rs8396						
SLC16A9	rs1171614						
TIMP3	rs12678919						
TRIM58	rs3811444						

Randomisation and concealment

Study participants are randomly allocated in a 1:1 ratio, stratified by cluster, to either the intervention group (personalised plan) or the control group (generic recommendations) prior to the intervention period. The allocation is computer-generated and the person responsible for generating the code does not take part in the inclusion and examination of study participants.

In order to maintain blinding, the clustering results are shared by Eurecat before the baseline visit (V2) with a member of the staff at NEXS who is responsible for randomisation; consequently, the allocation information is incorporated into each subject's profile in the PREVENTOMICS platform. Afterwards, Eurecat will grant access to Simple Feast (Denmark) and ONMI (The Netherlands; behavioural change technology, https://www.onmi.design/) to the subjects' profiles via the platform for appropriate delivery of food and behavioural prompts. The profile includes the assigned group (personalised or control) and cluster, in addition to relevant recommendations to each subject. The local team of investigators at NEXS is therefore unaware of the clustering and the randomisation of the participants. Moreover, the statistical analyses of the main outcome variable will be conducted without breaking the code for the intervention treatment until the primary analyses have been finalized.

For peer review only - http://bmjopen.bmj.com/site/about/guidelines.xhtml

Interventions

1. Dietary intervention

During the 10-week intervention, the personalised plan and control groups receive easy-to-prepare meal boxes twice a week from Simple Feast (Copenhagen, Denmark) complying with the national dietary guidelines of macronutrient distributions.[35] Each delivery provides meal boxes of breakfast and dinner for the subsequent three days (12 meals/week). Meal boxes for the two groups are designed to be visually identical. Moreover, food by Simple Feast is vegetarian and organically produced, however, participants are allowed to eat non-organic/non-vegetarian foods as part of the meals not provided. The number of meals provided to the participants was decided upon a combination of factors including budgetary limitations, practical reasons, and behavioural factors. For the days for which meals are not provided (Saturdays), as well as for all lunches, participants are encouraged to refer to the recipe recommendations that are presented through the Simple Feast Recipe App, so they prepare meals as similar as possible to the group and cluster they are assigned to.

The calorie content of meals was calculated based on the average daily energy requirements for the general population, which is 2000 kcal/day for females and 2500 kcal/day for males.[36] Given that 25% of daily energy is commonly consumed at breakfast and 35% at dinner, breakfast was designed to provide approximately 500 and 625 kcal/day (for females and males, respectively) and dinner to provide approximately 700 and 875 kcal/day (for females and males, respectively). Participants are instructed to consume the food provided ad libitum, until being fully satisfied. We anticipate this plant-based ad libitum diet to result in eating less outside the provided meals (i.e., during lunch or when snacking) and thereby produce the calorie deficit needed for body weight and fat loss. It is also anticipated that the personalised plan induces favourable changes in eating behaviour and physiological and metabolic parameters that promote body weight and fat loss when compared to the control plan.

The Eurecat Nutrition Team has prepared a list of recommended food items to increase, decrease, or completely exclude from the diet for the control and each cluster in the personalised group. The list was adopted by Simple Feast in creating five different menus that encompass 12 meals/week for the five different clusters in the intervention group in addition to the menu for the control group. Contrary to the control meals, personalised meals also include some bioactive compounds provided by CARINSA (Spain). These bioactive compounds were selected following review of the literature and are believed to benefit especially—or exclusively—the metabolic function of individuals in the corresponding cluster (Table 3). Each participant received approximately 20 g of functional ingredient per day, except for the inflammation cluster (6-8 g per day). The macronutrient distribution of the diets between clusters differed only in the

For peer review only - http://bmjopen.bmj.com/site/about/guidelines.xhtml

amount of fibre. Dietary fibre content was higher in the Carbohydrate and Microbiota clusters as these clusters received fructooligosaccharide (FOS) and inulin as functional ingredients. Nutritional information on macronutrient content and the bioactive ingredients of the meals for each cluster in the personalised and control groups, as well as an example of a 3-day menu, are provided in the online supplementary material 2.

Table 3: Recommended foods and functional ingredients for each metabolic cluster

**Carbohydrate**	Functional ingredient: *FOS and †Inulin<br>Prebiotics: fibre-rich plants (Jerusalem artichoke, onion, leek, asparagus, kale)
**Microbiota**	Functional ingredient: *FOS and †Inulin<br>Prebiotics: fibre-rich plants (Jerusalem artichoke, onion, leek, asparagus, kale)<br>Fermented vegetables<br>Vegetables rich in fibre
**Lipid**	Functional ingredient: sunflower oil<br>Raw nuts and seeds<br>Omega 3 and 6: chia seeds, hemp seeds, walnuts, flax seeds<br>Vegetables rich in fibre
**Inflammation**	Functional ingredient: turmeric powder<br>Raw nuts and seeds<br>Omega 3 and 6: chia seeds, hemp seeds, walnuts, flax seeds<br>Dark chocolate
**Oxidative stress**	Functional ingredient: oleic acid enriched sunflower oil.<br>Raw nuts and seeds<br>Orange, yellow, red coloured vegetables (rich in vitamin A, C, E)<br>Dark chocolate<br>Vegetables rich in fibre

*Fructooligosaccharide originates from partial hydrolysis of chicory roots. †Inulin is extracted from chicory roots.

2. Behavioural assessment and intervention

All participants are asked to fill out a behavioural questionnaire at baseline (V2), in order to collect information about certain habits or behaviours that affect physical, emotional, or mental well-being. During the 10-week intervention period, both groups are enrolled in a behavioural program delivered through ONMI's App with 2-3 electronic push notifications per week. Subjects randomised to the personalised group receive behavioural prompts (active Do's) from the predefined ONMI's evidence-based behavioural change program, which has been developed to increase behavioural flexibility and facilitate adoption of healthier habits.[37] For the purposes of this trial, the personalised group Do's (from ONMI) are based on subject's reports from the behavioural questionnaire at V2 in addition to inputs from the nutritional recommendations (from the Eurecat Nutrition Team) via the PREVENTOMICS platform, to provide a comprehensive behavioural change and improve adherence to the dietary intervention. For example, if a participant was recommended to eat kale and Brussels sprouts, they could get a message like: "*Our analysis shows kale and brussels sprouts are good for you and should be part of your diet. Find out how*

For peer review only - http://bmjopen.bmj.com/site/about/guidelines.xhtml

much you should be consuming. Do it now". Table 4 illustrates the different types and quantity of the Do's and massages delivered to personalised and control groups. The messages delivered to the control group are not personalised and are mostly informational in nature rather than prompting participants to take a specific action (i.e., general guidelines available from the National Health Service and the World Health Organization). The personalised and control groups receive the same behavioural treatment in terms of volume (frequency and intensity); the content of messages differs between groups as reflected in the numbers of specific types of messages delivered to each group, but the total number of messages is very similar (Table 4).

Table 4: The type and number of behavioural messages delivered by ONMI to the participants in the personalised and control groups

Type of Messages	Quantity	Description	Example	PP	C
Starter Do	1	Easy start of the program upon behavioural questionnaire completion at V2	SWITCH SEATS DAY! Move some seating around today. Sit somewhere different at meals/ when working/when watching TV. Get a new view! -- Shaking up old habits is good for you and puts you back in charge of your life. Try something new regularly. Make every day count! --	✓	✓
General Do	5	Apply to everyone, relatively easy, to get user hooked to the program	NEW WAY DAY. Take a detour today, go the prettiest route not the shortest. Allow more time, smile at people. Spot 3 beautiful things along the way. -- Wakey Wakey. Regularly challenging our brain keep us alert and interesting. When we take notice of our surroundings we start to live life to the fullest.	✓	
Personalised Do	10	Based on behavioural questionnaire	WHAT ARE YOU EATING FOR? Back off from boredom, address your stress. Get busy, unwind, release your emotions so you only eat when you're hungry today.	✓	
System Message	3	Encouragements, tips, manage expectations	HEALTH TIP. Regular contact with friends and family is key to good mental and physical health. Connections give meaning and purpose to our lives, even when it is digitally.	✓	
Expander Do	3	Prompt user to explore new parts of personality, based on behavioural questionnaire	EXPANDER: It's NO Day today. Don't say yes when you really want to say no. Give no reason or excuse. Just say, 'Sorry, but the answer's no'.	✓	
Preventomics Messages	6	Template messages that use inputs from the nutritional recommendations of food to increase	PREVENTOMICS: Are you getting the right amount of {{.R1}} and {{.R2}} in your diet? Go online and find some interesting recipes to try at home. Do it now.	✓	
General Messages	24	Recommendations from the NHS and WHO on eating,	Eating a healthy, balanced diet is an important part of maintaining good health, and can help you feel your best. This means eating a wide		✓

For peer review only - http://bmjopen.bmj.com/site/about/guidelines.xhtml

		eating out, exercise, check-ups, help and support, balanced diet	variety of foods in the right proportions, and consuming the right amount of food and drink to achieve and maintain a healthy body weight.		

Abbreviations: PP, personalised plan; C, control; NHS, The National Health Service; WHO, World Health Organization.

Compliance and food intake biomarkers

Dietary adherence is assessed twice a week—through an electronic questionnaire—by reporting the proportion of food consumed from the meals provided by Simple Feast in the previous three days. For example, in response to the question "*How much of your breakfast did you eat on day 1?*" the possible answers can be: a) Nothing or very little (0-30%); b) Approximately half (30-70%); c) Almost everything or everything (70-100%).

Overall compliance to the diet and the behavioural program is measured at the end of the trial by a six-point Likert scale question ranging from 1 (not at all compliant) to 6 (completely compliant). In addition, objective measures of adherence to the nutrition intervention will be evaluated: urine collected at pre-baseline and the end-of-trial visits will be analysed after the intervention for selected biomarkers of food intake through a target ultra-performance liquid chromatography-ion mobility separation-high resolution mass spectrometry (UPLC-IMS-HRMS) approach at the University of Parma (UNIPR, Italy). Data on biomarkers of food intake will also serve to assess the validity of the information collected through 3-day food diaries at pre-baseline and end-of-trial visits.

Data collection

Data are collected at screening, pre-baseline (V1), baseline (V2), and 10 weeks after initiating the intervention (V3) by using self-reported questionnaires, biological specimens, accelerometers, and physical examinations conducted by trained local research staff in accordance with standard operating procedures (Table 5). All primary and secondary endpoints are derived from measurements obtained at baseline (V2) and at the end of the intervention (V3). Measurements taken pre-baseline (V1) were used for clustering assignment. Reminder text messages are being sent to the participants before visit days. It is expected that a high retention rate will be achieved due to the nature of the study.

Primary outcome

The primary endpoint is the difference in body fat mass (kg) from baseline to end of trial between the two intervention groups (personalised vs. control). Body fat mass is determined during body composition analysis by use of dual-energy X-ray absorptiometry (iDXA, Lunar Radiation Co., Madison, Wisconsin, USA). The participants are scanned in a fasted state, lying on their back wearing lightweight clothes without jewellery and other metallic objects.

For peer review only - http://bmjopen.bmj.com/site/about/guidelines.xhtml

Table 5: Procedures and activities during the study period

	Screening visit (V0)	Pre-baseline visit (V1)	Baseline visit (V2)	End of trial visit (V3)
**Week**	**-20 to -13**	**-9 to -6**	**0**	**10**
**Visit day**	**0**	**1**	**2**	**3**
Informed consent	X			
Review of inclusion and exclusion criteria	X			
Medical history & examination	X			
Registration of medication and adverse events	X	X	X	X
**Anthropometry**				
Body weight	X	X	X	X
Height	X			
Waist circumference	X	X	X	X
Body composition (DXA)			X	X
**Biological Samples**				
Fasting blood sample		X	X	X
Saliva sample (SNPs)		X		
Urine sample		X	X	X
Faecal sample			X*	X*
**Nutritional Assessment**				
Food frequency questionnaire (FFQ)		X		X
3-day dietary records		X*		X*
**Other measurements**				
Blood Pressure/heart rate		X	X	X
Three factor eating questionnaire (TFEQ)			X	X
Perceived stress scale (PSS)			X	X
Behavioural questionnaire (by ONMI)			X	X
Quality of life questionnaires			X	X
Diet satisfaction questionnaire (DSat-28)			X	X
Money spent on food questions			X	X
Accelerometer (sleep and PA)		X**	X**	

X*: At home activity prior to visit.
X**: At home activity following the visit.

For peer review only - http://bmjopen.bmj.com/site/about/guidelines.xhtml

Secondary outcomes

Anthropometry

Body weight is measured at all visits using a calibrated digital scale to the nearest 0.1 kg with participants wearing lightweight clothes and no shoes, as well as after voiding their bladder. Height is measured at the screening visit using a wall-mounted stadiometer to the nearest 0.5 cm while participants are not wearing shoes. Body mass index (kg/m^2) is calculated as weight in kilograms divided by height in meters squared. Waist circumference (cm) is measured with a stretch-resistant tape at the midpoint between the lower margin of the last palpable ribs and the top of the iliac crest, in the fasted state (non-fasted during the screening visit) with an empty bladder and with participants wearing light clothes. Each measurement is taken twice to the nearest 0.5 cm and the average is used.

Biological Samples

Fasting blood samples collected at pre-baseline, baseline, and week 10 are analysed for plasma glucose, insulin, adipokines (leptin and adiponectin), inflammatory biomarkers (CRP, IL-6, IL-10, TNFα, MCP1, sICAM1, sCD14), lipid profile (total cholesterol, LDL-cholesterol, HDL-cholesterol, oxLDL, triglycerides), liver biomarkers (ALT and GGT), and renal biomarkers (uric acid, creatinine). Blood samples are sent to Eurecat for metabolomics analysis while biochemical markers are measured at NEXS, University of Copenhagen.

peer review

Blood pressure

Systolic and diastolic blood pressures and heart rate are measured by an automatic sphygmomanometer on the arm after 5-10 min rest in a sitting position. The same arm is used during all visits. The measurement is repeated three times (or four, if the last two measurements deviate by >5 mmHg), approximately 1 minute apart, and readings are recorded to the nearest 1 mmHg for blood pressures and 1 bpm for heart rate. The average of the last two readings is used.

Other outcome measures

Saliva

Saliva samples were collected pre-baseline and sent to Alimentomica (University of the Balearic Islands, Palma de Mallorca) for analysis of genetic variants, mainly single nucleotide polymorphisms (SNPs) in genes related to metabolism, inflammation, and oxidative stress for cluster assignment. Currently, there are 188 candidate SNPs being analysed and validated in an ongoing trial and a minimum of 35 and maximum of 150 SNPs of those are expected to be used in the present study.

For peer review only - http://bmjopen.bmj.com/site/about/guidelines.xhtml

Urine samples

Participants were asked to deliver a second-void urine spot sample in the morning at pre-baseline, and do the same at baseline and end of the study visits. Urine samples from pre-baseline and week 10 are used by UNIPR for food intake biomarker analysis and by Eurecat for analysing markers of oxidative stress utilized in cluster assignment. Furthermore, aliquots of urine samples from all visits are stored in a biobank for future analyses.

Nutritional assessment

Dietary intake is assessed pre-baseline and at the end of the study by using a validated self-administered electronic form of the European Prospective Investigation of Cancer (EPIC)-Norfolk Study food frequency questionnaire (FFQ),[38] supervised by a trained staff member. Furthermore, participants are instructed to complete 3-day weighed food records during the week before the pre-baseline visit and during the third week of the intervention. The dietary records cover two non-consecutive weekdays and one weekend day. Nutrient analysis will be done by the software program Vitakost (Conava ApS; Kolding, Denmark), which is based on the Danish national food database.

Questionnaires

Eating behaviour assessment at baseline and week 10 is conducted by administrating the three factor-eating questionnaire (TFEQ).[39] This is a 51-item self-report questionnaire which measures three domains of eating behaviour: (1) cognitive restraint of eating, (2) disinhibition and (3) hunger.

Stress assessment is conducted through the 10-item perceived stress scale (PSS)[40] at baseline and week 10. The PSS is one of the most widely used psychological instruments. It measures the degree to which participants perceive events in their life as being stressful by asking about thoughts and feelings over the last month using a response scale from 0 (never) to 4 (very often).

Quality of life is assessed by two questionnaires at baseline and week 10: 1) EQ-5D-5L[41], which is a standardized instrument developed by EuroQol Group for measuring health-related quality of life on five dimensions (mobility, self-care, usual activities, pain/discomfort, and anxiety/depression), with five response levels per dimension, and also includes a visual analogue scale (EQ-VAS) by which respondents report their perceived health status; and 2) Obesity and Weight-Loss Quality of Life Instrument (OWLQOL)[42], which is an instrument consisting of 17 statements about weight-related feelings and emotions which are rated on a seven-point scale, and primarily measures emotions and feelings resulting from suffering from obesity and trying to lose weight.

For peer review only - http://bmjopen.bmj.com/site/about/guidelines.xhtml

Diet satisfaction is assessed at baseline and week 10 by the Diet Satisfaction Questionnaire (DSat-28)[43] that involves 28 statements grouped into five dimensions (healthy lifestyle, eating out, cost, preoccupation with food, and planning and preparation) to evaluate satisfaction with weight-management diets.

Food expenditure is assessed by two questions completed at baseline and week 10 regarding the amount of money spent on food for the whole household.

Physical activity and sleep patterns are determined by ActiGraph GT3X+ accelerometer (ActiGraph, LLC, Pensacola, FL, USA) for 7 days/8 nights immediately following the pre-baseline visit and again for 7 days/8 nights during the third week of the intervention. During these wear-periods, a self-administered sleep-log to assess bedtimes is also obtained.

Microbiota composition will be determined on faecal samples collected within 24 hours prior to the clinical investigation days at baseline and week 10. These samples are not used in the cluster assignment but are collected and stored for future analyses.

Data management

Study investigators have access to the data collection forms and protocols using a secure shared drive. All collected data are pseudo-anonymized and participants are identified only by a study ID number on documents and on electronic databases, with personal identifiers kept separately under strict access control, limited to investigators and study staff directly involved in data collection and entry. All biological specimens sent to the consortium partners are pseudo-anonymized and encrypted. Upon completion of the study, data will be stored in a password-protected database, accessible only by study investigators, in anonymous form for a minimum of 10 years.

Data Monitoring

As the intervention risks to participants are minimal, a Data and Safety Monitoring Board was not deemed necessary. However, in case of unexpected adverse events during the study period, these are recorded and brought up to the principal investigator for appropriate decision-making. We did not plan to conduct interim analysis for safety as we did not anticipate any serious adverse events that would require trial termination.

Power and sample size calculation

To detect a difference in body fat mass change of 1.25 kg between the two intervention groups with 80% power at a two-tailed level of significance of 0.05, assuming a standard deviation (SD) of 2.0 kg, a sample size of 41 per group (i.e., personalised vs. control) is needed, i.e., a total sample size of 82 completers. To allow for an anticipated 18% dropout rate, 50 subjects per group would need to be recruited (total n = 100). The expected difference in fat mass between groups (1.25 kg) and the associated SD (2.0 kg) were based on values calculated from the raw data of the SHOPUS study.[44] In that study, we reported on body weight and fat mass during a 6-month dietary intervention in subjects with BMI of 22.6-47.3 kg/m^2. To conduct power calculations for this study, we extracted raw data from the SHOPUS study that were most representative of the current subjects and intervention duration. Accordingly, we selected those SHOPUS participants with BMI ≥27 kg/m^2 and elevated waist circumference (males >94 cm; females >80 cm) and assessed their body weight and fat mass at 12 weeks; this was an interim time point of the SHOPUS study (not published in the original paper),[44] which was the closest to the 10 week time frame of the current intervention.

Statistical analysis

Data analysis will be conducted using SPSS and R. Before statistical analyses are conducted, all continuous variables will be tested for normality and homogeneity of variance. For our primary objective, differences in fat mass from baseline to end of trial (V3 minus V2) between the two intervention groups (personalised vs. control) will be compared by means of linear mixed models with time and randomisation group as main effects, a time-by-group interaction, and adjusting for potential confounders (e.g., sex, age, and BMI at baseline) as necessary. If significant interactions emerge, post hoc testing will be used to evaluate effects within the metabolic clusters. LMMs are able to handle possible imbalances between groups in case of missing values in a single response variable.

Ethics and dissemination

The study has been approved by the Regional Committees on Health Research Ethics, Region Hovedstaden in Denmark (H-20029882) and is being conducted in accordance with the Helsinki Declaration. Any protocol amendments are submitted to the Research Ethics Board for approval and communicated to study participants and the trial registry once approved.

All personal data is being handled confidentially and stored in accordance with applicable law, GDPR and Danish Data Protection Agency. Participants received written and oral information on the study procedures, and only trained study-personnel provided information, monitored and attested signing of the informed consent form (online supplemental material 3). In addition, an optional GDPR consent to provide excess

For peer review only - http://bmjopen.bmj.com/site/about/guidelines.xhtml

sample materials to study biobank was signed. The Research biobank has been approved by the Danish Data Protection Agency.

A manuscript with the results of the primary study will be submitted for publication to an international, peer-reviewed journal, regardless of whether results are positive, negative or inconclusive in relation to the study hypothesis. Authorship eligibility will be based on the recommendations from the International Committee of Medical Journal Editors (ICMJE). Upon completion of the trial, and after publication of the primary manuscript, data requests can be submitted to the principal investigator at the Department of Nutrition, Exercise and Sports (NEXS) at the University of Copenhagen, Denmark.

Perspective

The results from this study will serve as proof of concept for the efficacy of using metabolic and genetic biomarkers to provide personalised diet treatments for reducing body fat mass and subsequently for improving health outcomes, such as metabolic and inflammatory markers, in high-risk individuals. Moreover, the findings will inform recommendations regarding the efficacy of using a web-based decision support system for personalising dietary plans to support and maintain health-promoting behaviours.

For peer review only

For peer review only - http://bmjopen.bmj.com/site/about/guidelines.xhtml

Contributors

The overall framework of the EU-project PREVENTOMICS was initiated by BG, AC and JMDB. The overall design of the present Danish study involved all co-authors. Detailed planning, implementation and daily management of the project was carried out by MAA, KP, FM and MFH. MAA, FM and MFH drafted the initial manuscript. KP, SMOG, PM, FS, MP, MW, JMDB, BG critically reviewed and edited the manuscript. All authors approved the final version for publication.

Trial sponsor: University of Copenhagen

Contact name: Faidon Magkos

Email: fma@nexs.ku.dk

Address: 1958 Frederiksberg C, Denmark

Funding: This work and the European partners in the PREVENTOMICS project are supported by the European Union's Horizon 2020 research and innovation program under grant agreement No. 818318.

Competing interests: None declared.

Patient consent for publication: Not required.

Provenance and peer review: Not commissioned; externally peer reviewed

Ethics approval: The study is approved by the Scientific Ethics Committee Region H in Denmark with journal number H-20029882, and registered at Clinicaltrials.gov with: NCT04590989.

Figure 1: Study design and timeline. *The results for metabolome and genotype analyses are integrated into the PREVENTOMICS platform. SF: Simple feast.

Figure 2: Schematic diagram of the intervention. CARB: carbohydrate cluster, LIPID: lipid cluster, INFL: inflammation cluster, OXIS: oxidative stress cluster, MB: microbiota cluster.

For peer review only

References:

1. Ohlhorst SD, Russell R, Bier D, et al. Nutrition research to affect food and a healthy lifespan. Adv Nutr 2013;4(5):579-84.

2. Singhal A. The global epidemic of noncommunicable disease: the role of early-life factors. Nestle Nutr Inst Workshop Ser 2014;78:123-32.

3. Hruby A, Hu FB. The Epidemiology of Obesity: A Big Picture. Pharmacoeconomics 2015;33(7):673-89.

4. Nyberg ST, Batty GD, Pentti J, et al. Obesity and loss of disease-free years owing to major non-communicable diseases: a multicohort study. Lancet Public Health 2018;3(10):e490-e97.

5. Sacks FM, Bray GA, Carey VJ, et al. Comparison of weight-loss diets with different compositions of fat, protein, and carbohydrates. N Engl J Med 2009;360(9):859-73.

6. Shai I, Schwarzfuchs D, Henkin Y, et al. Weight loss with a low-carbohydrate, Mediterranean, or low-fat diet. N Engl J Med 2008;359(3):229-41.

7. Stanton MV, Robinson JL, Kirkpatrick SM, et al. DIETFITS study (diet intervention examining the factors interacting with treatment success) - Study design and methods. Contemp Clin Trials 2017;53:151-61.

8. Hjorth MF, Corella D, Astrup A, et al. High fat diets for weight loss among subjects with elevated fasting glucose levels: The PREDIMED study. Obesity Medicine 2020;18:100210.

9. Aleksandrova K, Egea Rodrigues C, Floegel A, et al. Omics Biomarkers in Obesity: Novel Etiological Insights and Targets for Precision Prevention. Curr Obes Rep 2020;9(3):219-30.

10. Verma M, Hontecillas R, Tubau-Juni N, et al. Challenges in Personalised Nutrition and Health. Front Nutr 2018;5:117.

11. Odriozola L, Corrales FJ. Discovery of nutritional biomarkers: future directions based on omics technologies. Int J Food Sci Nutr 2015;66 Suppl 1:S31-40.

12. Chan AW, Tetzlaff JM, Altman DG, et al. SPIRIT 2013 statement: defining standard protocol items for clinical trials. Ann Intern Med 2013;158(3):200-7.

13. Aulchenko YS, Ripatti S, Lindqvist I, et al. Loci influencing lipid levels and coronary heart disease risk in 16 European population cohorts. Nat Genet 2009;41(1):47-55.

14. Dastani Z, Hivert MF, Timpson N, et al. Novel loci for adiponectin levels and their influence on type 2 diabetes and metabolic traits: a multi-ethnic meta-analysis of 45,891 individuals. PLoS Genet 2012;8(3):e1002607.

15. Draisma HHM, Pool R, Kobl M, et al. Genome-wide association study identifies novel genetic variants contributing to variation in blood metabolite levels. Nat Commun 2015;6:7208.

For peer review only - http://bmjopen.bmj.com/site/about/guidelines.xhtml

16. Guan W, Steffen BT, Lemaitre RN, et al. Genome-wide association study of plasma N6 polyunsaturated fatty acids within the cohorts for heart and aging research in genomic epidemiology consortium. Circ Cardiovasc Genet 2014;7(3):321-31.
17. Hu Y, Liu W, Huang R, et al. A systematic review and meta-analysis of the relationship between lipoprotein lipase Asn291Ser variant and diseases. J Lipid Res 2006;47(9):1908-14.
18. Kathiresan S, Willer CJ, Peloso GM, et al. Common variants at 30 loci contribute to polygenic dyslipidemia. Nat Genet 2009;41(1):56-65.
19. Kilpeläinen TO, Carli JF, Skowronski AA, et al. Genome-wide meta-analysis uncovers novel loci influencing circulating leptin levels. Nat Commun 2016;7:10494.
20. Tintle NL, Pottala JV, Lacey S, et al. A genome-wide association study of saturated, mono- and polyunsaturated red blood cell fatty acids in the Framingham Heart Offspring Study. Prostaglandins Leukot Essent Fatty Acids 2015;94:65-72.
21. Dupuis J, Langenberg C, Prokopenko I, et al. New genetic loci implicated in fasting glucose homeostasis and their impact on type 2 diabetes risk. Nat Genet 2010;42(2):105-16.
22. Mahajan A, Sim X, Ng HJ, et al. Identification and functional characterization of G6PC2 coding variants influencing glycemic traits define an effector transcript at the G6PC2-ABCB11 locus. PLoS Genet 2015;11(1):e1004876.
23. Manning AK, Hivert MF, Scott RA, et al. A genome-wide approach accounting for body mass index identifies genetic variants influencing fasting glycemic traits and insulin resistance. Nat Genet 2012;44(6):659-69.
24. Shin SY, Fauman EB, Petersen AK, et al. An atlas of genetic influences on human blood metabolites. Nat Genet 2014;46(6):543-50.
25. Ahola-Olli AV, Würtz P, Havulinna AS, et al. Genome-wide Association Study Identifies 27 Loci Influencing Concentrations of Circulating Cytokines and Growth Factors. Am J Hum Genet 2017;100(1):40-50.
26. Ligthart S, Vaez A, Võsa U, et al. Genome Analyses of >200,000 Individuals Identify 58 Loci for Chronic Inflammation and Highlight Pathways that Link Inflammation and Complex Disorders. Am J Hum Genet 2018;103(5):691-706.
27. Ljungman P, Bellander T, Nyberg F, et al. DNA variants, plasma levels and variability of interleukin-6 in myocardial infarction survivors: results from the AIRGENE study. Thromb Res 2009;124(1):57-64.
28. Qi L, van Dam RM, Meigs JB, et al. Genetic variation in IL6 gene and type 2 diabetes: tagging-SNP haplotype analysis in large-scale case-control study and meta-analysis. Hum Mol Genet 2006;15(11):1914-20.
29. Galmés S, Cifre M, Palou A, et al. A Genetic Score of Predisposition to Low-Grade Inflammation Associated with Obesity May Contribute to Discern Population at Risk for Metabolic Syndrome. Nutrients 2019;11(2)

For peer review only - http://bmjopen.bmj.com/site/about/guidelines.xhtml

30. Jerotic D, Matic M, Suvakov S, et al. Association of Nrf2, SOD2 and GPX1 Polymorphisms with Biomarkers of Oxidative Distress and Survival in End-Stage Renal Disease Patients. Toxins (Basel) 2019;11(7)
31. van Meurs JB, Pare G, Schwartz SM, et al. Common genetic loci influencing plasma homocysteine concentrations and their effect on risk of coronary artery disease. Am J Clin Nutr 2013;98(3):668-76.
32. Jia Q, Han Y, Huang P, et al. Genetic Determinants of Circulating Glycine Levels and Risk of Coronary Artery Disease. J Am Heart Assoc 2019;8(10):e011922.
33. Wittemans LBL, Lotta LA, Oliver-Williams C, et al. Assessing the causal association of glycine with risk of cardio-metabolic diseases. Nat Commun 2019;10(1):1060.
34. Hernandez-Baixauli J, Quesada-Vázquez S, Mariné-Casadó R, et al. Detection of Early Disease Risk Factors Associated with Metabolic Syndrome: A New Era with the NMR Metabolomics Assessment. Nutrients 2020;12(3)
35. Nordic Nutrition Recommendations 2012 : Integrating nutrition and physical activity. 5 ed. Copenhagen: Nordisk Ministerråd 2014.
36. Salmon J. Excerpts from Dietary Reference Values for Food Energy and Nutrients for the United Kingdom: introduction to the Guide and Summary Tables. Nutr Rev 1992;50(3):90-3.
37. Habibović M, Broers E, Piera-Jimenez J, et al. Enhancing Lifestyle Change in Cardiac Patients Through the Do CHANGE System ("Do Cardiac Health: Advanced New Generation Ecosystem"): Randomized Controlled Trial Protocol. JMIR Res Protoc 2018;7(2):e40.
38. Bingham SA, Gill C, Welch A, et al. Validation of dietary assessment methods in the UK arm of EPIC using weighed records, and 24-hour urinary nitrogen and potassium and serum vitamin C and carotenoids as biomarkers. Int J Epidemiol 1997;26 Suppl 1:S137-51.
39. Stunkard AJ, Messick S. The three-factor eating questionnaire to measure dietary restraint, disinhibition and hunger. J Psychosom Res 1985;29(1):71-83.
40. Cohen S, Kamarck T, Mermelstein R. A global measure of perceived stress. J Health Soc Behav 1983;24(4):385-96.
41. EuroQol--a new facility for the measurement of health-related quality of life. Health Policy 1990;16(3):199-208.
42. Niero M, Martin M, Finger T, et al. A new approach to multicultural item generation in the development of two obesity-specific measures: the Obesity and Weight Loss Quality of Life (OWLQOL) questionnaire and the Weight-Related Symptom Measure (WRSM). Clin Ther 2002;24(4):690-700.
43. James BL, Loken E, Roe LS, et al. Validation of the Diet Satisfaction Questionnaire: a new measure of satisfaction with diets for weight management. Obes Sci Pract 2018;4(6):506-14.

For peer review only

For peer review only - http://bmjopen.bmj.com/site/about/guidelines.xhtml

44. Poulsen SK, Due A, Jordy AB, et al. Health effect of the New Nordic Diet in adults with increased waist circumference: a 6-mo randomized controlled trial. Am J Clin Nutr 2014;99(1):35-45.

For peer review only

For peer review only - http://bmjopen.bmj.com/site/about/guidelines.xhtml

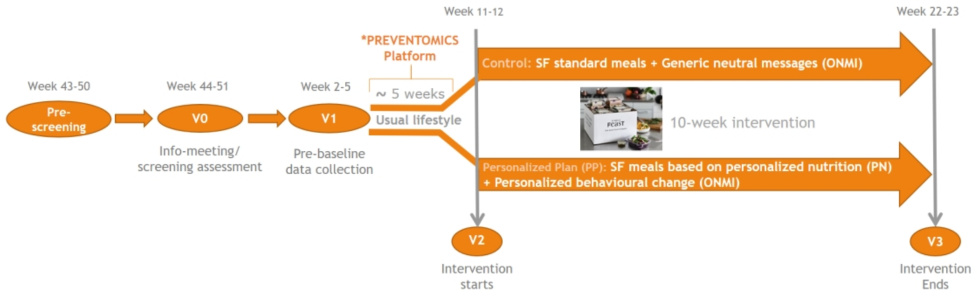

Figure 1: Study design and timeline. *The analysis results on subjects' metabolome and genotypes will be integrated into PREVENTOMICS platform. This takes about 5 weeks from the date of sending samples from the University of Copenhagen to the assigned partners (Eurecat, Alimentomica). SF, Simple Feast.

819x282mm (57 x 57 DPI)

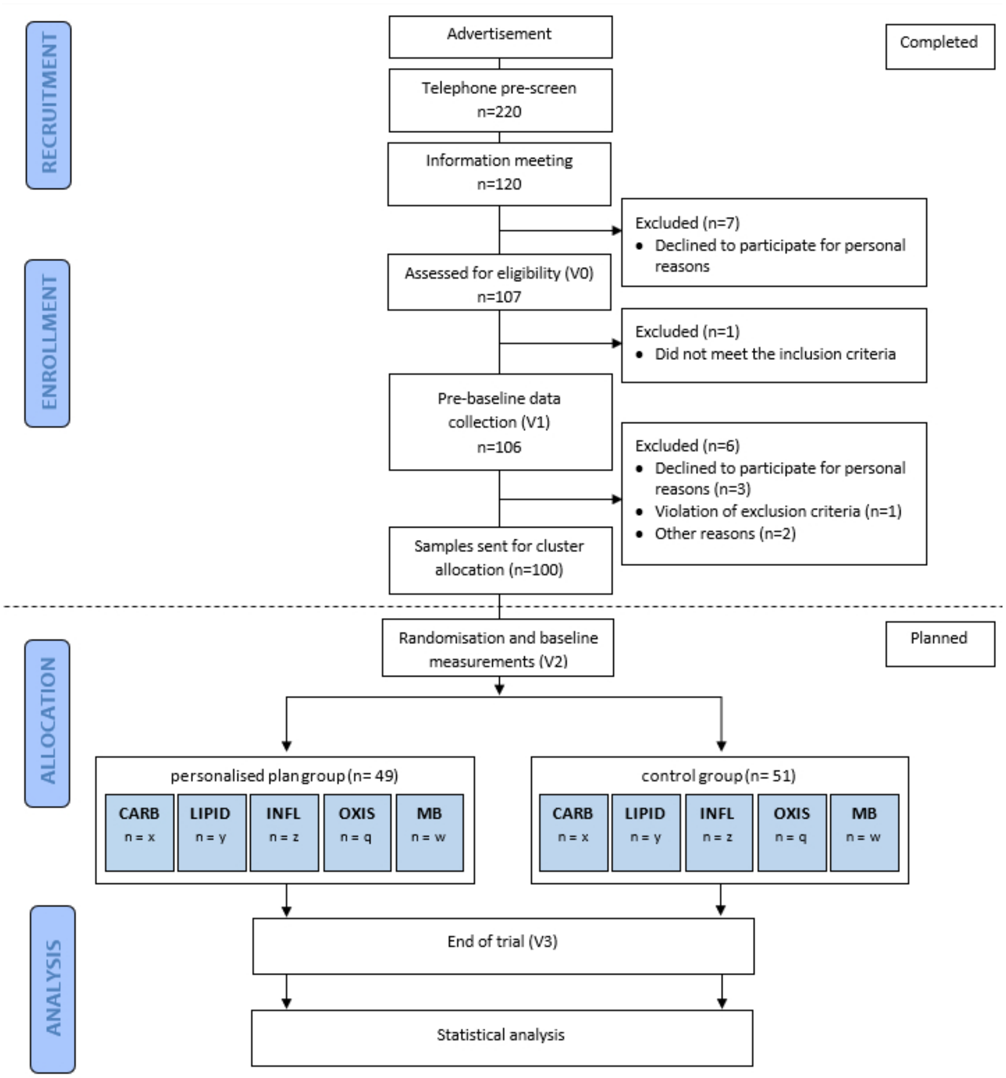

Figure 2: Schematic diagram of the intervention. CARB, carbohydrate cluster; LIPID, lipid cluster; INFL, inflammation cluster; OXIS, oxidative stress cluster; MB, microbiota cluster.

388x419mm (47 x 47 DPI)

SUPPLEMENTARY ONLINE MATERIAL 1

Table 1: Biomarkers included in the carbohydrate cluster

**(1) Carbohydrates cluster**		
**Biomarker**	**Bio-fluid**	**Reference**
**Glucose**	Plasma	Cosentino et al., 2020[1]
**HOMA-IR**	-	Bloomgarden, 2003[2]<br>Govers et al., 2015[3]<br>Shashaj et al., 2016[4]
**Insulin**	Serum	Aleksandrova et al., 2018[5]<br>Bloomgarden, 2003[2]<br>Govers et al., 2015[3]
**Leptin**	Serum	Chen et al., 2014[6]<br>Finucane et al., 2019[7]<br>López-Jaramillo et al., 2014[8]
**Adiponectin**	Serum	Dastani et al., 2012[9]<br>Li et al., 2009[10]<br>Liu et al., 2016[11]<br>Wang et al., 2018[12]
**Lactate**	Serum	Berhane et al., 2015[13]<br>Choi et al., 2002[14]<br>Lovejoy et al., 1992[15]<br>Shantha et al., 2013[16]
**Glutamate**	Serum	Martin and Price, 2018[17]<br>Ottosson et al., 2018[18]
**Uric acid**	Serum	Darmawan et al., 2018[19]<br>Fabbrini et al., 2014[20]<br>Johnson et al., 2013[21]<br>van der Schaft et al., 2017[22]
**Propionylcarnitine**	Plasma	Mai et al., 2013[23]<br>Mihalik et al., 2010[24]<br>Zhang et al., 2014[25]
**BCAA (Valine, Leucine, Isoleucine)**	Serum	Chen et al., 2016[26]<br>Katagiri et al., 2018[27]<br>Lotta et al., 2016[28]<br>Newgard et al., 2009[29]<br>Okekunle et al., 2019[30]
**Phenylalanine**	Serum	Chen et al., 2019[31]<br>Suzuki et al., 2019[32]<br>Wang et al., 2011[33]
**Tyrosine**	Serum	Chen et al., 2019[31]<br>Hellmuth et al., 2016[34]<br>Wang et al., 2011[33]
**Glutamine**	Serum	Chen et al., 2019[31]<br>Guasch-Ferré et al., 2016[35]<br>Liu et al., 2019[36]<br>Rhee et al., 2018[37]

Abbreviations: HOMA-IR, homeostatic model assessment of insulin resistance; BCAA, branched chain amino acids.

For peer review only

For peer review only - http://bmjopen.bmj.com/site/about/guidelines.xhtml

Table 2: Biomarkers included in the lipid cluster

**(2) Lipid cluster**		
**Biomarker**	**Bio-fluid**	**Reference**
**LDL-cholesterol**	Plasma	Grundy et al., 2019[38]
**HDL-cholesterol**	Plasma	Grundy et al., 2019[38]
**Triglycerides**	Plasma	Grundy et al., 2019[38]
**Total cholesterol**	Plasma	Grundy et al., 2019[38]
**PUFAs (total)**	Serum	Koga et al., 2019[39]
**LPCs (total)**	Plasma	Law et al., 2019[40]
**Linoleic acid C18:2**	Serum	Koga et al., 2019[39]
**Oleic acid C18:1**	Serum	Griel and Kris-Etherton, 2006[41]<br>Yamagishi et al., 2013[42]
**Leptin**	Serum	Mantzoros and Flier, 2000[43]
**Adiponectin**	Serum	Abdella and Mojiminiyi, 2018[44]<br>Liu et al., 2018[45]
**SFAs**	Serum	Liu et al., 2019[46]
**3-hydroxybutyrate**	Serum	Margolis and O'Fallon, 2020[47]
**MUFAs (total)**	Serum	Griel and Kris-Etherton, 2006[41]<br>Yamagishi et al., 2013[42]
**Propionylcarnitine**	Plasma	Ottosson et al., 2018[18]
**DHA C20:3**	Serum	Koga et al., 2019[39]
**Choline**	Serum	Ottosson et al., 2018[18]

Abbreviations: LDL, low-density lipoprotein; HDL, high-density lipoprotein; PUFAs, poly unsaturated fatty acids; LPC, lysophosphatidylcholine; SFAs, saturated fatty acids; MUFAs, monounsaturated fatty acids; DHA, docosahexaenoic acid.

For peer review only

For peer review only - http://bmjopen.bmj.com/site/about/guidelines.xhtml

Table 3: Biomarkers included in the inflammation cluster

**(3) Inflammation cluster**		
**Biomarker**	**Bio-fluid**	**Reference**
**CRP**	Serum	Amsterdam, 2003[48]<br>Knight, 2015[49]<br>Liu et al., 2016[11]<br>Musunuru et al., 2008[50]<br>Wang et el., 2013[51]
**IL-6**	Plasma	Liu et al., 2016[11]<br>Wang et al., 2013[51]
**N-acetylglycoproteins**	Serum	Gruppen et al., 2019[52]<br>Ritchie et al., 2015[53]
**TNFα**	Plasma	Liu et al., 2016[11]
**MCP-1**	Plasma	Georgakis et al., 2019[54]
**IL-10**	Plasma	Charles et al., 2011[55]<br>Leon-Cabrera et al., 2015[56]<br>Meng et al., 2019[57]
**sICAM1**	Plasma	El Amine et al., 2010[58]<br>Luc et al., 2003[59]<br>Straczkowski et al., 2002[60]
**LBP**	Plasma	Moreno-Navarrete et al., 2012[61]
**sCD14**	Plasma	de Courten et al., 2016[62]
**LPCs (total)**	Plasma	Iwase et al., 2008[63]
**Linoleic acid C18:2**	Serum	Steffen et al., 2012[64]<br>Yli-Jama et al., 2002[65]
**DHA C20:3**	Serum	Steffen et al., 2012[64]<br>Yli-Jama et al., 2002[65]

Abbreviations: CRP, C-reactive protein; IL-6, interleukin-6; IL-10, interleukin-10; TNFα, tumor necrosis factor alpha; MCP-1, monocyte chemoattractant protein-1; sICAM-1, soluble intercellular adhesion molecule-1; LBP, lipopolysaccharide binding protein; sCD14, soluble CD14; LPC, lysophosphatidylcholines; DHA: docosahexaenoic acid.

For peer review only

For peer review only - http://bmjopen.bmj.com/site/about/guidelines.xhtml

Table 4: Biomarkers included in the oxidative stress cluster

(4) Oxidative stress cluster		
**Biomarker**	**Bio-fluid**	**Reference**
**8-iso-PGF2α**	Urine	Davies and Roberts, 2011[66]<br>Kim et al., 2012[67]<br>Milne et al., 2015[68]<br>van't Erve, 2018[69]
**8-OHdG**	Urine	Di Minno et al., 2016[70]<br>Kroese and Scheffe, 2014[71]
**LDLox**	Plasma	Barbosa et al., 2011[72]<br>Gao et al., 2017[73]
**Uric acid**	Serum	Darmawan et al., 2018[19]<br>Fabbrini et al., 2014[20]<br>van der Schaft et al., 2017[22]
**Allantoin**	Urine	Il'yasova et al., 2012[74]
**Betaine**	Urine	Svingen et al., 2016[75]<br>Walford et al., 2016[76]
**Pseudouridine**	Urine	Topp et al., 2008[77]
**Dimethylglycine**	Urine	Svingen et al., 2016[75]
**Glycine**	Serum	Sekhar et al., 2011[78]
**Methionine**	Serum	Grizales et al., 2018[79]

Abbreviations: 8-iso-PGF2α, 8-iso-prostaglandin F2 alpha; 8-OHdG, 8-hydroxydeoxyguanosine; LDLox, oxidized low density lipoprotein.

For peer review only

For peer review only - http://bmjopen.bmj.com/site/about/guidelines.xhtml

Table 5: Biomarkers included in the microbiota cluster

**(5) Microbiota cluster**		
**Biomarker**	**Bio-fluid**	**Reference**
**TMAO**	Serum	Bain et al., 2006[80]<br>Chen et al., 2016[81]<br>Ge et al., 2020[82]<br>Heianza et al., 2017[83]<br>Schiattarella et al., 2017[84]<br>Yang et el., 2019[85]<br>Yao et al., 2020[86]
**TMA**	Urine	Aragonès et al., 2019[87]<br>Bouatra et al., 2013[88]<br>Chen et al., 2016[81]<br>Palau-Rodriguez et al., 2015[89]<br>Yang et el., 2019[85]
**DMA**	Urine	Bouatra et al., 2013[88]<br>Palau-Rodriguez et al., 2015[89]
**Betaine**	Urine	Chen et al., 2016[81]<br>Palau-Rodriguez et al., 2015[89]<br>Yang et el., 2019[85]
**Choline**	Serum	Aragonès et al., 2019[87]<br>Chen et al., 2016[81]<br>Palau-Rodriguez et al., 2015[89]<br>Yang et el., 2019[85]
**Dimethylglycine**	Urine	Palau-Rodriguez et al., 2015[89]
**Succinate**	Plasma	Psychogios et al., 2011[90]<br>Serena et al., 2018[91]
**Lactate**	Serum	Psychogios et al., 2011[90]<br>Vojinovic et al., 2019[92]
**LBP**	Plasma	Awoyemi et al., 2018[93]<br>Barengolts et al., 2019[94]<br>Bouatra et al., 2013[88]<br>Citronberg et al., 2018[95]
**Acetate**	Urine	Liu et al., 2014[96]

Abbreviations: TMAO, trimethylamine N-oxide; TMA, trimethylamine; DMA, dimethylamine; LBP, lipopolysaccharide binding protein.

For peer review only

For peer review only - http://bmjopen.bmj.com/site/about/guidelines.xhtml

References:

1. Cosentino F, Grant PJ, Aboyans V, et al. 2019 ESC Guidelines on diabetes, pre-diabetes, and cardiovascular diseases developed in collaboration with the EASD. *Eur Heart J* 2020;41(2):255-323.
2. Bloomgarden ZT. American Association of Clinical Endocrinologists (AACE) consensus conference on the insulin resistance syndrome: 25-26 August 2002, Washington, DC. *Diabetes Care* 2003;26(4):1297-303.
3. Govers E, Slof E, Verkoelen H, et al. Guideline for the Management of Insulin Resistance. *Int J Endocrinol Metab Disord* 2015;1(4).
4. Shashaj B, Luciano R, Contoli B, et al. Reference ranges of HOMA-IR in normal-weight and obese young Caucasians. *Acta Diabetol* 2016;53(2):251-60.
5. Aleksandrova K, Mozaffarian D, Pischon T. Addressing the Perfect Storm: Biomarkers in Obesity and Pathophysiology of Cardiometabolic Risk. *Clin Chem* 2018;64(1):142-53.
6. Chen GC, Qin LQ, Ye JK. Leptin levels and risk of type 2 diabetes: gender-specific meta-analysis. *Obes Rev* 2014;15(2):134-42.
7. Finucane FM, Luan J, Wareham NJ, et al. Correlation of the leptin:adiponectin ratio with measures of insulin resistance in non-diabetic individuals. *Diabetologia* 2009;52(11):2345-49.
8. López-Jaramillo P, Gómez-Arbeláez D, López-López J, et al. The role of leptin/adiponectin ratio in metabolic syndrome and diabetes. *Horm Mol Biol Clin Investig* 2014;18(1):37-45.
9. Dastani Z, Hivert MF, Timpson N, et al. Novel loci for adiponectin levels and their influence on type 2 diabetes and metabolic traits: a multi-ethnic meta-analysis of 45,891 individuals. *PLoS Genet* 2012;8(3):e1002607.
10. Li S, Shin HJ, Ding EL, et al. Adiponectin levels and risk of type 2 diabetes: a systematic review and meta-analysis. *Jama* 2009;302(2):179-88.
11. Liu C, Feng X, Li Q, et al. Adiponectin, TNF-α and inflammatory cytokines and risk of type 2 diabetes: A systematic review and meta-analysis. *Cytokine* 2016;86:100-09.
12. Wang Y, Meng RW, Kunutsor SK, et al. Plasma adiponectin levels and type 2 diabetes risk: a nested case-control study in a Chinese population and an updated meta-analysis. *Sci Rep* 2018;8(1):406.
13. Berhane F, Fite A, Daboul N, et al. Plasma Lactate Levels Increase during Hyperinsulinemic Euglycemic Clamp and Oral Glucose Tolerance Test. *J Diabetes Res* 2015;2015:102054.
14. Choi CS, Kim YB, Lee FN, et al. Lactate induces insulin resistance in skeletal muscle by suppressing glycolysis and impairing insulin signaling. *Am J Physiol Endocrinol Metab* 2002;283(2):E233-40.
15. Lovejoy J, Newby FD, Gebhart SSP, et al. Insulin resistance in obesity is associated with elevated basal lactate levels and diminished lactate appearance following intravenous glucose and insulin. *Metabolism* 1992;41(1):22-27.
16. Shantha GP, Wasserman B, Astor BC, et al. Association of blood lactate with carotid atherosclerosis: the Atherosclerosis Risk in Communities (ARIC) Carotid MRI Study. *Atherosclerosis* 2013;228(1):249-55.
17. Martin AJ, Price CI. A Systematic Review and Meta-Analysis of Molecular Biomarkers Associated with Early Neurological Deterioration Following Acute Stroke. *Cerebrovasc Dis* 2018;46(5-6):230-41.
18. Ottosson F, Brunkwall L, Ericson U, et al. Connection Between BMI-Related Plasma Metabolite Profile and Gut Microbiota. *J Clin Endocrinol Metab* 2018;103(4):1491-501.
19. Darmawan G, Hamijoyo L, Hasan I. Association between Serum Uric Acid and Non-Alcoholic Fatty Liver Disease: A Meta-Analysis. *Acta Med Indones* 2017;49(2):136-47.
20. Fabbrini E, Serafini M, Colic Baric I, et al. Effect of plasma uric acid on antioxidant capacity, oxidative stress, and insulin sensitivity in obese subjects. *Diabetes* 2014;63(3):976-81.
21. Johnson RJ, Nakagawa T, Sanchez-Lozada LG, et al. Sugar, uric acid, and the etiology of diabetes and obesity. *Diabetes* 2013;62(10):3307-15.
22. van der Schaft N, Brahimaj A, Wen KX, et al. The association between serum uric acid and the incidence of prediabetes and type 2 diabetes mellitus: The Rotterdam Study. *PLoS One* 2017;12(6):e0179482.
23. Mai M, Tönjes A, Kovacs P, et al. Serum levels of acylcarnitines are altered in prediabetic conditions. *PLoS One* 2013;8(12):e82459.
24. Mihalik SJ, Goodpaster BH, Kelley DE, et al. Increased levels of plasma acylcarnitines in obesity and type 2 diabetes and identification of a marker of glucolipotoxicity. *Obesity (Silver Spring)* 2010;18(9):1695-700.
25. Zhang X, Zhang C, Chen L, et al. Human serum acylcarnitine profiles in different glucose tolerance states. *Diabetes Res Clin Pract* 2014;104(3):376-82.
26. Chen T, Ni Y, Ma X, et al. Branched-chain and aromatic amino acid profiles and diabetes risk in Chinese populations. *Sci Rep* 2016;6:20594.
27. Katagiri R, Goto A, Budhathoki S, et al. Association between plasma concentrations of branched-chain amino acids and adipokines in Japanese adults without diabetes. *Sci Rep* 2018;8(1):1043.

For peer review only

For peer review only - http://bmjopen.bmj.com/site/about/guidelines.xhtml

28. Lotta LA, Scott RA, Sharp SJ, et al. Genetic Predisposition to an Impaired Metabolism of the Branched-Chain Amino Acids and Risk of Type 2 Diabetes: A Mendelian Randomisation Analysis. *PLoS Med* 2016;13(11):e1002179.
29. Newgard CB, An J, Bain JR, et al. A branched-chain amino acid-related metabolic signature that differentiates obese and lean humans and contributes to insulin resistance. *Cell Metab* 2009;9(4):311-26.
30. Okekunle AP, Zhang M, Wang Z, et al. Dietary branched-chain amino acids intake exhibited a different relationship with type 2 diabetes and obesity risk: a meta-analysis. *Acta Diabetol* 2019;56(2):187-95.
31. Chen S, Akter S, Kuwahara K, et al. Serum amino acid profiles and risk of type 2 diabetes among Japanese adults in the Hitachi Health Study. *Sci Rep* 2019;9(1):7010.
32. Suzuki Y, Kido J, Matsumoto S, et al. Associations among amino acid, lipid, and glucose metabolic profiles in childhood obesity. *BMC Pediatr* 2019;19(1):273.
33. Wang TJ, Larson MG, Vasan RS, et al. Metabolite profiles and the risk of developing diabetes. *Nat Med* 2011;17(4):448-53.
34. Hellmuth C, Kirchberg FF, Lass N, et al. Tyrosine Is Associated with Insulin Resistance in Longitudinal Metabolomic Profiling of Obese Children. *J Diabetes Res* 2016;2016:2108909.
35. Guasch-Ferré M, Hruby A, Toledo E, et al. Metabolomics in Prediabetes and Diabetes: A Systematic Review and Meta-analysis. *Diabetes Care* 2016;39(5):833-46.
36. Liu X, Zheng Y, Guasch-Ferré M, et al. High plasma glutamate and low glutamine-to-glutamate ratio are associated with type 2 diabetes: Case-cohort study within the PREDIMED trial. *Nutr Metab Cardiovasc Dis* 2019;29(10):1040-49.
37. Rhee SY, Jung ES, Park HM, et al. Plasma glutamine and glutamic acid are potential biomarkers for predicting diabetic retinopathy. *Metabolomics* 2018;14(7):89.
38. Grundy SM, Stone NJ, Bailey AL, et al. 2018 AHA/ACC/AACVPR/AAPA/ABC/ACPM/ADA/AGS/APhA/ASPC/NLA/PCNA Guideline on the Management of Blood Cholesterol: A Report of the American College of Cardiology/American Heart Association Task Force on Clinical Practice Guidelines. *J Am Coll Cardiol* 2019;73(24):e285-e350.
39. Koga N, Ogura J, Yoshida F, et al. Altered polyunsaturated fatty acid levels in relation to proinflammatory cytokines, fatty acid desaturase genotype, and diet in bipolar disorder. *Transl Psychiatry* 2019;9(1):208.
40. Law SH, Chan ML, Marathe GK, et al. An Updated Review of Lysophosphatidylcholine Metabolism in Human Diseases. *Int J Mol Sci* 2019;20(5).
41. Griel AE, Kris-Etherton PM. Tree nuts and the lipid profile: a review of clinical studies. *Br J Nutr* 2006;96 Suppl 2:S68-78.
42. Yamagishi K, Folsom AR, Steffen LM. Plasma fatty acid composition and incident ischemic stroke in middle-aged adults: the Atherosclerosis Risk in Communities (ARIC) Study. *Cerebrovasc Dis* 2013;36(1):38-46.
43. Mantzoros CS, Flier JS. Editorial: leptin as a therapeutic agent--trials and tribulations. *J Clin Endocrinol Metab* 2000;85(11):4000-2.
44. Abdella NA, Mojiminiyi OA. Clinical Applications of Adiponectin Measurements in Type 2 Diabetes Mellitus: Screening, Diagnosis, and Marker of Diabetes Control. *Dis Markers* 2018;2018:5187940.
45. Liu Z, Liang S, Que S, et al. Meta-Analysis of Adiponectin as a Biomarker for the Detection of Metabolic Syndrome. *Front Physiol* 2018;9:1238.
46. Liu Q, Matthan NR, Manson JE, et al. Plasma Phospholipid Fatty Acids and Coronary Heart Disease Risk: A Matched Case-Control Study within the Women's Health Initiative Observational Study. *Nutrients* 2019;11(7).
47. Margolis LM, O'Fallon KS. Utility of Ketone Supplementation to Enhance Physical Performance: A Systematic Review. *Adv Nutr* 2020;11(2):412-19.
48. Amsterdam EA. C-reactive protein: a guideline for its application. *Prev Cardiol* 2003;6(2):70.
49. Knight ML. The Application of High-Sensitivity C-Reactive Protein in Clinical Practice: A 2015 Update. *US Pharm* 2015;40(2):50-53.
50. Musunuru K, Kral BG, Blumenthal RS, et al. The use of high-sensitivity assays for C-reactive protein in clinical practice. *Nat Clin Pract Cardiovasc Med* 2008;5(10):621-35.
51. Wang X, Bao W, Liu J, et al. Inflammatory markers and risk of type 2 diabetes: a systematic review and meta-analysis. *Diabetes Care* 2013;36(1):166-75.
52. Gruppen EG, Kunutsor SK, Kieneker LM, et al. GlycA, a novel pro-inflammatory glycoprotein biomarker is associated with mortality: results from the PREVEND study and meta-analysis. *J Intern Med* 2019;286(5):596-609.
53. Ritchie SC, Würtz P, Nath AP, et al. The Biomarker GlycA Is Associated with Chronic Inflammation and Predicts Long-Term Risk of Severe Infection. *Cell Syst* 2015;1(4):293-301.

For peer review only - http://bmjopen.bmj.com/site/about/guidelines.xhtml

54. Georgakis MK, Malik R, Björkbacka H, et al. Circulating Monocyte Chemoattractant Protein-1 and Risk of Stroke: Meta-Analysis of Population-Based Studies Involving 17 180 Individuals. *Circ Res* 2019;125(8):773-82.
55. Charles BA, Doumatey A, Huang H, et al. The roles of IL-6, IL-10, and IL-1RA in obesity and insulin resistance in African-Americans. *J Clin Endocrinol Metab* 2011;96(12):E2018-22.
56. Leon-Cabrera S, Arana-Lechuga Y, Esqueda-León E, et al. Reduced systemic levels of IL-10 are associated with the severity of obstructive sleep apnea and insulin resistance in morbidly obese humans. *Mediators Inflamm* 2015;2015:493409.
57. Meng D, Liang L, Guo X. Serum interleukin-10 level in patients with inflammatory bowel disease: A meta-analysis. *Eur J Inflamm* 2019;17:1-7.
58. El Amine M, Sohawon S, Lagneau L, et al. Plasma levels of ICAM-1 and circulating endothelial cells are elevated in unstable types 1 and 2 diabetes. *Endocr Regul* 2010;44(1):17-24.
59. Luc G, Arveiler D, Evans A, et al. Circulating soluble adhesion molecules ICAM-1 and VCAM-1 and incident coronary heart disease: the PRIME Study. *Atherosclerosis* 2003;170(1):169-76.
60. Straczkowski M, Lewczuk P, Dzienis-Straczkowska S, et al. Elevated soluble intercellular adhesion molecule-1 levels in obesity: relationship to insulin resistance and tumor necrosis factor-alpha system activity. *Metabolism* 2002;51(1):75-8.
61. Moreno-Navarrete JM, Ortega F, Serino M, et al. Circulating lipopolysaccharide-binding protein (LBP) as a marker of obesity-related insulin resistance. *Int J Obes (Lond)* 2012;36(11):1442-9.
62. de Courten B, Moreno-Navarrete JM, Lyons J, et al. Contrasting association of circulating sCD14 with insulin sensitivity in non-obese and morbidly obese subjects. *Mol Nutr Food Res* 2016;60(1):103-9.
63. Iwase M, Sonoki K, Sasaki N, et al. Lysophosphatidylcholine contents in plasma LDL in patients with type 2 diabetes mellitus: relation with lipoprotein-associated phospholipase A2 and effects of simvastatin treatment. *Atherosclerosis* 2008;196(2):931-6.
64. Steffen BT, Steffen LM, Tracy R, et al. Obesity modifies the association between plasma phospholipid polyunsaturated fatty acids and markers of inflammation: the Multi-Ethnic Study of Atherosclerosis. *Int J Obes (Lond)* 2012;36(6):797-804.
65. Yli-Jama P, Seljeflot I, Meyer HE, et al. Serum non-esterified very long-chain PUFA are associated with markers of endothelial dysfunction. *Atherosclerosis* 2002;164(2):275-81.
66. Davies SS, Roberts LJ, 2nd. F2-isoprostanes as an indicator and risk factor for coronary heart disease. *Free Radic Biol Med* 2011;50(5):559-66.
67. Kim JY, Lee JW, Youn YJ, et al. Urinary levels of 8-iso-prostaglandin f2α and 8-hydroxydeoxyguanine as markers of oxidative stress in patients with coronary artery disease. *Korean Circ J* 2012;42(9):614-7.
68. Milne GL, Dai Q, Roberts LJ, 2nd. The isoprostanes--25 years later. *Biochim Biophys Acta* 2015;1851(4):433-45.
69. van 't Erve TJ. Strategies to decrease oxidative stress biomarker levels in human medical conditions: A meta-analysis on 8-iso-prostaglandin F(2α). *Redox Biol* 2018;17:284-96.
70. Di Minno A, Turnu L, Porro B, et al. 8-Hydroxy-2-Deoxyguanosine Levels and Cardiovascular Disease: A Systematic Review and Meta-Analysis of the Literature. *Antioxid Redox Signal* 2016;24(10):548-55.
71. Kroese LJ, Scheffer PG. 8-hydroxy-2'-deoxyguanosine and cardiovascular disease: a systematic review. *Curr Atheroscler Rep* 2014;16(11):452.
72. Barbosa KB, Volp AC, Hermsdorff HH, et al. Relationship of oxidized low density lipoprotein with lipid profile and oxidative stress markers in healthy young adults: a translational study. *Lipids Health Dis* 2011;10:61.
73. Gao S, Zhao D, Wang M, et al. Association Between Circulating Oxidized LDL and Atherosclerotic Cardiovascular Disease: A Meta-analysis of Observational Studies. *Can J Cardiol* 2017;33(12):1624-32.
74. Il'yasova D, Scarbrough P, Spasojevic I. Urinary biomarkers of oxidative status. *Clin Chim Acta* 2012;413(19-20):1446-53.
75. Svingen GF, Schartum-Hansen H, Pedersen ER, et al. Prospective Associations of Systemic and Urinary Choline Metabolites with Incident Type 2 Diabetes. *Clin Chem* 2016;62(5):755-65.
76. Walford GA, Ma Y, Clish C, et al. Metabolite Profiles of Diabetes Incidence and Intervention Response in the Diabetes Prevention Program. *Diabetes* 2016;65(5):1424-33.
77. Topp H, Fusch G, Schöch G, et al. Noninvasive markers of oxidative DNA stress, RNA degradation and protein degradation are differentially correlated with resting metabolic rate and energy intake in children and adolescents. *Pediatr Res* 2008;64(3):246-50.
78. Sekhar RV, McKay SV, Patel SG, et al. Glutathione synthesis is diminished in patients with uncontrolled diabetes and restored by dietary supplementation with cysteine and glycine. *Diabetes Care* 2011;34(1):162-7.
79. Grizales AM, Patti ME, Lin AP, et al. Metabolic Effects of Betaine: A Randomized Clinical Trial of Betaine Supplementation in Prediabetes. *J Clin Endocrinol Metab* 2018;103(8):3038-49.

For peer review only - http://bmjopen.bmj.com/site/about/guidelines.xhtml

80. Bain MA, Faull R, Fornasini G, et al. Accumulation of trimethylamine and trimethylamine-N-oxide in end-stage renal disease patients undergoing haemodialysis. *Nephrol Dial Transplant* 2006;21(5):1300-4.
81. Chen YM, Liu Y, Zhou RF, et al. Associations of gut-flora-dependent metabolite trimethylamine-N-oxide, betaine and choline with non-alcoholic fatty liver disease in adults. *Sci Rep* 2016;6:19076.
82. Ge X, Zheng L, Zhuang R, et al. The Gut Microbial Metabolite Trimethylamine N-Oxide and Hypertension Risk: A Systematic Review and Dose-Response Meta-analysis. *Adv Nutr* 2020;11(1):66-76.
83. Heianza Y, Ma W, Manson JE, et al. Gut Microbiota Metabolites and Risk of Major Adverse Cardiovascular Disease Events and Death: A Systematic Review and Meta-Analysis of Prospective Studies. *J Am Heart Assoc* 2017;6(7)
84. Schiattarella GG, Sannino A, Toscano E, et al. Gut microbe-generated metabolite trimethylamine-N-oxide as cardiovascular risk biomarker: a systematic review and dose-response meta-analysis. *Eur Heart J* 2017;38(39):2948-56.
85. Yang S, Li X, Yang F, et al. Gut Microbiota-Dependent Marker TMAO in Promoting Cardiovascular Disease: Inflammation Mechanism, Clinical Prognostic, and Potential as a Therapeutic Target. *Front Pharmacol* 2019;10:1360.
86. Yao ME, Liao PD, Zhao XJ, et al. Trimethylamine-N-oxide has prognostic value in coronary heart disease: a meta-analysis and dose-response analysis. *BMC Cardiovasc Disord* 2020;20(1):7.
87. Aragonès G, González-García S, Aguilar C, et al. Gut Microbiota-Derived Mediators as Potential Markers in Nonalcoholic Fatty Liver Disease. *Biomed Res Int* 2019;2019:8507583.
88. Bouatra S, Aziat F, Mandal R, et al. The human urine metabolome. *PLoS One* 2013;8(9):e73076.
89. Palau-Rodriguez M, Tulipani S, Isabel Queipo-Ortuño M, et al. Metabolomic insights into the intricate gut microbial-host interaction in the development of obesity and type 2 diabetes. *Front Microbiol* 2015;6:1151.
90. Psychogios N, Hau DD, Peng J, et al. The human serum metabolome. *PLoS One* 2011;6(2):e16957.
91. Serena C, Ceperuelo-Mallafré V, Keiran N, et al. Elevated circulating levels of succinate in human obesity are linked to specific gut microbiota. *Isme j* 2018;12(7):1642-57.
92. Vojinovic D, Radjabzadeh D, Kurilshikov A, et al. Relationship between gut microbiota and circulating metabolites in population-based cohorts. *Nat Commun* 2019;10(1):5813.
93. Awoyemi A, Trøseid M, Arnesen H, et al. Markers of metabolic endotoxemia as related to metabolic syndrome in an elderly male population at high cardiovascular risk: a cross-sectional study. *Diabetol Metab Syndr* 2018;10:59.
94. Barengolts E, Green SJ, Chlipala GE, et al. Predictors of Obesity among Gut Microbiota Biomarkers in African American Men with and without Diabetes. *Microorganisms* 2019;7(9).
95. Citronberg JS, Curtis KR, White E, et al. Association of gut microbial communities with plasma lipopolysaccharide-binding protein (LBP) in premenopausal women. *Isme j* 2018;12(7):1631-41.
96. Liu X, Lu L, Yao P, et al. Lipopolysaccharide binding protein, obesity status and incidence of metabolic syndrome: a prospective study among middle-aged and older Chinese. *Diabetologia* 2014;57(9):1834-41.

For peer review only

For peer review only - http://bmjopen.bmj.com/site/about/guidelines.xhtml

SUPPLEMENTARY ONLINE MATERIAL 2

Table 1: Target daily amounts of macronutrients and functional ingredients in meals provided to the personalised plan and control groups

		Personalised plan cluster				
**Meal: Breakfast + dinner**	**Control**	**Carbohydrate**	**Microbiota**	**Lipid**	**Inflammation**	**Oxidative stress**
Energy, kcal	1200 - 1500	1200 - 1500	1200 - 1500	1200 - 1500	1200 - 1500	1200 - 1500
Protein, %	10 - 14	10 - 14	10 - 14	10 - 14	10 - 14	10 - 14
Carbohydrate, %	45 - 55	45 - 55	45 - 55	45 - 55	45 - 55	45 - 55
Dietary fibre, g	22 - 28	42 – 52*	47 – 52*	22 - 28	22 - 28	22 - 28
Added sugars, g	0	0	0	0	0	0
Fat, %	30 - 40	30 - 40	30 - 40	30 - 40	30 - 40	30 - 40
**Functional ingredient**						
Fructooligosaccharides and/or Inulin		20 g	20 g			
Sunflower oil				20 g		
Turmeric powder					6-8 g	
Oleic acid enriched sunflower oil						20 g

Values are presented in ranges (minimum-maximum) for both genders.
**Including the functional ingredient*

For peer review only

For peer review only - http://bmjopen.bmj.com/site/about/guidelines.xhtml

Table 2: Example of 3-day menus provided to the personalised plan and control groups during the 10-week intervention

				Substituted or added ingredient in each cluster of the personalised plan group				
**Day**	**Breakfast meal**	**Ingredient**	**Control (female/male)**	**Carbohydrate**	**Microbiota**	**Lipid**	**Inflammation**	**Oxidative stress**
**1**	• You Go Ghurt with granola<br>• Kale salad with orange and roasted walnuts<br>• Cucumber and celery with pumpkin seeds juice	Yogurt, You Go Ghurt	160/210 g					
		Roasted walnuts	8/10 g			Not roasted	Not roasted	Not roasted
		Granola	20/25 g	+ 5 g FOS	+ 5 g FOS			
		Orange	1 piece/medium					
		Kale	40 g				100 g coleslaw	150 g red bell pepper
		Cucumber and celery with pumpkin seeds juice	250 mL				+3 g turmeric	
**2**	• Quinoa porridge with baked strawberry compote<br>• Blood orange with pine nuts and chervil<br>• Yogurt smoothie with Mint	Quinoa porridge	40/60 g	35/55 g + 10 g inulin	35/55 g + 10 g inulin	35/55 g + 5 g seed mixture	35/55 g + 5 g seed mixture	
		Oat milk with hibiscus	130/180 mL			+ 5 g sunflower oil		+ 5 g Oleic acid enriched sunflower oil
		Strawberry compote	40 g	30 g + 10 g FOS	30 g + 10 g FOS	+5 g sunflower oil		+5 g oleic oil
		Roasted pine nuts	5 g			Not roasted	Not roasted	Not roasted
		Chervil	5 g					
		Blood orange	1 piece/medium					
		Yogurt smoothie with Mint	250 mL					
**3**	• Pearl barley salad with peanuts vinaigrette topped with roasted peanuts + 1 pear<br>• Yogurt with baked blueberries<br>• Raspberry/chili/and violet cabbage juice	Roasted peanuts	6 g			Not roasted	8 g dark chocolate 70%	8 g dark chocolate 70%
		Mint	5 g					
		Lime	20 g					
		Yogurt/You Go Ghurt	70 g					
		Baked blueberries	10 g					
		Peanut vinaigrette	18/25 g			9/13 g sunflower oil	+ 3 g turmeric	9/13 g oleic oil
		Boiled pearl barley	80/110 g					
		Pear	1 piece/medium	100 g kale	100 g kale	100 g kale		
		Raspberry/chili and violet cabbage juice	250 mL					

Continued.

For peer review only - http://bmjopen.bmj.com/site/about/guidelines.xhtml

Concluded.

Day	Dinner meal	Ingredients	Control (female/male)	Personalised plan substituted ingredient in each cluster*: Carbohydrate	Microbiota	Lipid	Inflammation	Oxidative stress
**1**	Vegetarian massaman curry with cilantro/quinoa and baked tomatoes	Massaman curry	230/325 g	220/315 g with 118 g sweet potato + 10 g inulin	220/315 g + 10 g inulin		With 122 g sweet potatoes + 5 g turmeric	Extra baked carrots - 70 g
		Rapeseed oil	1 teaspoon					
		Sesame oil dressing	20 g			6.7 g sunflower oil		6.7 Oleic oil
		Sesame seeds	4 g					
		Baby spinach	5 g					
		Cilantro	5 g					
		Cooked quinoa	110/150 g			105/145 g + 5 g seed mixture	+5 g seed mixture	
		Tomato	90 g					
		Bell pepper/red	1 piece/medium	+30 g kale	+30 g kale	+20 g kale		
**2**	Baked eggplant with green hummus/bulgur salad and Turkish flatbread	Baked eggplant	65/75 g					
		Sunflower oil	1 teaspoon					
		Flatbread with nigella sativa	120/150 g			+3 g hemp seeds	+4 g turmeric	+3 g hemp seeds
		Green hummus	60/80 g	55/75 g + 5 g inulin	55/75 g + 5 g inulin	6/8 g sunflower oil		6/8 g oleic oil
		Mint cream	40 g					
		Lemon dressing	20 g	20 g lemon dressing with 5 g FOS (replaces sirup)	20 g lemon dressing with 5 g FOS (replaces sirup)	10 g sunflower oil (replaces the canola/olive oil in standard recipe)		10 g oleic oil (replaces the canola/olive oil in standard recipe)
		Parsley	5 g					
		Bulgur	30/45 g					
		Tomato	100 g					
		Courgette	150 g	150 g red cabbage	150 g red cabbage	150 g red cabbage		120 g carrot
**3**	Trofie pasta with cauliflower sauce and fried pak-choi + green bell pepper	Cauliflower sauce	140/190 g	135/185 g + 10 g FOS	135/185 g + 10 g FOS			
		Extra virgin olive oil	2.5 g					
		Mint	5 g					
		Marinated split peas	60/80 g				4 g turmeric + 4 g hemp seed	
		Lime dressing	30 g					
		Pak-choi	90 g	100 g broccoli	100 g broccoli	100 g broccoli		100 g Chinese cabbage
		Bell pepper/green	½ piece (100 g)	75 g kale	75 g kale	75 g kale		150 g red bell pepper
		Pasta/Trofie	120/150 g	Whole grain linguine	Whole grain linguine	Whole grain linguine	Whole grain linguine	Whole grain linguine

**Added or substituted ingredients from the control diet.*
Values are presented as 'female/male' unless equal for both genders.
Abbreviations: FOS, fructooligosaccharides.

For peer review only

For peer review only - http://bmjopen.bmj.com/site/about/guidelines.xhtml

Table 3: Nutritional information of the 3-days menus

Day 1, Breakfast	Control	Carbohydrate	Microbiota	Lipid	Inflammation	Oxidative stress
Energy, kcal	507/601	505/596	515/609	507/601	510/604	529/623
Protein, g (%)	14 (11)/16 (10)	13 (10)/16 (10)	14 (10)/16 (10)	14 (11)/16 (10)	13 (10)/15 (10)	14 (10)/16 (10)
Carbohydrate, g (%)	55 (43)/62 (41)	54 (42)/61 (40)	56 (43)/63 (41)	55 (43)/62 (41)	56 (44)/63 (42)	60 (45)/67 (43)
Dietary fibre, g	13/14	17/18	17/18	13/14	13/14	13/14
Sugars, g	29/31	28/29	29/31	29/31	29/30	35/35
Added sugars, g	0	0	0	0	0	0
Fat, g (%)	24 (41)/30 (44)	24 (41)/30 (44)	24 (41)/30 (43)	24 (41)/30 (44)	24 (41)/31 (44)	24 (41)/31 (44)
Saturated fat, g	4/5	4/5	4/5	4/5	4/5	4/5
Monounsaturated fat, g	3	3/4	3	3	3	3
Polyunsaturated fat, g	6/7	6/7	6/7	6/7	6/7	6/7
Salt, g	0.5	0.5	0.5	0.5	0.5	0.5
**Day 2, Breakfast**						
Energy, kcal	501/608	512/618	512/615	505/612	507/614	499/606
Protein, g (%)	12 (10)/15 (10)	12 (9)/15 (9)	12 (9)/15 (10)	12 (10)/15 (10)	13 (10)/16 (10)	12 (10)/15 (10)
Carbohydrate, g (%)	65 (53)/82 (55)	63 (50)/80 (52)	63 (50)/80 (52)	55 (44)/72 (48)	63 (50)/80 (53)	57 (46)/75 (49)
Dietary fibre, g	10/12	27/29	27/29	11/13	11/13	10/12
Sugars, g	22/24	24/33	22/24	20/23	22/24	21/23
Added sugars, g	0	0	0	0	0	0
Fat, g (%)	19 (33)/21 (31)	18 (31)/21 (29)	18 (31)/21 (29)	24 (42)/27 (38)	20 (35)/23 (33)	23 (41)/25 (37)
Saturated fat, g	3	3	3	4	3/4	3/4
Monounsaturated fat, g	2	2	1/2	3	2	3
Polyunsaturated fat, g	3	3	3	7/8	4/5	6/7
Salt, g	1	1	1	1	1	1
**Day 3, Breakfast**						
Energy, kcal	508/608	510/610	510/610	510/610	528/628	518/625
Protein, g (%)	9 (7)/10 (7)	13 (10)/15 (10)	13 (10)/15 (10)	13 (10)/15 (10)	8 (6)/10 (6)	8 (6)/10 (6)
Carbohydrate, g (%)	68 (53)/78 (52)	60 (48)/71 (47)	60 (48)/71 (47)	60 (48)/71 (47)	71 (54)/82 (52)	69 (53)/82 (54)
Dietary fibre, g	8.2/8.8	11/12	11/12	11/12	9.3/9.8	8.6/9.6
Sugars, g	29	21	21	21	31	31
Added sugars, g	0	0	0	0	0	0
Fat, g (%)	21 (37)/27 (38)	22 (38)/28 (39)	22 (38)/28 (39)	22 (38)/28 (39)	22 (37)/28 (39)	22 (37)/27 (38)

Continued.

For peer review only - http://bmjopen.bmj.com/site/about/guidelines.xhtml

Day 3, Breakfast	Control	Carbohydrate	Microbiota	Lipid	Inflammation	Oxidative stress
Saturated fat, g	3/4	3/4	3/4	3/4	5/6	5/6
Monounsaturated fat, g	5/6	5/6	5/6	5/6	4/6	4/6
Polyunsaturated fat, g	8/11	8/11	8/11	8/11	7/10	7/10
Salt, g	1	1	1	1	1	0.5
**Day 1, Dinner**						
Energy, kcal	697/881	712/890	724/907	725/908	729/907	697/881
Protein, g (%)	19 (11)/25 (11)	19 (11)/25 (11)	20 (11)/26 (11)	21 (11)/26 (12)	20 (11)/25 (11)	19 (11)/25 (11)
Carbohydrate, g	86 (50)/114 (52)	84 (48)/110 (50)	87 (48)/114 (50)	86 (48)/114 (50)	86 (48)/112 (50)	86 (50)/114 (52)
Dietary fibre, g	12/14	23/26	22/25	14/16	15/18	12/14
Sugars, g	24/28	27/33	25/29	24/28	27/32	24/28
Added sugars, g	0	0	0	0	0	0
Fat, g (%)	29 (36)/34 (34)	28 (35)/33 (33)	29 (35)/34 (33)	31 (37)/36 (35)	31 (37)/36 (35)	29 (36)/34 (34)
Saturated fat, g	7/9	7/9	7/9	7/9	8/10	7/9
Monounsaturated fat, g	7	7	7	7/8	7/8	7
Polyunsaturated fat, g	11/13	11/13	11/13	12/14	12/14	11/13
Salt, g	2/3	2/3	2/3	2/3	2/3	2/3
**Day 2, Dinner**						
Energy, kcal	704/871	731/897	731/897	742/912	718/884	742/901
Protein, g (%)	21 (12)/26 (12)	21 (11)/26 (12)	21 (11)/26 (12)	22 (12)/28 (12)	21 (12)/27 (12)	21 (11)/26 (12)
Carbohydrate, g	90 (54)/116 (51)	95 (52)/121 (54)	95 (52)/121 (54)	96 (52)/122 (53)	92 (51)/118 (54)	96 (52)/122 (54)
Dietary fibre, g	14/17	25/28	25/28	15/18	15/18	16/18
Sugars, g	5/6	11/12	11/12	11	5/6	12/13
Added sugars, g	0	0	0	0	0	0
Fat, g (%)	27 (33)/31 (30)	25 (30)/29 (28)	25 (30)/29 (28)	28 (32)/29 (31)	27 (33)/31 (30)	28 (33)/32 (30)
Saturated fat, g	3/4	3/4	3/4	3/4	4	4
Monounsaturated fat, g	12/15	12/14	12/14	13/15	12/15	13/15
Polyunsaturated fat, g	8/9	7/8	7/8	9/10	8/9	9/10
Salt, g	3/4	3/4	3/4	3/4	3/4	3/4
**Day 3, Dinner**						
Energy, kcal	699/877	731/909	731/909	722/900	732/911	723/901
Protein, g (%)	23 (13)/28 (13)	25 (14)/31 (14)	25 (14)/31 (14)	25 (14)/31 (14)	24 (13)/30 (13)	24 (13)/30 (13)
Carbohydrate, g (%)	105 (60)/131 (60)	106 (58)/131 (58)	106 (58)/131 (58)	106 (58)/131 (59)	107 (59)/133 (58)	109 (60)/135 (61)

Continued.

For peer review only - http://bmjopen.bmj.com/site/about/guidelines.xhtml

Concluded.

Day 3, Dinner	Control	Carbohydrate	Microbiota	Lipid	Inflammation	Oxidative stress
Dietary fibre, g	11/13	22/25	22/25	14/16	12/14	11/14
Sugars, g	20/23	19/22	19/22	20/23	20/23	24/27
Added sugars, g	0	0	0	0	0	0
Fat, g (%)	19 (24)/24 (24)	19 (22)/25 (23)	19 (22)/25 (23)	19 (23)/25 (24)	21 (25)/26 (25)	19 (23)/25 (24)
Saturated fat, g	3/4	3/4	3/4	3/4	3/4	3/4
Monounsaturated fat, g	7/10	7/9	7/9	7/10	8/10	7/9
Polyunsaturated fat, g	6/8	6/8	6/8	7/9	7/9	6/8
Salt, g	2/3	2/3	2/3	2/3	2/3	2/3

Values are presented as 'female/male' unless equal for both genders.

For peer review only

For peer review only - http://bmjopen.bmj.com/site/about/guidelines.xhtml

SUPPLEMENTARY ONLINE MATERIAL 3: informed consent version 2.0, 18 September 2020

Title of research project: **PREVENTOMICS: Empowering consumers to PREVENT diet-related diseases through OMICS-based personalized nutrition**

Consent from the study participant:

I have obtained written and oral information about the research project and I am informed about the aim, methods, benefits and risks of participating in the study.

I know that it is completely voluntary to participate, and I have the right to withdraw the informed consent at any time and with no loss of benefits to which I am otherwise entitled.

I consent to participate in the research project and that my biological material will be stored in a research biobank. I have received a copy of this informed consent form as well as a copy of the written information.

Study participant name: ____

Date: ________________ Signature: ____

In case new information that has substantial influence on your health emerges from the research project, you will be informed. Would you prefer **not** to be informed about information that has substantial influence on your health, please mark it here __________ (insert X).

Do you wish to be informed about the final result of the research project and the potential consequences for you:

Yes _____ (insert X) No _____ (insert X)

Consent from the study staff that provided the oral information:

I declare, that the participant has received both written and oral information about the research project.

I declare to the best of my knowledge and belief that the participant has received sufficient information to decide to participate in the research project.

Study staff name: ____

Date: ________________ Signature: ____

National project identification: H-20029882

For peer review only

Reporting checklist for protocol of a clinical trial

SPIRIT

STANDARD PROTOCOL ITEMS: RECOMMENDATIONS FOR INTERVENTIONAL TRIALS

		Reporting Item	Page Number
**Administrative information**			
Title	#1	Descriptive title identifying the study design, population, interventions, and, if applicable, trial acronym	1
Trial registration	#2a	Trial identifier and registry name. If not yet registered, name of intended registry	2
Trial registration: data set	#2b	All items from the World Health Organization Trial Registration Data Set	N/A
Protocol version	#3	Date and version identifier	18,20
Funding	#4	Sources and types of financial, material, and other support	20
Roles and responsibilities: contributorship	#5a	Names, affiliations, and roles of protocol contributors	1,20
Roles and responsibilities: sponsor contact information	#5b	Name and contact information for the trial sponsor	20
Roles and responsibilities: sponsor and funder	#5c	Role of study sponsor and funders, if any, in study design; collection, management, analysis, and interpretation of data; writing of the report; and the decision to submit the report for publication, including whether they will have ultimate authority over any of these activities	18-20

For peer review only

Roles and responsibilities: committees	#5d	Composition, roles, and responsibilities of the coordinating centre, steering committee, endpoint adjudication committee, data management team, and other individuals or groups overseeing the trial, if applicable (see Item 21a for data monitoring committee)	N/A
**Introduction**			
Background and rationale	#6a	Description of research question and justification for undertaking the trial, including summary of relevant studies (published and unpublished) examining benefits and harms for each intervention	4, 5
Background and rationale: choice of comparators	#6b	Explanation for choice of comparators	4, 5
Objectives	#7	Specific objectives or hypotheses	5
Trial design	#8	Description of trial design including type of trial (eg, parallel group, crossover, factorial, single group), allocation ratio, and framework (eg, superiority, equivalence, non-inferiority, exploratory)	6, Figure 1
**Methods: Participants, interventions, and outcomes**			
Study setting	#9	Description of study settings (eg, community clinic, academic hospital) and list of countries where data will be collected. Reference to where list of study sites can be obtained	6
Eligibility criteria	#10	Inclusion and exclusion criteria for participants. If applicable, eligibility criteria for study centres and individuals who will perform the interventions (eg, surgeons, psychotherapists)	6,7

For peer review only

Interventions: description	#11a	Interventions for each group with sufficient detail to allow replication, including how and when they will be administered	7-13, Tables 1-5, Supplemental material 1 and 2
Interventions: modifications	#11b	Criteria for discontinuing or modifying allocated interventions for a given trial participant (eg, drug dose change in response to harms, participant request, or improving / worsening disease)	6, 17
Interventions: adherance	#11c	Strategies to improve adherence to intervention protocols, and any procedures for monitoring adherence (eg, drug tablet return; laboratory tests)	13
Interventions: concomitant care	#11d	Relevant concomitant care and interventions that are permitted or prohibited during the trial	10-13, Table 4
Outcomes	#12	Primary, secondary, and other outcomes, including the specific measurement variable (eg, systolic blood pressure), analysis metric (eg, change from baseline, final value, time to event), method of aggregation (eg, median, proportion), and time point for each outcome. Explanation of the clinical relevance of chosen efficacy and harm outcomes is strongly recommended	13-17, Table 5
Participant timeline	#13	Time schedule of enrolment, interventions (including any run-ins and washouts), assessments, and visits for participants. A schematic diagram is highly recommended (see Figure)	6, 7, Figure 2
Sample size	#14	Estimated number of participants needed to achieve study objectives and how it was determined, including clinical and statistical assumptions supporting any sample size calculations	18
Recruitment	#15	Strategies for achieving adequate participant enrolment to reach target sample size	6

For peer review only

**Methods: Assignment of interventions (for controlled trials)**			
Allocation: sequence generation	#16a	Method of generating the allocation sequence (eg, computer-generated random numbers), and list of any factors for stratification. To reduce predictability of a random sequence, details of any planned restriction (eg, blocking) should be provided in a separate document that is unavailable to those who enrol participants or assign interventions	9
Allocation concealment mechanism	#16b	Mechanism of implementing the allocation sequence (eg, central telephone; sequentially numbered, opaque, sealed envelopes), describing any steps to conceal the sequence until interventions are assigned	9
Allocation: implementation	#16c	Who will generate the allocation sequence, who will enrol participants, and who will assign participants to interventions	9
Blinding (masking)	#17a	Who will be blinded after assignment to interventions (eg, trial participants, care providers, outcome assessors, data analysts), and how	9
Blinding (masking): emergency unblinding	#17b	If blinded, circumstances under which unblinding is permissible, and procedure for revealing a participant's allocated intervention during the trial	N/A
**Methods: Data collection, management, and analysis**			
Data collection plan	#18a	Plans for assessment and collection of outcome, baseline, and other trial data, including any related processes to promote data quality (eg, duplicate measurements, training of assessors) and a description	13-17, Table 5

For peer review only

		of study instruments (eg, questionnaires, laboratory tests) along with their reliability and validity, if known. Reference to where data collection forms can be found, if not in the protocol	
Data collection plan: retention	#18b	Plans to promote participant retention and complete follow-up, including list of any outcome data to be collected for participants who discontinue or deviate from intervention protocols	13
Data management	#19	Plans for data entry, coding, security, and storage, including any related processes to promote data quality (eg, double data entry; range checks for data values). Reference to where details of data management procedures can be found, if not in the protocol	18-19
Statistics: outcomes	#20a	Statistical methods for analysing primary and secondary outcomes. Reference to where other details of the statistical analysis plan can be found, if not in the protocol	18
Statistics: additional analyses	#20b	Methods for any additional analyses (eg, subgroup and adjusted analyses)	18
Statistics: analysis population and missing data	#20c	Definition of analysis population relating to protocol non-adherence (eg, as randomised analysis), and any statistical methods to handle missing data (eg, multiple imputation)	18
**Methods: Monitoring**			
Data monitoring: formal committee	#21a	Composition of data monitoring committee (DMC); summary of its role and reporting structure; statement of whether it is independent from the sponsor and competing interests; and reference to where further details about its charter can be found, if not in the protocol. Alternatively, an explanation of why a DMC is not needed	17

For peer review only

Data monitoring: interim analysis	#21b	Description of any interim analyses and stopping guidelines, including who will have access to these interim results and make the final decision to terminate the trial	17
Harms	#22	Plans for collecting, assessing, reporting, and managing solicited and spontaneously reported adverse events and other unintended effects of trial interventions or trial conduct	17
Auditing	#23	Frequency and procedures for auditing trial conduct, if any, and whether the process will be independent from investigators and the sponsor	N/A
**Ethics and dissemination**			
Research ethics approval	#24	Plans for seeking research ethics committee / institutional review board (REC / IRB) approval	18, 19
Protocol amendments	#25	Plans for communicating important protocol modifications (eg, changes to eligibility criteria, outcomes, analyses) to relevant parties (eg, investigators, REC / IRBs, trial participants, trial registries, journals, regulators)	18
Consent or assent	#26a	Who will obtain informed consent or assent from potential trial participants or authorised surrogates, and how (see Item 32)	18, 19
Consent or assent: ancillary studies	#26b	Additional consent provisions for collection and use of participant data and biological specimens in ancillary studies, if applicable	18, 19
Confidentiality	#27	How personal information about potential and enrolled participants will be collected, shared, and maintained in order to protect confidentiality before, during, and after the trial	17, 18

For peer review only

Declaration of interests	#28	Financial and other competing interests for principal investigators for the overall trial and each study site	20
Data access	#29	Statement of who will have access to the final trial dataset, and disclosure of contractual agreements that limit such access for investigators	17, 19
Ancillary and post trial care	#30	Provisions, if any, for ancillary and post-trial care, and for compensation to those who suffer harm from trial participation	N/A
Dissemination policy: trial results	#31a	Plans for investigators and sponsor to communicate trial results to participants, healthcare professionals, the public, and other relevant groups (eg, via publication, reporting in results databases, or other data sharing arrangements), including any publication restrictions	19
Dissemination policy: authorship	#31b	Authorship eligibility guidelines and any intended use of professional writers	19
Dissemination policy: reproducible research	#31c	Plans, if any, for granting public access to the full protocol, participant-level dataset, and statistical code	N/A
**Appendices**			
Informed consent materials	#32	Model consent form and other related documentation given to participants and authorised surrogates	Supplemental Material 3
Biological specimens	#33	Plans for collection, laboratory evaluation, and storage of biological specimens for genetic or molecular analysis in the current trial and for future use in ancillary studies, if applicable	16, 17

The SPIRIT Explanation and Elaboration paper is distributed under the terms of the Creative Commons Attribution License CC-BY-NC. This checklist was completed on 02. March 2021 using https://www.goodreports.org/, a tool made by the EQUATOR Network in collaboration with Penelope.ai

For peer review only

BMJ Open

Empowering consumers to PREVENT diet-related diseases through OMICS sciences (PREVENTOMICS): Protocol for a parallel double-blinded randomised intervention trial to investigate biomarker-based nutrition plans for weight loss

Journal:	*BMJ Open*
Manuscript ID	bmjopen-2021-051285.R2
Article Type:	Protocol
Date Submitted by the Author:	25-Jan-2022

Complete List of Authors:	Aldubayan, Mona A.; University of Copenhagen Faculty of Science, Nutrition, Exercise and Sports; King Saud bin Abdulaziz University for Health Sciences, College of Applied Medical Sciences<br>Pigsborg, Kristina; University of Copenhagen Faculty of Science, Nutrition, Exercise and Sports<br>Gormsen, Sophia; Simple Feast<br>Serra, Francisca; University of the Balearic Islands, Laboratory of Molecular Biology, Nutrition and Biotechnology - NUO group; Alimentómica S.L., Spin-off n.1 of the University of the Balearic Islands<br>Palou, Mariona; University of the Balearic Islands, Laboratory of Molecular Biology, Nutrition and Biotechnology - NUO group; Alimentómica S.L., Spin-off n.1 of the University of the Balearic Islands<br>Mena, Pedro; University of Parma, Department of Food and Drug, Human Nutrition Unit<br>Wetzels, Mart; Onmi: Behaviour Change Technology<br>Calleja , Alberto; Grupo Carinsa<br>Caimari, Antoni; Eurecat Centre Tecnològic de Catalunya, Biotechnology Area, Nutrition and Health Unit<br>Del Bas, Josep; Eurecat Centre Tecnològic de Catalunya, Biotechnology Area, Nutrition and Health Unit<br>Gutierrez, Biotza; Eurecat Centre Tecnològic de Catalunya, Biotechnology Area, Nutrition and Health Unit<br>Magkos, Faidon ; University of Copenhagen, Faculty of Science, Nutrition, Exercise and Sports<br>Hjorth, Mads; Novo Nordisk Foundation, Healthy Weight Center

**Primary Subject Heading**:	Nutrition and metabolism
Secondary Subject Heading:	Public health
Keywords:	NUTRITION & DIETETICS, PUBLIC HEALTH, GENETICS

Empowering consumers to PREVENT diet-related diseases through OMICS sciences (PREVENTOMICS): Protocol for a parallel double-blinded randomised intervention trial to investigate biomarker-based nutrition plans for weight loss

Mona A. Aldubayan[1,2], Kristina Pigsborg[1], Sophia M. O. Gormsen[3], Francisca Serra[4], Mariona Palou[4], Pedro Mena[5], Mart Wetzels[6], Alberto Calleja[7], Antoni Caimari[8], Josep M del Bas[8], Biotza Gutierrez[8], Faidon Magkos[1], Mads F. Hjorth[9]

[1]Department of Nutrition, Exercise and Sports, Faculty of Science, University of Copenhagen, Denmark.

[2]King Saud bin Abdulaziz University for Health Sciences, Riyadh, Saudi Arabia.

[3]Simple Feast, Copenhagen, Denmark.

[4]Laboratory of Molecular Biology, Nutrition and Biotechnology; NUO group. Universitat de les Illes Balears, Palma, Spain; Institut d'Investigació Sanitària Illes Balears (IdISBa), Palma, Spain; CIBER de Fisiopatología de la Obesidad y Nutrición (CIBEROBN), Madrid, Spain and Alimentómica S.L., Spin-off n.1 of the University of the Balearic Islands, Palma, Spain.

[5]Human Nutrition Unit, Department of Food and Drug, University of Parma, Parma, Italy.

[6]ONMI: Behaviour Change Technology, Eindhoven, The Netherlands.

[7]Grupo Carinsa, Sant Quirze del Valles, Barcelona, Spain.

[8]Eurecat, Centre Tecnològic de Catalunya, Biotechnology Area, Nutrition and Health Unit, Reus, Spain.

[9]Healthy Weight Centre, Novo Nordisk Foundation, Tuborg Havnevej 19, 2900 Hellerup, Denmark.

Corresponding author:

Mona Adnan Aldubayan
Department of Nutrition, Exercise and Sports,
Faculty of Science, University of Copenhagen
Rolighedsvej 26, 1958 Frederiksberg, Denmark
Phone: +45 91991923
E-mail: monal@nexs.ku.dk

Word count: 5145 (excluding title page, abstract, references, figures and tables).

For peer review only

ABSTRACT

Introduction: Personalised nutrition holds immense potential over conventional one-size-fits-all approaches for preventing and treating diet-related diseases, such as obesity. The current study aims to examine whether a personalised nutritional plan produces more favourable health outcomes than a standard approach based on general dietary recommendations in subjects with overweight or obesity and elevated waist circumference.

Methods and analysis: This project is a 10-week parallel, double-blinded randomised intervention trial. We plan to include 100 adults aged 18-65 years interested in losing weight, with body mass index ≥27 but <40 kg/m^2 and elevated waist circumference (males >94 cm; females >80 cm). Participants will be categorized into one of five predefined 'clusters' based on their individual metabolic biomarker profile and genetic background, and will be randomised in a 1:1 ratio to one of two groups: (1) personalised plan group that will receive cluster-specific meals every day for 6 days a week, in conjunction with a personalised behavioural change program via electronic push notifications; or (2) control group that will receive meals following the general dietary recommendations in conjunction with generic health behaviour prompts. The primary outcome is the difference between groups (personalised vs. control) in the change in fat mass from baseline. Other outcome measures include changes in body weight and waist circumference, lipid profile, fasting blood glucose, adipokines, inflammatory biomarkers, blood pressure, physical activity and sleep patterns, health-related quality of life, eating behaviour, biomarkers of food intake, attitude to weight management diets and dietary intake. The effect of the intervention on the primary outcome will be analysed by means of linear mixed models.

Ethics and dissemination: The protocol has been approved by the Ethics Committee of the Capital Region, Copenhagen, Denmark. Study findings will be disseminated through peer-reviewed publications, conference presentations and media outlets.

Trial registration number: ClinicalTrials.gov registry (NCT04590989).

Keywords: Personalised nutrition, Precision nutrition, Nutrigenomics, Nutrigenetics, Metabolomics, Obesity, Overweight, Weight management, Body weight

For peer review only

Strengths and limitations of this study

- This study may identify novel approaches in facilitating weight loss and health-promoting behaviours by applying state-of-the-art knowledge that integrates metabolomics and genetics with nutrition.
- The trial is double-blinded, which is rare in nutritional science, and serves as proof of concept for the personalised dietary management of obesity.
- A potential limitation is that both groups are receiving healthy foods and behavioural advice, which may mask the hypothesised intervention effect of the personalised plan.
- The study is powered to detect differences in 10-week body fat loss between intervention and control arms and not within each of the five clusters; differences between the latter will be assessed by post-hoc analysis.
- Potential long-term effects of a personalised approach cannot be evaluated from this 10-week study; however, results will provide a basis for implementation in longer obesity-management programs.

For peer review only - http://bmjopen.bmj.com/site/about/guidelines.xhtml

INTRODUCTION

The ultimate goal of nutrition research and dietary recommendations is the promotion of human health and the prevention or treatment of chronic diseases.[1] Still, the global prevalence rate of nutrition-related non-communicable diseases (NCDs) continues to rise rapidly.[2] There is considerable evidence indicating that obesity is a major risk factor for developing NCDs including type 2 diabetes, cardiovascular diseases, and certain types of cancers, which are the leading causes of morbidity and mortality.[3 4] Therefore, obesity puts a great burden on the individual, the healthcare system, and society.[3] Accordingly, enormous efforts to tackle this epidemic have been implemented from health professionals through setting different policies and guidelines for the public, but with little success, as management of obesity remains a very challenging task. Moreover, the optimal diet characteristics—particularly with respect to dietary macronutrient composition (e.g., low-carbohydrate, low-fat, high-protein diets)—that are most effective in reducing excess weight gain or promoting weight loss have long been debated.[5 6] Clinical trials have demonstrated that certain individuals benefit more from a particular dietary intervention than others in reducing body weight, while only a small number are able to keep the weight off in the long-term.[7 8] This implies there is no strong evidence that one diet is superior to others for inducing weight loss, and there is no such thing as a "perfect" diet for everyone. Such substantial interindividual variation in response to any given dietary treatment can be attributed to multiple phenotypic factors and genetic variants which influence how the body utilises and metabolises nutrients.[7] This gives rise to the demand for customising diet plans and nutrition advice at the individual or small group level, rather than at the population level. Recent developments in "omics" technologies (nutrigenomics, transcriptomics, epigenomics, metabolomics, metagenomics) offer exciting opportunities to explore the complex interplay between nutrition, genetics and metabolism.[9] By integrating these novel tools with bioinformatics, the potential of "personalised nutrition" can be implemented through identifying novel biomarkers that can predict the most effective diet for weight loss and improved health outcomes for any given individual.[9-11] Therefore, the ability to provide evidence-based dietary advice based on individual genetic make-up, phenotypic information on anthropometry, biochemical and metabolic profiles, physical activity habits, and medical history—among others—may lead to changed behaviours and ultimately, improved health.

In this context, H2020 PREVENTOMICS (Empowering consumers to PREVENT diet-related diseases through OMICS sciences), coordinated by Eurecat in Spain, has developed a platform with a Decision Support System (DSS) tool that integrates individual phenotypic characteristics at the metabolome level with their genotype, lifestyle habits, and preferences to improve their health status through personalised nutrition management plans. The project aims to examine the validity of the PREVENTOMICS platform in terms of its

For peer review only - http://bmjopen.bmj.com/site/about/guidelines.xhtml

potential for personalisation at different levels of the food value chain. This will be achieved through different intervention studies in Denmark, Spain, and Poland and the United Kingdom, with both healthy volunteers and volunteers with abdominal obesity. Here we report the specific characteristics of the Danish study protocol.

Research hypothesis and aims

The overall aim of this 10-week randomised trial is to examine the efficacy of the PREVENTOMICS platform, integrated in an e-commerce digital tool created for delivering personalised meals for producing more favourable health outcomes than meals based on general dietary recommendations, in subjects with overweight or obesity and elevated waist circumference. In addition, the intervention group will receive tailored and actionable behaviour change prompts whereas the control group will receive general nutrition and lifestyle advice. Our hypothesis is that the personalised dietary and behavioural treatment plan will produce greater reductions in fat mass and body weight, and will promote more favourable changes in circulating metabolic and inflammatory biomarkers compared to the control dietary and behavioural treatment plan.

Primary and secondary objectives

The primary goal of this study is to evaluate the change in body fat mass between the personalised plan group and the control group over the 10-week intervention period. The secondary goal is to evaluate the change in the following health outcomes between the personalised and control groups: (1) body composition (visceral and subcutaneous fat, lean body mass, weight, body mass index [BMI], waist circumference); (2) lipid profile (total cholesterol, low-density lipoprotein [LDL], high-density lipoprotein [HDL], oxidized LDL [oxLDL], triglycerides); (3) glucose homeostasis (glucose, insulin, homeostatic model assessment of insulin resistance [HOMA-IR]); (4) inflammatory markers (C-reactive protein [CRP], interleukin 6 [IL-6], interleukin 10 [IL-10], monocyte chemoattractant protein-1 [MCP-1], tumour necrosis factor alpha [TNFα], soluble amino acid residue glycosylated peptide-1 [ICAM-1], soluble cluster of differentiation-14 [CD-14]); (5) adipokines (leptin, adiponectin); (6) liver health markers (alanine transaminase [ALT], gamma-glutamyl transferase [GGT]); (7) renal health markers (uric acid, creatinine); and (8) blood pressure.

For peer review only - http://bmjopen.bmj.com/site/about/guidelines.xhtml

METHODS AND ANALYSIS

Study design

This is a randomised, single-centre, parallel-group (1:1 ratio), double-blinded intervention study conducted at the research facilities of the Department of Nutrition, Exercise and Sports (NEXS), University of Copenhagen, Denmark. The study protocol adheres to the SPIRIT (Standard Protocol Items: Recommendations for Interventional Trials) guidelines.[12] This study was registered at ClinicalTrials.gov in October 2020 (NCT04590989), and recruitment started at NEXS immediately after. Information needed to determine the 'cluster' of participants was collected in January 2021, and the actual analysis of biological samples and clustering was performed in February-March 2021. All data collected during the 10-week intervention period (March-June 2021) are expected to be fully analysed by the end of 2021. The overall study design is illustrated in Figure 1.

Patient and Public Involvement: Patients and the public were not involved in the design, conduct or reporting of this study.

Study participants

Participants are males and females aged 18–65 years with a BMI of ≥27 but <40 kg/m^2 and elevated waist circumference (males >94 cm; females >80 cm). Participants should possess a smartphone and be able to provide an informed consent. The exclusion criteria are as follows: (1) diagnosis of diabetes; (2) history or diagnosis of heart, liver or kidney diseases; (3) chronic diseases, e.g., cancer within the past 5 years (except adequately-treated localized basal cell skin cancer); (4) use of drugs (e.g., antibiotics) that, in the opinion of the medically responsible investigator, are likely to affect the primary outcomes of the study; (5) being lactating, pregnant or planning to become pregnant within the study period; (6) self-reported weight change of >5 % within two months prior to screening; (7) participation in another clinical trial; (8) other blood donation during the study; (9) having allergies or food intolerances; (10) no or limited access to the internet. Participants unable to comply with the study protocol, as judged by the investigator, are also excluded.

Recruitment procedure

The study flow chart is summarized in Figure 2. Potential participants were recruited through internet-based advertisements. Trained study personnel contacted 220 subjects who expressed interest in the study via telephone to determine initial eligibility (pre-screening). Written information about the study was provided to 120 potential participants who were deemed eligible from the telephone pre-screening and

For peer review only - http://bmjopen.bmj.com/site/about/guidelines.xhtml

scheduled for an oral information meeting (visit 0, V0) at the department (NEXS). If the subject signed the informed consent, either immediately following the information meeting or after a few days of consideration, they were screened according to the inclusion/exclusion criteria to assess final eligibility. A total of 106 participants were recruited and invited for the pre-baseline visit (V1) where anthropometric measurements, blood, saliva, and urine samples were collected, and various questionnaires were filled out. One hundred participants completed V1 and had their samples sent to the assigned consortium for analysing data on subjects' metabolome and genotype in addition to lifestyle habits, preferences, and physiological status. These data are utilized to determine subjects' cluster (see later) and develop the personalised dietary plans for the subsequent 10-week intervention period.

Cluster allocation

All subjects were categorized into one of five predefined 'clusters' (Table 1) based on their metabolic and genetic biomarkers collected at V1, according to the following procedure:

First, collected samples of urine, plasma and serum were analysed to assess a total of 58 biomarkers relevant to the following five metabolic processes: 1) oxidative stress; 2) inflammation; 3) carbohydrate metabolism; 4) lipid metabolism; and 5) gut microbiota metabolism. References supporting the rationale for using the biomarkers listed in Table 1 are included in the online supplemental material 1.

Table 1: Full list of biomarkers in relation to the metabolic clusters.

Carbohydrate	Lipid	Inflammation	Oxidative stress	Microbiota
Glucose	LDL-cholesterol	CRP	8-iso-PGF2α	TMA
HOMA-IR	Total cholesterol	N-acetylglycoproteins	8-OHdG	TMAO
Glutamate	PUFAs	MCP-1	oxidized LDL	Betaine
Uric acid	HDL-cholesterol	TNFα	Uric acid	Choline
Leptin	SFAs	IL-6	Allantoin	DMA
Adiponectin	Triglycerides	IL-10	Betaine	Dimethylglycine
Insulin	MUFAs	SFAs	Pseudouridine	LBP
Tyrosine	LPC	sICAM-1	Dimethylglycine	Succinate
Propionylcarnitine	Linoleic acid	LPC	Methionine	Lactate
Lactate	DHA	LBP	Glycine	Acetate
Valine	Oleic acid	DHA C20:3		
Leucine	Choline	sCD14		
Isoleucine	3-hydroxybutyrate	Linoleic acid C18:2		
Phenylalanine	Propionylcarnitine	PUFAs		

For peer review only - http://bmjopen.bmj.com/site/about/guidelines.xhtml

Glutamine	Adiponectin
	Leptin

Some biomarkers help define more than one cluster. Abbreviations: HOMA-IR, homeostatic model assessment of insulin resistance; LDL, low-density lipoprotein; HDL, high-density lipoprotein; PUFAs, polyunsaturated fatty acids; MUFAs, monounsaturated fatty acids; SFAs, saturated fatty acids; LPC, lysophosphatidylcholine; DHA, docosahexaenoic acid; CRP, C-reactive protein; MCP-1, monocyte chemoattractant protein-1; TNFα, tumour necrosis factor alpha; IL-6, interleukin-6; IL-10, interleukin-10; sICAM-1, soluble amino acid residue glycosylated peptide-1; sCD14, soluble cluster of differentiation-14; LBP, lipopolysaccharide binding protein; 8-iso-PGF2α, prostaglandin, 8-iso-prostaglandin F2α; 8-OHdG, 8-hydroxydeoxyguanosine; TMA, trimethylamine; TMAO, trimethylamine N-oxide; DMA, dimethylamine.

Second, a core of 35 different single nucleotide polymorphisms (SNPs) which are associated with the five metabolic processes and are able to modulate the biomarker levels reported in Table 1 have been identified by Alimentomica (Spain) to be analysed in saliva samples (Table 2). The biomarkers of the lipid cluster are able to be modulated, at different degree, by a set of 18 SNPs in 14 genes[13-20], the carbohydrate cluster is represented by a panel of 12 SNPs in 11 genes[14 15 21-24] and the inflammation cluster by 8 SNPs in 7 genes.[25-28] Parse scientific data deal with the genetic impact on the specific biomarkers of the oxidative stress cluster; the corresponding genetic risk score comprises 7 genetic variants in 6 genes associated with reduced ability to buffer the oxidative stress associated with low levels of plasma antioxidants.[29-33] In relation to the microbiota cluster, the current evidence base does not provide enough data in support of the role of SNPs (included in the panel or not) and some evidence concerning the host genetic influence on microbiota response and on microbiota metabolite production is neither robust nor sufficient; hence no genetic influence was adopted in this cluster.

Finally, the specific SNPs and the biomarkers in the five metabolic processes will be used—by means of a proprietary algorithm—to calculate individual scores for each of the five metabolic clusters for any given participant. Each subject is then assigned to the metabolic cluster with the highest score. Briefly, the individual biomarkers, both metabolic and genetic, are combined into metabolic clusters considering both the absolute value of the biomarker in the biofluid and the biological relevance of the biomarker within the metabolic cluster. Whereas the first value is directly obtained from blood and urine measurements (metabolomics and proteomics biomarkers) and saliva (genotyping), the second is obtained from different approaches combining artificial intelligence applied to measurements of different biobank samples and literature review.[34] Therefore, the resulting score for each cluster is not based on the definition of thresholds but on the contribution of all individual biomarkers analysed. The specifics of the algorithm cannot be disclosed due to a pending intellectual property rights (IPR) application.

For peer review only - http://bmjopen.bmj.com/site/about/guidelines.xhtml

Table 2: List of SNPs in relation to the metabolic clusters

Lipid		Carbohydrate		Oxidative		Inflammation	
**Gene**	**SNP**	**Gene**	**SNP**	**Gene**	**SNP**	**Gene**	**SNP**
ADIPOQ	rs182052	ADIPOQ	rs182052	COMT	rs4680	APOE	rs429358
APOA5	rs12272004	ASCL1	rs17450122	CPS1	rs1047891	CADM3-AS1	rs12075
APOA5	rs662799	FADS1, FADS2	rs174550	CPS1	rs715	CUX1	rs409224
APOE	rs7412	GCKR	rs1260326	FGF21	rs838133	FADS1	rs174547
APOE	rs429358	GCKR	rs780093	GSTP1	rs1695	GCKR	rs1260326
CUX1	rs409224	GLS2, SPRYD4	rs2657879	MTHFR	rs1801133	GCKR	rs780093
FADS1	rs174547	LEP	rs10487505	SOD2	rs4880	ICAM1	rs5498
GCKR	rs780093	PPARG	rs1801282			IL-6	rs1800795
GCKR	rs1260326	SLC16A10	rs14399				
HFE	rs1800562	SLC16A9	rs1171614				
LEP	rs10487505	SLC2A2	rs8192675				
LPL	rs268	TCF7L2	rs7903146				
LPL	rs326						
PNPLA3	rs738409						
PPID	rs8396						
SLC16A9	rs1171614						
TIMP3	rs12678919						
TRIM58	rs3811444						

Randomisation and concealment

Prior to the intervention, all participants are stratified by metabolic cluster (oxidative stress; inflammation; carbohydrate metabolism; lipid metabolism; microbiota-generated metabolites) and then randomly assigned to either the control or the intervention group, in a 1:1 allocation ratio, by using a computer-generated randomisation sequence with random permuted block sizes of two subjects within each stratum. The person responsible for randomisation and generating the code does not take part in the inclusion and examination of study participants.

In order to maintain blinding, the clustering results for all participants are shared by Eurecat before the baseline visit (V2) with a member of the staff at NEXS (not through the platform) who is only responsible for randomisation. Consequently, the allocation information is incorporated into each participant's profile in the PREVENTOMICS platform after passing back the randomisation information to Eurecat by the staff member at NEXS. However, this field is visible only to SimpleFeast (Denmark) and ONMI (The Netherlands; behavioural change technology, https://www.onmi.design/) user accounts for appropriate delivery of food and behavioural prompts. The profile includes the assigned group (personalised or control) and cluster, in addition to relevant recommendations to each subject. The local team of investigators at NEXS as well as participants are therefore unaware of the clustering and the randomisation of the participants as this field

For peer review only

is hidden from their user account. Moreover, the statistical analyses of the main outcome variable will be conducted without breaking the code for the intervention treatment until the primary analyses have been finalized.

Interventions

1. Dietary intervention

During the 10-week intervention, the personalised plan and control groups receive easy-to-prepare meal boxes twice a week from Simple Feast (Copenhagen, Denmark) complying with the national dietary guidelines of macronutrient distributions.[35] Each delivery provides meal boxes of breakfast and dinner for the subsequent three days (12 meals/week). Meal boxes for the two groups are designed to be visually identical. Moreover, food by Simple Feast is vegetarian and organically produced, however, participants are allowed to eat non-organic/non-vegetarian foods as part of the meals not provided. The number of meals provided to the participants was decided upon a combination of factors including budgetary limitations, practical reasons, and behavioural factors. For the days for which meals are not provided (Saturdays), as well as for all lunches, participants are encouraged to refer to the recipe recommendations that are presented through the Simple Feast Recipe App, so they prepare meals as similar as possible to the group and cluster they are assigned to. All provided foods, including the recipes, are plant-based and align with the recommended list of foods created by Eurecat for each group/cluster (see below).

The calorie content of meals was calculated based on the average daily energy requirements for the general population, which is 2000 kcal/day for females and 2500 kcal/day for males.[36] Given that 25% of daily energy is commonly consumed at breakfast and 35% at dinner, breakfast was designed to provide approximately 500 and 625 kcal/day (for females and males, respectively) and dinner to provide approximately 700 and 875 kcal/day (for females and males, respectively). Participants are instructed to consume the provided meals in whole, or until they are fully satisfied. In addition, they are advised to be inspired by the breakfast and dinner meals provided during this 10-week trial and to consume similar foods for lunch and limit intake of energy-dense foods and drinks. Plant-based meals are fibre-rich and induce greater and faster satiety.[37-39] We thus anticipate lower food consumption both for the provided meals but also outside them (i.e., during lunch or when snacking), which will hopefully be large enough to produce the calorie deficit needed for body weight and fat loss. It is also anticipated that the personalised plan induces favourable changes in eating behaviour and physiological and metabolic parameters that promote body weight and fat loss when compared to the control plan.

The Eurecat Nutrition Team has prepared a list of recommended food items to increase, decrease, or completely exclude from the diet for the control and each cluster in the personalised group. The list was

For peer review only - http://bmjopen.bmj.com/site/about/guidelines.xhtml

adopted by Simple Feast in creating five different menus that encompass 12 meals/week for the five different clusters in the intervention group in addition to the menu for the control group. Contrary to the control meals, personalised meals also include some bioactive compounds provided by CARINSA (Spain). These bioactive compounds were selected following review of the literature and are believed to benefit especially—or exclusively—the metabolic function of individuals in the corresponding cluster (Table 3). Each participant received approximately 20 g of functional ingredient per day, except for the inflammation cluster (6-8 g per day). The macronutrient distribution of the diets between clusters differed only in the amount of fibre. Dietary fibre content was higher in the Carbohydrate and Microbiota clusters as these clusters received fructooligosaccharide (FOS) and inulin as functional ingredients. Nutritional information on macronutrient content and the bioactive ingredients of the meals for each cluster in the personalised and control groups, as well as an example of a 3-day menu, are provided in the online supplementary material 2.

Table 3: Recommended foods and functional ingredients for each metabolic cluster

**Carbohydrate**	Functional ingredient: *FOS and †Inulin<br>Prebiotics: fibre-rich plants (Jerusalem artichoke, onion, leek, asparagus, kale)
**Microbiota**	Functional ingredient: *FOS and †Inulin<br>Prebiotics: fibre-rich plants (Jerusalem artichoke, onion, leek, asparagus, kale)<br>Fermented vegetables<br>Vegetables rich in fibre
**Lipid**	Functional ingredient: sunflower oil<br>Raw nuts and seeds<br>Omega 3 and 6: chia seeds, hemp seeds, walnuts, flax seeds<br>Vegetables rich in fibre
**Inflammation**	Functional ingredient: turmeric powder<br>Raw nuts and seeds<br>Omega 3 and 6: chia seeds, hemp seeds, walnuts, flax seeds<br>Dark chocolate
**Oxidative stress**	Functional ingredient: oleic acid enriched sunflower oil.<br>Raw nuts and seeds<br>Orange, yellow, red coloured vegetables (rich in vitamin A, C, E)<br>Dark chocolate<br>Vegetables rich in fibre

*Fructooligosaccharide originates from partial hydrolysis of chicory roots. †Inulin is extracted from chicory roots.

2. Behavioural assessment and intervention

All participants are asked to fill out a behavioural questionnaire at baseline (V2), in order to collect information about certain habits or behaviours that affect physical, emotional, or mental well-being. During the 10-week intervention period, both groups are enrolled in a behavioural program delivered through ONMI's App with 2-3 electronic push notifications per week. Subjects randomised to the personalised group receive behavioural prompts (active Do's) from the predefined ONMI's evidence-based behavioural

For peer review only

For peer review only - http://bmjopen.bmj.com/site/about/guidelines.xhtml

change program, which has been developed to increase behavioural flexibility and facilitate adoption of healthier habits.[40] For the purposes of this trial, the personalised group Do's (from ONMI) are based on subject's reports from the behavioural questionnaire at V2 in addition to inputs from the nutritional recommendations (from the Eurecat Nutrition Team) via the PREVENTOMICS platform, to provide a comprehensive behavioural change and improve adherence to the dietary intervention. For example, if a participant was recommended to eat kale and brussels sprouts, they could get a message like: "*Our analysis shows kale and brussels sprouts are good for you and should be part of your diet. Find out how much you should be consuming. Do it now*". Table 4 illustrates the different types and quantity of the Do's and massages delivered to personalised and control groups. The messages delivered to the control group are not personalised and are mostly informational in nature rather than prompting participants to take a specific action (i.e., general guidelines available from the National Health Service and the World Health Organization). The personalised and control groups receive the same behavioural treatment in terms of volume (frequency and intensity); the content of messages differs between groups as reflected in the numbers of specific types of messages delivered to each group, but the total number of messages is very similar (Table 4).

Table 4: The type and number of behavioural messages delivered by ONMI to the participants in the personalised and control groups

Type of Messages	Quantity	Description	Example	PP	C
Starter Do	1	Easy start of the program upon behavioural questionnaire completion at V2	SWITCH SEATS DAY! Move some seating around today. Sit somewhere different at meals/ when working/when watching TV. Get a new view! -- Shaking up old habits is good for you and puts you back in charge of your life. Try something new regularly. Make every day count! --	✓	✓
General Do	5	Apply to everyone, relatively easy, to get user hooked to the program	NEW WAY DAY. Take a detour today, go the prettiest route not the shortest. Allow more time, smile at people. Spot 3 beautiful things along the way. -- Wakey Wakey. Regularly challenging our brain keep us alert and interesting. When we take notice of our surroundings we start to live life to the fullest.	✓	
Personalised Do	10	Based on behavioural questionnaire	WHAT ARE YOU EATING FOR? Back off from boredom, address your stress. Get busy, unwind, release your emotions so you only eat when you're hungry today.	✓	
System Message	3	Encouragements, tips, manage expectations	HEALTH TIP. Regular contact with friends and family is key to good mental and physical health. Connections give meaning and purpose to our lives, even when it is digitally.	✓	

For peer review only - http://bmjopen.bmj.com/site/about/guidelines.xhtml

Expander Do	3	Prompt user to explore new parts of personality, based on behavioural questionnaire	EXPANDER: It's NO Day today. Don't say yes when you really want to say no. Give no reason or excuse. Just say, 'Sorry, but the answer's no'.	✓	
Preventomics Messages	6	Template messages that use inputs from the nutritional recommendations of food to increase	PREVENTOMICS: Are you getting the right amount of {{.R1}} and {{.R2}} in your diet? Go online and find some interesting recipes to try at home. Do it now.	✓	
General Messages	24	Recommendations from the NHS and WHO on eating, eating out, exercise, check-ups, help and support, balanced diet	Eating a healthy, balanced diet is an important part of maintaining good health, and can help you feel your best. This means eating a wide variety of foods in the right proportions, and consuming the right amount of food and drink to achieve and maintain a healthy body weight.		✓

Abbreviations: PP, personalised plan; C, control; NHS, The National Health Service; WHO, World Health Organization.

Compliance and food intake biomarkers

Dietary adherence is assessed twice a week—through an electronic questionnaire—by reporting the proportion of food consumed from the meals provided by Simple Feast in the previous three days. For example, in response to the question “*How much of your breakfast did you eat on day 1?*” the possible answers can be: a) Nothing or very little (0-30%); b) Approximately half (30-70%); c) Almost everything or everything (70-100%).

Overall compliance to the diet and the behavioural program is measured at the end of the trial by a six-point Likert scale question ranging from 1 (not at all compliant) to 6 (completely compliant). In addition, objective measures of adherence to the nutrition intervention will be evaluated: urine collected at pre-baseline and the end-of-trial visits will be analysed for the quantification of selected biomarkers of food intake through a target ultra-performance liquid chromatography-ion mobility separation-high resolution mass spectrometry (UPLC-IMS-HRMS) approach at the University of Parma (UNIPR, Italy). In particular, a set of about 150 potential biomarkers of intake related to major food groups (tubers, cereals, legumes, vegetables, fruits, nuts, vegetable oils, dairy products, meat, fish, and alcoholic beverages; among others) and, when available, specific foodstuffs (orange, apple, cocoa, etc.) will be defined on the basis of available literature and the technical feasibility at the time of the sample analysis. Data on biomarkers of food intake will also serve to assess the validity of the information collected through 3-day food diaries at pre-baseline and end-of-trial visits.

Data collection

Data are collected at screening, pre-baseline (V1), baseline (V2), and 10 weeks after initiating the intervention (V3) by using self-reported questionnaires, biological specimens, accelerometers, and physical

For peer review only - http://bmjopen.bmj.com/site/about/guidelines.xhtml

examinations conducted by trained local research staff in accordance with standard operating procedures (Table 5). All primary and secondary endpoints are derived from measurements obtained at baseline (V2) and at the end of the intervention (V3). Measurements taken pre-baseline (V1) were used for clustering assignment. Reminder text messages are being sent to the participants before visit days. It is expected that a high retention rate will be achieved due to the nature of the study.

Primary outcome

The primary endpoint is the difference in body fat mass (kg) from baseline to end of trial between the two intervention groups (personalised vs. control). Body fat mass is determined during body composition analysis by use of dual-energy X-ray absorptiometry (iDXA, Lunar Radiation Co., Madison, Wisconsin, USA). The participants are scanned in a fasted state, lying on their back wearing lightweight clothes without jewellery and other metallic objects.

Secondary outcomes

Anthropometry

Body weight is measured at all visits using a calibrated digital scale to the nearest 0.1 kg with participants wearing lightweight clothes and no shoes, as well as after voiding their bladder. Height is measured at the screening visit using a wall-mounted stadiometer to the nearest 0.5 cm while participants are not wearing shoes. Body mass index (kg/m^2) is calculated as weight in kilograms divided by height in meters squared. Waist circumference (cm) is measured with a stretch-resistant tape at the midpoint between the lower margin of the last palpable ribs and the top of the iliac crest, in the fasted state (non-fasted during the screening visit) with an empty bladder and with participants wearing light clothes. Each measurement is taken twice to the nearest 0.5 cm and the average is used.

Biological Samples

Fasting blood samples collected at pre-baseline, baseline, and week 10 are analysed for plasma glucose, insulin, adipokines (leptin and adiponectin), inflammatory biomarkers (CRP, IL-6, IL-10, TNFα, MCP1, sICAM1, sCD14), lipid profile (total cholesterol, LDL-cholesterol, HDL-cholesterol, oxLDL, triglycerides), liver biomarkers (ALT and GGT), and renal biomarkers (uric acid, creatinine). Blood samples are sent to Eurecat for metabolomics analysis while biochemical markers are measured at NEXS, University of Copenhagen.

For peer review only - http://bmjopen.bmj.com/site/about/guidelines.xhtml

Table 5: Procedures and activities during the study period

	Screening visit (V0)	Pre-baseline visit (V1)	Baseline visit (V2)	End of trial visit (V3)
**Week**	**-20 to -13**	**-9 to -6**	**0**	**10**
**Visit day**	**0**	**1**	**2**	**3**
Informed consent	X			
Review of inclusion and exclusion criteria	X			
Medical history & examination	X			
Randomisation			X[†]	
Registration of medication and adverse events	X	X	X	X
**Anthropometry**				
Body weight	X	X	X	X
Height	X			
Waist circumference	X	X	X	X
Body composition (DXA)			X	X
**Biological Samples**				
Fasting blood sample		X	X	X
Saliva sample (SNPs)		X		
Urine sample		X	X	X
Faecal sample			X*	X*
**Nutritional Assessment**				
Food frequency questionnaire (FFQ)		X		X
3-day dietary records		X*		X*
**Other measurements**				
Blood Pressure/heart rate		X	X	X
Three factor eating questionnaire (TFEQ)			X	X
Perceived stress scale (PSS)			X	X
Behavioural questionnaire (by ONMI)			X	X
Quality of life questionnaires			X	X
Diet satisfaction questionnaire (DSat-28)			X	X
Money spent on food questions			X	X
Accelerometer (sleep and PA)		X**	X**	

†: prior to visit
X*: At home activity prior to visit.
X**: At home activity following the visit.

For peer review only - http://bmjopen.bmj.com/site/about/guidelines.xhtml

Blood pressure

Systolic and diastolic blood pressures and heart rate are measured by an automatic sphygmomanometer on the arm after 5-10 min rest in a sitting position. The same arm is used during all visits. The measurement is repeated three times (or four, if the last two measurements deviate by >5 mmHg), approximately 1 minute apart, and readings are recorded to the nearest 1 mmHg for blood pressures and 1 bpm for heart rate. The average of the last two readings is used.

Other outcome measures

Saliva

Saliva samples were collected pre-baseline and sent to Alimentomica (University of the Balearic Islands, Palma de Mallorca) for analysis of genetic variants, mainly single nucleotide polymorphisms (SNPs) in genes related to metabolism, inflammation, and oxidative stress for cluster assignment. Currently, there are 188 candidate SNPs being analysed and validated in an ongoing trial and a minimum of 35 and maximum of 150 SNPs of those are expected to be used in the present study.

Urine samples

Participants were asked to deliver a second-void urine spot sample in the morning at pre-baseline, and do the same at baseline and end of the study visits. Urine samples from pre-baseline and week 10 are used by UNIPR for food intake biomarker analysis and by Eurecat for analysing markers of oxidative stress utilized in cluster assignment. Furthermore, aliquots of urine samples from all visits are stored in a biobank for future analyses.

Nutritional assessment

Dietary intake is assessed pre-baseline and at the end of the study by using a validated self-administered electronic form of the European Prospective Investigation of Cancer (EPIC)-Norfolk Study food frequency questionnaire (FFQ),[41] supervised by a trained staff member. Furthermore, participants are instructed to complete 3-day weighed food records during the week before the pre-baseline visit and during the third week of the intervention. The dietary records cover two non-consecutive weekdays and one weekend day. Nutrient analysis will be done by the software program Vitakost (Conava ApS; Kolding, Denmark), which is based on the Danish national food database.

For peer review only - http://bmjopen.bmj.com/site/about/guidelines.xhtml

Questionnaires

Eating behaviour assessment at baseline and week 10 is conducted by administrating the three factor-eating questionnaire (TFEQ).[42] This is a 51-item self-report questionnaire which measures three domains of eating behaviour: (1) cognitive restraint of eating, (2) disinhibition and (3) hunger.

Stress assessment is conducted through the 10-item perceived stress scale (PSS)[43] at baseline and week 10. The PSS is one of the most widely used psychological instruments. It measures the degree to which participants perceive events in their life as being stressful by asking about thoughts and feelings over the last month using a response scale from 0 (never) to 4 (very often).

Quality of life is assessed by two questionnaires at baseline and week 10: 1) EQ-5D-5L[44], which is a standardized instrument developed by EuroQol Group for measuring health-related quality of life on five dimensions (mobility, self-care, usual activities, pain/discomfort, and anxiety/depression), with five response levels per dimension, and also includes a visual analogue scale (EQ-VAS) by which respondents report their perceived health status; and 2) Obesity and Weight-Loss Quality of Life Instrument (OWLQOL)[45], which is an instrument consisting of 17 statements about weight-related feelings and emotions which are rated on a seven-point scale, and primarily measures emotions and feelings resulting from suffering from obesity and trying to lose weight.

Diet satisfaction is assessed at baseline and week 10 by the Diet Satisfaction Questionnaire (DSat-28)[46] that involves 28 statements grouped into five dimensions (healthy lifestyle, eating out, cost, preoccupation with food, and planning and preparation) to evaluate satisfaction with weight-management diets.

Food expenditure is assessed by two questions completed at baseline and week 10 regarding the amount of money spent on food for the whole household.

Physical activity and sleep patterns are determined by ActiGraph GT3X+ accelerometer (ActiGraph, LLC, Pensacola, FL, USA) for 7 days/8 nights immediately following the pre-baseline visit and again for 7 days/8 nights during the third week of the intervention. During these wear-periods, a self-administered sleep-log to assess bedtimes is also obtained.

Microbiota composition will be determined on faecal samples collected within 24 hours prior to the clinical investigation days at baseline and week 10. These samples are not used in the cluster assignment but are collected and stored for future analyses.

For peer review only - http://bmjopen.bmj.com/site/about/guidelines.xhtml

Data management

Study investigators have access to the data collection forms and protocols using a secure shared drive. All collected data are pseudo-anonymized and participants are identified only by a study ID number on documents and on electronic databases, with personal identifiers kept separately under strict access control, limited to investigators and study staff directly involved in data collection and entry. All biological specimens sent to the consortium partners are pseudo-anonymized and encrypted. Upon completion of the study, data will be stored in a password-protected database, accessible only by study investigators, in anonymous form for a minimum of 10 years.

Data Monitoring

As the intervention risks to participants are minimal, a Data and Safety Monitoring Board was not deemed necessary. However, in case of unexpected adverse events during the study period, these are recorded and brought up to the principal investigator for appropriate decision-making. We did not plan to conduct interim analysis for safety as we did not anticipate any serious adverse events that would require trial termination.

Power and sample size calculation

To detect a difference in body fat mass change of 1.25 kg between the two intervention groups with 80% power at a two-tailed level of significance of 0.05, assuming a standard deviation (SD) of 2.0 kg, a sample size of 41 per group (i.e., personalised vs. control) is needed, i.e., a total sample size of 82 completers. To allow for an anticipated 18% dropout rate, 50 subjects per group would need to be recruited (total n = 100). The expected difference in fat mass between groups (1.25 kg) and the associated SD (2.0 kg) were based on values calculated from the raw data of the SHOPUS study.[47] In that study, we reported on body weight and fat mass during a 6-month dietary intervention in subjects with BMI of 22.6-47.3 kg/m^2. To conduct power calculations for this study, we extracted raw data from the SHOPUS study that were most representative of the current subjects and intervention duration. Accordingly, we selected those SHOPUS participants with BMI ≥27 kg/m^2 and elevated waist circumference (males >94 cm; females >80 cm) which represented more than 67% of the total study population (n=145) and assessed their body weight and fat mass at 12 weeks; this was an interim time point of the SHOPUS study (not published in the original paper),[47] which was the closest to the 10 week time frame of the current intervention.

For peer review only - http://bmjopen.bmj.com/site/about/guidelines.xhtml

Statistical analysis

Data analysis will be conducted using SPSS and R. Before statistical analyses are conducted, all continuous variables will be tested for normality and homogeneity of variance. For our primary objective, differences in fat mass from baseline to end of trial (V3 minus V2) between the two intervention groups (personalised vs. control) will be compared by means of linear mixed models with time and randomisation group as main effects, a time-by-group interaction, and adjusting for potential confounders (e.g., sex, age, and BMI at baseline) as necessary. If significant interactions emerge, post hoc testing will be used to evaluate effects within the metabolic clusters. LMMs are able to handle possible imbalances between groups in case of missing values in a single response variable.

Ethics and dissemination

The study has been approved by the Regional Committees on Health Research Ethics, Region Hovedstaden in Denmark (H-20029882) and is being conducted in accordance with the Helsinki Declaration. Any protocol amendments are submitted to the Research Ethics Board for approval and communicated to study participants and the trial registry once approved.

All personal data is being handled confidentially and stored in accordance with applicable law, GDPR and Danish Data Protection Agency. Participants received written and oral information on the study procedures, and only trained study-personnel provided information, monitored and attested signing of the informed consent form (online supplemental material 3). In addition, an optional GDPR consent to provide excess sample materials to study biobank was signed. The Research biobank has been approved by the Danish Data Protection Agency.

A manuscript with the results of the primary study will be submitted for publication to an international, peer-reviewed journal, regardless of whether results are positive, negative or inconclusive in relation to the study hypothesis. Authorship eligibility will be based on the recommendations from the International Committee of Medical Journal Editors (ICMJE). Upon completion of the trial, and after publication of the primary manuscript, data requests can be submitted to the principal investigator at the Department of Nutrition, Exercise and Sports (NEXS) at the University of Copenhagen, Denmark.

Perspective

The results from this study will serve as proof of concept for the efficacy of using metabolic and genetic biomarkers to provide personalised diet treatments for reducing body fat mass and subsequently for improving health outcomes, such as metabolic and inflammatory markers, in high-risk individuals. Moreover, the findings will inform recommendations regarding the efficacy of using a web-based decision support system for personalising dietary plans to support and maintain health-promoting behaviours.

For peer review only - http://bmjopen.bmj.com/site/about/guidelines.xhtml

Contributors

The overall framework of the EU-project PREVENTOMICS was initiated by BG, ANC and JMDB. The overall design of the present Danish study involved all co-authors. Detailed planning, implementation and daily management of the Danish study was carried out by KP, MAA, FM and MFH. MAA, FM and MFH drafted the initial manuscript. KP, SMOG, PM, FS, MP, MW, ALC, JMDB, BG critically reviewed and edited the manuscript. All authors approved the final version for publication.

Trial sponsor: University of Copenhagen

Contact name: Faidon Magkos

Email: fma@nexs.ku.dk

Address: 1958 Frederiksberg C, Denmark

Funding: This work and the European partners in the PREVENTOMICS project are supported by the European Union’s Horizon 2020 research and innovation program under grant agreement No. 818318.

Competing interests: None declared.

Patient consent for publication: Not required.

Provenance and peer review: Not commissioned; externally peer reviewed

Ethics approval: The study is approved by the Scientific Ethics Committee Region H in Denmark with journal number H-20029882, and registered at Clinicaltrials.gov with: NCT04590989.

Figure 1: Study design and timeline. *The results for metabolome and genotype analyses are integrated into the PREVENTOMICS platform. SF: Simple feast.

Figure 2: Schematic diagram of the intervention. CARB: carbohydrate cluster, LIPID: lipid cluster, INFL: inflammation cluster, OXIS: oxidative stress cluster, MB: microbiota cluster.

For peer review only

References:

1. Ohlhorst SD, Russell R, Bier D, et al. Nutrition research to affect food and a healthy lifespan. *Adv Nutr* 2013;4(5):579-84.
2. Singhal A. The global epidemic of noncommunicable disease: the role of early-life factors. *Nestle Nutr Inst Workshop Ser* 2014;78:123-32.
3. Hruby A, Hu FB. The Epidemiology of Obesity: A Big Picture. *Pharmacoeconomics* 2015;33(7):673-89.
4. Nyberg ST, Batty GD, Pentti J, et al. Obesity and loss of disease-free years owing to major non-communicable diseases: a multicohort study. *Lancet Public Health* 2018;3(10):e490-e97.
5. Sacks FM, Bray GA, Carey VJ, et al. Comparison of weight-loss diets with different compositions of fat, protein, and carbohydrates. *N Engl J Med* 2009;360(9):859-73.
6. Shai I, Schwarzfuchs D, Henkin Y, et al. Weight loss with a low-carbohydrate, Mediterranean, or low-fat diet. *N Engl J Med* 2008;359(3):229-41.
7. Stanton MV, Robinson JL, Kirkpatrick SM, et al. DIETFITS study (diet intervention examining the factors interacting with treatment success) - Study design and methods. *Contemp Clin Trials* 2017;53:151-61.
8. Hjorth MF, Corella D, Astrup A, et al. High fat diets for weight loss among subjects with elevated fasting glucose levels: The PREDIMED study. *Obesity Medicine* 2020;18:100210.
9. Aleksandrova K, Egea Rodrigues C, Floegel A, et al. Omics Biomarkers in Obesity: Novel Etiological Insights and Targets for Precision Prevention. *Curr Obes Rep* 2020;9(3):219-30.
10. Verma M, Hontecillas R, Tubau-Juni N, et al. Challenges in Personalized Nutrition and Health. *Front Nutr* 2018;5:117.
11. Odriozola L, Corrales FJ. Discovery of nutritional biomarkers: future directions based on omics technologies. *Int J Food Sci Nutr* 2015;66 Suppl 1:S31-40.
12. Chan AW, Tetzlaff JM, Altman DG, et al. SPIRIT 2013 statement: defining standard protocol items for clinical trials. *Ann Intern Med* 2013;158(3):200-7.
13. Aulchenko YS, Ripatti S, Lindqvist I, et al. Loci influencing lipid levels and coronary heart disease risk in 16 European population cohorts. *Nat Genet* 2009;41(1):47-55.
14. Dastani Z, Hivert MF, Timpson N, et al. Novel loci for adiponectin levels and their influence on type 2 diabetes and metabolic traits: a multi-ethnic meta-analysis of 45,891 individuals. *PLoS Genet* 2012;8(3):e1002607.
15. Draisma HHM, Pool R, Kobl M, et al. Genome-wide association study identifies novel genetic variants contributing to variation in blood metabolite levels. *Nat Commun* 2015;6:7208.

For peer review only - http://bmjopen.bmj.com/site/about/guidelines.xhtml

16. Guan W, Steffen BT, Lemaitre RN, et al. Genome-wide association study of plasma N6 polyunsaturated fatty acids within the cohorts for heart and aging research in genomic epidemiology consortium. *Circ Cardiovasc Genet* 2014;7(3):321-31.

17. Hu Y, Liu W, Huang R, et al. A systematic review and meta-analysis of the relationship between lipoprotein lipase Asn291Ser variant and diseases. *J Lipid Res* 2006;47(9):1908-14.

18. Kathiresan S, Willer CJ, Peloso GM, et al. Common variants at 30 loci contribute to polygenic dyslipidemia. *Nat Genet* 2009;41(1):56-65.

19. Kilpeläinen TO, Carli JF, Skowronski AA, et al. Genome-wide meta-analysis uncovers novel loci influencing circulating leptin levels. *Nat Commun* 2016;7:10494.

20. Tintle NL, Pottala JV, Lacey S, et al. A genome-wide association study of saturated, mono- and polyunsaturated red blood cell fatty acids in the Framingham Heart Offspring Study. *Prostaglandins Leukot Essent Fatty Acids* 2015;94:65-72.

21. Dupuis J, Langenberg C, Prokopenko I, et al. New genetic loci implicated in fasting glucose homeostasis and their impact on type 2 diabetes risk. *Nat Genet* 2010;42(2):105-16.

22. Mahajan A, Sim X, Ng HJ, et al. Identification and functional characterization of G6PC2 coding variants influencing glycemic traits define an effector transcript at the G6PC2-ABCB11 locus. *PLoS Genet* 2015;11(1):e1004876.

23. Manning AK, Hivert MF, Scott RA, et al. A genome-wide approach accounting for body mass index identifies genetic variants influencing fasting glycemic traits and insulin resistance. *Nat Genet* 2012;44(6):659-69.

24. Shin SY, Fauman EB, Petersen AK, et al. An atlas of genetic influences on human blood metabolites. *Nat Genet* 2014;46(6):543-50.

25. Ahola-Olli AV, Würtz P, Havulinna AS, et al. Genome-wide Association Study Identifies 27 Loci Influencing Concentrations of Circulating Cytokines and Growth Factors. *Am J Hum Genet* 2017;100(1):40-50.

26. Ligthart S, Vaez A, Võsa U, et al. Genome Analyses of >200,000 Individuals Identify 58 Loci for Chronic Inflammation and Highlight Pathways that Link Inflammation and Complex Disorders. *Am J Hum Genet* 2018;103(5):691-706.

27. Ljungman P, Bellander T, Nyberg F, et al. DNA variants, plasma levels and variability of interleukin-6 in myocardial infarction survivors: results from the AIRGENE study. *Thromb Res* 2009;124(1):57-64.

28. Qi L, van Dam RM, Meigs JB, et al. Genetic variation in IL6 gene and type 2 diabetes: tagging-SNP haplotype analysis in large-scale case-control study and meta-analysis. *Hum Mol Genet* 2006;15(11):1914-20.

For peer review only - http://bmjopen.bmj.com/site/about/guidelines.xhtml

29. Galmés S, Cifre M, Palou A, et al. A Genetic Score of Predisposition to Low-Grade Inflammation Associated with Obesity May Contribute to Discern Population at Risk for Metabolic Syndrome. *Nutrients* 2019;11(2)
30. Jerotic D, Matic M, Suvakov S, et al. Association of Nrf2, SOD2 and GPX1 Polymorphisms with Biomarkers of Oxidative Distress and Survival in End-Stage Renal Disease Patients. *Toxins (Basel)* 2019;11(7)
31. van Meurs JB, Pare G, Schwartz SM, et al. Common genetic loci influencing plasma homocysteine concentrations and their effect on risk of coronary artery disease. *Am J Clin Nutr* 2013;98(3):668-76.
32. Jia Q, Han Y, Huang P, et al. Genetic Determinants of Circulating Glycine Levels and Risk of Coronary Artery Disease. *J Am Heart Assoc* 2019;8(10):e011922.
33. Wittemans LBL, Lotta LA, Oliver-Williams C, et al. Assessing the causal association of glycine with risk of cardio-metabolic diseases. *Nat Commun* 2019;10(1):1060.
34. Hernandez-Baixauli J, Quesada-Vázquez S, Mariné-Casadó R, et al. Detection of Early Disease Risk Factors Associated with Metabolic Syndrome: A New Era with the NMR Metabolomics Assessment. *Nutrients* 2020;12(3)
35. Nordic Nutrition Recommendations 2012 : Integrating nutrition and physical activity. 5 ed. Copenhagen: Nordisk Ministerråd 2014.
36. Salmon J. Excerpts from Dietary Reference Values for Food Energy and Nutrients for the United Kingdom: introduction to the Guide and Summary Tables. *Nutr Rev* 1992;50(3):90-3.
37. Hervik AK, Svihus B. The Role of Fiber in Energy Balance. *Journal of Nutrition and Metabolism* 2019;2019:4983657.
38. Wanders AJ, van den Borne JJ, de Graaf C, et al. Effects of dietary fibre on subjective appetite, energy intake and body weight: a systematic review of randomized controlled trials. *Obes Rev* 2011;12(9):724-39.
39. Papathanasopoulos A, Camilleri M. Dietary fiber supplements: effects in obesity and metabolic syndrome and relationship to gastrointestinal functions. *Gastroenterology* 2010;138(1):65-72.e1-2.
40. Habibović M, Broers E, Piera-Jimenez J, et al. Enhancing Lifestyle Change in Cardiac Patients Through the Do CHANGE System ("Do Cardiac Health: Advanced New Generation Ecosystem"): Randomized Controlled Trial Protocol. *JMIR Res Protoc* 2018;7(2):e40.
41. Bingham SA, Gill C, Welch A, et al. Validation of dietary assessment methods in the UK arm of EPIC using weighed records, and 24-hour urinary nitrogen and potassium and serum vitamin C and carotenoids as biomarkers. *Int J Epidemiol* 1997;26 Suppl 1:S137-51.

For peer review only - http://bmjopen.bmj.com/site/about/guidelines.xhtml

42. Stunkard AJ, Messick S. The three-factor eating questionnaire to measure dietary restraint, disinhibition and hunger. *J Psychosom Res* 1985;29(1):71-83.

43. Cohen S, Kamarck T, Mermelstein R. A global measure of perceived stress. *J Health Soc Behav* 1983;24(4):385-96.

44. EuroQol--a new facility for the measurement of health-related quality of life. *Health Policy* 1990;16(3):199-208.

45. Niero M, Martin M, Finger T, et al. A new approach to multicultural item generation in the development of two obesity-specific measures: the Obesity and Weight Loss Quality of Life (OWLQOL) questionnaire and the Weight-Related Symptom Measure (WRSM). *Clin Ther* 2002;24(4):690-700.

46. James BL, Loken E, Roe LS, et al. Validation of the Diet Satisfaction Questionnaire: a new measure of satisfaction with diets for weight management. *Obes Sci Pract* 2018;4(6):506-14.

47. Poulsen SK, Due A, Jordy AB, et al. Health effect of the New Nordic Diet in adults with increased waist circumference: a 6-mo randomized controlled trial. *Am J Clin Nutr* 2014;99(1):35-45.

For peer review only

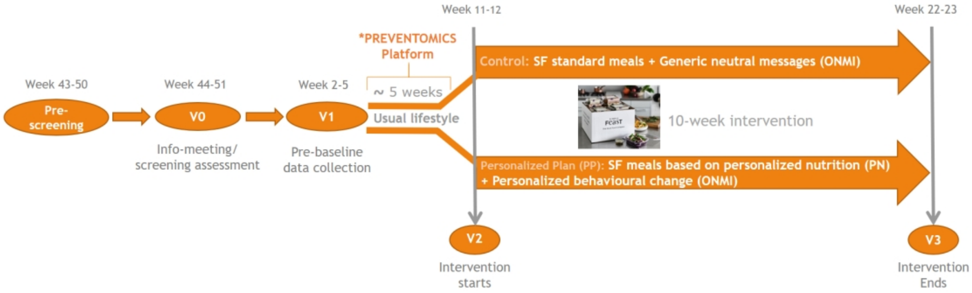

Figure 1: Study design and timeline. *The analysis results on subjects' metabolome and genotypes will be integrated into PREVENTOMICS platform. This takes about 5 weeks from the date of sending samples from the University of Copenhagen to the assigned partners (Eurecat, Alimentomica). SF, Simple Feast.

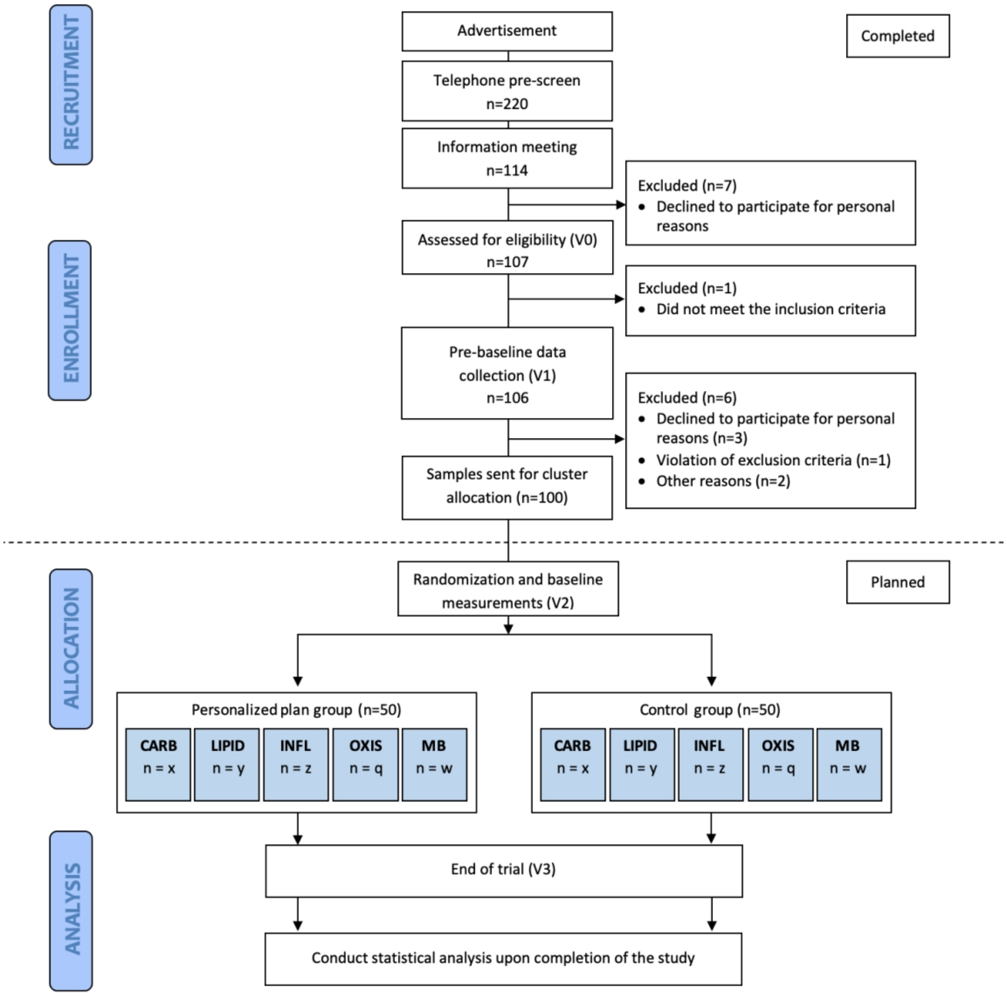

Figure 2: Schematic diagram of the intervention. CARB, carbohydrate cluster; LIPID, lipid cluster; INFL, inflammation cluster; OXIS, oxidative stress cluster; MB, microbiota cluster.

SUPPLEMENTARY ONLINE MATERIAL 1

Table 1: Biomarkers included in the carbohydrate cluster

**(1) Carbohydrates cluster**		
**Biomarker**	**Bio-fluid**	**Reference**
**Glucose**	Plasma	Cosentino et al., 2020[1]
**HOMA-IR**	-	Bloomgarden, 2003[2]<br>Govers et al., 2015[3]<br>Shashaj et al., 2016[4]
**Insulin**	Serum	Aleksandrova et al., 2018[5]<br>Bloomgarden, 2003[2]<br>Govers et al., 2015[3]
**Leptin**	Serum	Chen et al., 2014[6]<br>Finucane et al., 2019[7]<br>López-Jaramillo et al., 2014[8]
**Adiponectin**	Serum	Dastani et al., 2012[9]<br>Li et al., 2009[10]<br>Liu et al., 2016[11]<br>Wang et al., 2018[12]
**Lactate**	Serum	Berhane et al., 2015[13]<br>Choi et al., 2002[14]<br>Lovejoy et al., 1992[15]<br>Shantha et al., 2013[16]
**Glutamate**	Serum	Martin and Price, 2018[17]<br>Ottosson et al., 2018[18]
**Uric acid**	Serum	Darmawan et al., 2018[19]<br>Fabbrini et al., 2014[20]<br>Johnson et al., 2013[21]<br>van der Schaft et al., 2017[22]
**Propionylcarnitine**	Plasma	Mai et al., 2013[23]<br>Mihalik et al., 2010[24]<br>Zhang et al., 2014[25]
**BCAA (Valine, Leucine, Isoleucine)**	Serum	Chen et al., 2016[26]<br>Katagiri et al., 2018[27]<br>Lotta et al., 2016[28]<br>Newgard et al., 2009[29]<br>Okekunle et al., 2019[30]
**Phenylalanine**	Serum	Chen et al., 2019[31]<br>Suzuki et al., 2019[32]<br>Wang et al., 2011[33]
**Tyrosine**	Serum	Chen et al., 2019[31]<br>Hellmuth et al., 2016[34]<br>Wang et al., 2011[33]
**Glutamine**	Serum	Chen et al., 2019[31]<br>Guasch-Ferré et al., 2016[35]<br>Liu et al., 2019[36]<br>Rhee et al., 2018[37]

Abbreviations: HOMA-IR, homeostatic model assessment of insulin resistance; BCAA, branched chain amino acids.

For peer review only

For peer review only - http://bmjopen.bmj.com/site/about/guidelines.xhtml

Table 2: Biomarkers included in the lipid cluster

**(2) Lipid cluster**		
**Biomarker**	**Bio-fluid**	**Reference**
**LDL-cholesterol**	Plasma	Grundy et al., 2019[38]
**HDL-cholesterol**	Plasma	Grundy et al., 2019[38]
**Triglycerides**	Plasma	Grundy et al., 2019[38]
**Total cholesterol**	Plasma	Grundy et al., 2019[38]
**PUFAs (total)**	Serum	Koga et al., 2019[39]
**LPCs (total)**	Plasma	Law et al., 2019[40]
**Linoleic acid C18:2**	Serum	Koga et al., 2019[39]
**Oleic acid C18:1**	Serum	Griel and Kris-Etherton, 2006[41]<br>Yamagishi et al., 2013[42]
**Leptin**	Serum	Mantzoros and Flier, 2000[43]
**Adiponectin**	Serum	Abdella and Mojiminiyi, 2018[44]<br>Liu et al., 2018[45]
**SFAs**	Serum	Liu et al., 2019[46]
**3-hydroxybutyrate**	Serum	Margolis and O'Fallon, 2020[47]
**MUFAs (total)**	Serum	Griel and Kris-Etherton, 2006[41]<br>Yamagishi et al., 2013[42]
**Propionylcarnitine**	Plasma	Ottosson et al., 2018[18]
**DHA C20:3**	Serum	Koga et al., 2019[39]
**Choline**	Serum	Ottosson et al., 2018[18]

Abbreviations: LDL, low-density lipoprotein; HDL, high-density lipoprotein; PUFAs, poly unsaturated fatty acids; LPC, lysophosphatidylcholine; SFAs, saturated fatty acids; MUFAs, monounsaturated fatty acids; DHA, docosahexaenoic acid.

For peer review only

For peer review only - http://bmjopen.bmj.com/site/about/guidelines.xhtml

Table 3: Biomarkers included in the inflammation cluster

**(3) Inflammation cluster**		
**Biomarker**	**Bio-fluid**	**Reference**
**CRP**	Serum	Amsterdam, 2003[48]<br>Knight, 2015[49]<br>Liu et al., 2016[11]<br>Musunuru et al., 2008[50]<br>Wang et el., 2013[51]
**IL-6**	Plasma	Liu et al., 2016[11]<br>Wang et al., 2013[51]
**N-acetylglycoproteins**	Serum	Gruppen et al., 2019[52]<br>Ritchie et al., 2015[53]
**TNFα**	Plasma	Liu et al., 2016[11]
**MCP-1**	Plasma	Georgakis et al., 2019[54]
**IL-10**	Plasma	Charles et al., 2011[55]<br>Leon-Cabrera et al., 2015[56]<br>Meng et al., 2019[57]
**sICAM1**	Plasma	El Amine et al., 2010[58]<br>Luc et al., 2003[59]<br>Straczkowski et al., 2002[60]
**LBP**	Plasma	Moreno-Navarrete et al., 2012[61]
**sCD14**	Plasma	de Courten et al., 2016[62]
**LPCs (total)**	Plasma	Iwase et al., 2008[63]
**Linoleic acid C18:2**	Serum	Steffen et al., 2012[64]<br>Yli-Jama et al., 2002[65]
**DHA C20:3**	Serum	Steffen et al., 2012[64]<br>Yli-Jama et al., 2002[65]

Abbreviations: CRP, C-reactive protein; IL-6, interleukin-6; IL-10, interleukin-10; TNFα, tumor necrosis factor alpha; MCP-1, monocyte chemoattractant protein-1; sICAM-1, soluble intercellular adhesion molecule-1; LBP, lipopolysaccharide binding protein; sCD14, soluble CD14; LPC, lysophosphatidylcholines; DHA: docosahexaenoic acid.

For peer review only

Table 4: Biomarkers included in the oxidative stress cluster

**(4) Oxidative stress cluster**		
**Biomarker**	**Bio-fluid**	**Reference**
**8-iso-PGF2α**	Urine	Davies and Roberts, 2011[66]<br>Kim et al., 2012[67]<br>Milne et al., 2015[68]<br>van't Erve, 2018[69]
**8-OHdG**	Urine	Di Minno et al., 2016[70]<br>Kroese and Scheffe, 2014[71]
**LDLox**	Plasma	Barbosa et al., 2011[72]<br>Gao et al., 2017[73]
**Uric acid**	Serum	Darmawan et al., 2018[19]<br>Fabbrini et al., 2014[20]<br>van der Schaft et al., 2017[22]
**Allantoin**	Urine	Il'yasova et al., 2012[74]
**Betaine**	Urine	Svingen et al., 2016[75]<br>Walford et al., 2016[76]
**Pseudouridine**	Urine	Topp et al., 2008[77]
**Dimethylglycine**	Urine	Svingen et al., 2016[75]
**Glycine**	Serum	Sekhar et al., 2011[78]
**Methionine**	Serum	Grizales et al., 2018[79]

Abbreviations: 8-iso-PGF2α, 8-iso-prostaglandin F2 alpha; 8-OHdG, 8-hydroxydeoxyguanosine; LDLox, oxidized low density lipoprotein.

For peer review only

For peer review only - http://bmjopen.bmj.com/site/about/guidelines.xhtml

Table 5: Biomarkers included in the microbiota cluster

**(5) Microbiota cluster**		
**Biomarker**	**Bio-fluid**	**Reference**
**TMAO**	Serum	Bain et al., 2006[80]<br>Chen et al., 2016[81]<br>Ge et al., 2020[82]<br>Heianza et al., 2017[83]<br>Schiattarella et al., 2017[84]<br>Yang et el., 2019[85]<br>Yao et al., 2020[86]
**TMA**	Urine	Aragonès et al., 2019[87]<br>Bouatra et al., 2013[88]<br>Chen et al., 2016[81]<br>Palau-Rodriguez et al., 2015[89]<br>Yang et el., 2019[85]
**DMA**	Urine	Bouatra et al., 2013[88]<br>Palau-Rodriguez et al., 2015[89]
**Betaine**	Urine	Chen et al., 2016[81]<br>Palau-Rodriguez et al., 2015[89]<br>Yang et el., 2019[85]
**Choline**	Serum	Aragonès et al., 2019[87]<br>Chen et al., 2016[81]<br>Palau-Rodriguez et al., 2015[89]<br>Yang et el., 2019[85]
**Dimethylglycine**	Urine	Palau-Rodriguez et al., 2015[89]
**Succinate**	Plasma	Psychogios et al., 2011[90]<br>Serena et al., 2018[91]
**Lactate**	Serum	Psychogios et al., 2011[90]<br>Vojinovic et al., 2019[92]
**LBP**	Plasma	Awoyemi et al., 2018[93]<br>Barengolts et al., 2019[94]<br>Bouatra et al., 2013[88]<br>Citronberg et al., 2018[95]
**Acetate**	Urine	Liu et al., 2014[96]

Abbreviations: TMAO, trimethylamine N-oxide; TMA, trimethylamine; DMA, dimethylamine; LBP, lipopolysaccharide binding protein.

For peer review only

For peer review only - http://bmjopen.bmj.com/site/about/guidelines.xhtml

References:

1. Cosentino F, Grant PJ, Aboyans V, et al. 2019 ESC Guidelines on diabetes, pre-diabetes, and cardiovascular diseases developed in collaboration with the EASD. *Eur Heart J* 2020;41(2):255-323.
2. Bloomgarden ZT. American Association of Clinical Endocrinologists (AACE) consensus conference on the insulin resistance syndrome: 25-26 August 2002, Washington, DC. *Diabetes Care* 2003;26(4):1297-303.
3. Govers E, Slof E, Verkoelen H, et al. Guideline for the Management of Insulin Resistance. *Int J Endocrinol Metab Disord* 2015;1(4).
4. Shashaj B, Luciano R, Contoli B, et al. Reference ranges of HOMA-IR in normal-weight and obese young Caucasians. *Acta Diabetol* 2016;53(2):251-60.
5. Aleksandrova K, Mozaffarian D, Pischon T. Addressing the Perfect Storm: Biomarkers in Obesity and Pathophysiology of Cardiometabolic Risk. *Clin Chem* 2018;64(1):142-53.
6. Chen GC, Qin LQ, Ye JK. Leptin levels and risk of type 2 diabetes: gender-specific meta-analysis. *Obes Rev* 2014;15(2):134-42.
7. Finucane FM, Luan J, Wareham NJ, et al. Correlation of the leptin:adiponectin ratio with measures of insulin resistance in non-diabetic individuals. *Diabetologia* 2009;52(11):2345-49.
8. López-Jaramillo P, Gómez-Arbeláez D, López-López J, et al. The role of leptin/adiponectin ratio in metabolic syndrome and diabetes. *Horm Mol Biol Clin Investig* 2014;18(1):37-45.
9. Dastani Z, Hivert MF, Timpson N, et al. Novel loci for adiponectin levels and their influence on type 2 diabetes and metabolic traits: a multi-ethnic meta-analysis of 45,891 individuals. *PLoS Genet* 2012;8(3):e1002607.
10. Li S, Shin HJ, Ding EL, et al. Adiponectin levels and risk of type 2 diabetes: a systematic review and meta-analysis. *Jama* 2009;302(2):179-88.
11. Liu C, Feng X, Li Q, et al. Adiponectin, TNF-α and inflammatory cytokines and risk of type 2 diabetes: A systematic review and meta-analysis. *Cytokine* 2016;86:100-09.
12. Wang Y, Meng RW, Kunutsor SK, et al. Plasma adiponectin levels and type 2 diabetes risk: a nested case-control study in a Chinese population and an updated meta-analysis. *Sci Rep* 2018;8(1):406.
13. Berhane F, Fite A, Daboul N, et al. Plasma Lactate Levels Increase during Hyperinsulinemic Euglycemic Clamp and Oral Glucose Tolerance Test. *J Diabetes Res* 2015;2015:102054.
14. Choi CS, Kim YB, Lee FN, et al. Lactate induces insulin resistance in skeletal muscle by suppressing glycolysis and impairing insulin signaling. *Am J Physiol Endocrinol Metab* 2002;283(2):E233-40.
15. Lovejoy J, Newby FD, Gebhart SSP, et al. Insulin resistance in obesity is associated with elevated basal lactate levels and diminished lactate appearance following intravenous glucose and insulin. *Metabolism* 1992;41(1):22-27.
16. Shantha GP, Wasserman B, Astor BC, et al. Association of blood lactate with carotid atherosclerosis: the Atherosclerosis Risk in Communities (ARIC) Carotid MRI Study. *Atherosclerosis* 2013;228(1):249-55.
17. Martin AJ, Price CI. A Systematic Review and Meta-Analysis of Molecular Biomarkers Associated with Early Neurological Deterioration Following Acute Stroke. *Cerebrovasc Dis* 2018;46(5-6):230-41.
18. Ottosson F, Brunkwall L, Ericson U, et al. Connection Between BMI-Related Plasma Metabolite Profile and Gut Microbiota. *J Clin Endocrinol Metab* 2018;103(4):1491-501.
19. Darmawan G, Hamijoyo L, Hasan I. Association between Serum Uric Acid and Non-Alcoholic Fatty Liver Disease: A Meta-Analysis. *Acta Med Indones* 2017;49(2):136-47.
20. Fabbrini E, Serafini M, Colic Baric I, et al. Effect of plasma uric acid on antioxidant capacity, oxidative stress, and insulin sensitivity in obese subjects. *Diabetes* 2014;63(3):976-81.
21. Johnson RJ, Nakagawa T, Sanchez-Lozada LG, et al. Sugar, uric acid, and the etiology of diabetes and obesity. *Diabetes* 2013;62(10):3307-15.
22. van der Schaft N, Brahimaj A, Wen KX, et al. The association between serum uric acid and the incidence of prediabetes and type 2 diabetes mellitus: The Rotterdam Study. *PLoS One* 2017;12(6):e0179482.
23. Mai M, Tönjes A, Kovacs P, et al. Serum levels of acylcarnitines are altered in prediabetic conditions. *PLoS One* 2013;8(12):e82459.
24. Mihalik SJ, Goodpaster BH, Kelley DE, et al. Increased levels of plasma acylcarnitines in obesity and type 2 diabetes and identification of a marker of glucolipotoxicity. *Obesity (Silver Spring)* 2010;18(9):1695-700.
25. Zhang X, Zhang C, Chen L, et al. Human serum acylcarnitine profiles in different glucose tolerance states. *Diabetes Res Clin Pract* 2014;104(3):376-82.
26. Chen T, Ni Y, Ma X, et al. Branched-chain and aromatic amino acid profiles and diabetes risk in Chinese populations. *Sci Rep* 2016;6:20594.
27. Katagiri R, Goto A, Budhathoki S, et al. Association between plasma concentrations of branched-chain amino acids and adipokines in Japanese adults without diabetes. *Sci Rep* 2018;8(1):1043.

For peer review only - http://bmjopen.bmj.com/site/about/guidelines.xhtml

28. Lotta LA, Scott RA, Sharp SJ, et al. Genetic Predisposition to an Impaired Metabolism of the Branched-Chain Amino Acids and Risk of Type 2 Diabetes: A Mendelian Randomisation Analysis. *PLoS Med* 2016;13(11):e1002179.
29. Newgard CB, An J, Bain JR, et al. A branched-chain amino acid-related metabolic signature that differentiates obese and lean humans and contributes to insulin resistance. *Cell Metab* 2009;9(4):311-26.
30. Okekunle AP, Zhang M, Wang Z, et al. Dietary branched-chain amino acids intake exhibited a different relationship with type 2 diabetes and obesity risk: a meta-analysis. *Acta Diabetol* 2019;56(2):187-95.
31. Chen S, Akter S, Kuwahara K, et al. Serum amino acid profiles and risk of type 2 diabetes among Japanese adults in the Hitachi Health Study. *Sci Rep* 2019;9(1):7010.
32. Suzuki Y, Kido J, Matsumoto S, et al. Associations among amino acid, lipid, and glucose metabolic profiles in childhood obesity. *BMC Pediatr* 2019;19(1):273.
33. Wang TJ, Larson MG, Vasan RS, et al. Metabolite profiles and the risk of developing diabetes. *Nat Med* 2011;17(4):448-53.
34. Hellmuth C, Kirchberg FF, Lass N, et al. Tyrosine Is Associated with Insulin Resistance in Longitudinal Metabolomic Profiling of Obese Children. *J Diabetes Res* 2016;2016:2108909.
35. Guasch-Ferré M, Hruby A, Toledo E, et al. Metabolomics in Prediabetes and Diabetes: A Systematic Review and Meta-analysis. *Diabetes Care* 2016;39(5):833-46.
36. Liu X, Zheng Y, Guasch-Ferré M, et al. High plasma glutamate and low glutamine-to-glutamate ratio are associated with type 2 diabetes: Case-cohort study within the PREDIMED trial. *Nutr Metab Cardiovasc Dis* 2019;29(10):1040-49.
37. Rhee SY, Jung ES, Park HM, et al. Plasma glutamine and glutamic acid are potential biomarkers for predicting diabetic retinopathy. *Metabolomics* 2018;14(7):89.
38. Grundy SM, Stone NJ, Bailey AL, et al. 2018 AHA/ACC/AACVPR/AAPA/ABC/ACPM/ADA/AGS/APhA/ASPC/NLA/PCNA Guideline on the Management of Blood Cholesterol: A Report of the American College of Cardiology/American Heart Association Task Force on Clinical Practice Guidelines. *J Am Coll Cardiol* 2019;73(24):e285-e350.
39. Koga N, Ogura J, Yoshida F, et al. Altered polyunsaturated fatty acid levels in relation to proinflammatory cytokines, fatty acid desaturase genotype, and diet in bipolar disorder. *Transl Psychiatry* 2019;9(1):208.
40. Law SH, Chan ML, Marathe GK, et al. An Updated Review of Lysophosphatidylcholine Metabolism in Human Diseases. *Int J Mol Sci* 2019;20(5).
41. Griel AE, Kris-Etherton PM. Tree nuts and the lipid profile: a review of clinical studies. *Br J Nutr* 2006;96 Suppl 2:S68-78.
42. Yamagishi K, Folsom AR, Steffen LM. Plasma fatty acid composition and incident ischemic stroke in middle-aged adults: the Atherosclerosis Risk in Communities (ARIC) Study. *Cerebrovasc Dis* 2013;36(1):38-46.
43. Mantzoros CS, Flier JS. Editorial: leptin as a therapeutic agent--trials and tribulations. *J Clin Endocrinol Metab* 2000;85(11):4000-2.
44. Abdella NA, Mojiminiyi OA. Clinical Applications of Adiponectin Measurements in Type 2 Diabetes Mellitus: Screening, Diagnosis, and Marker of Diabetes Control. *Dis Markers* 2018;2018:5187940.
45. Liu Z, Liang S, Que S, et al. Meta-Analysis of Adiponectin as a Biomarker for the Detection of Metabolic Syndrome. *Front Physiol* 2018;9:1238.
46. Liu Q, Matthan NR, Manson JE, et al. Plasma Phospholipid Fatty Acids and Coronary Heart Disease Risk: A Matched Case-Control Study within the Women's Health Initiative Observational Study. *Nutrients* 2019;11(7).
47. Margolis LM, O'Fallon KS. Utility of Ketone Supplementation to Enhance Physical Performance: A Systematic Review. *Adv Nutr* 2020;11(2):412-19.
48. Amsterdam EA. C-reactive protein: a guideline for its application. *Prev Cardiol* 2003;6(2):70.
49. Knight ML. The Application of High-Sensitivity C-Reactive Protein in Clinical Practice: A 2015 Update. *US Pharm* 2015;40(2):50-53.
50. Musunuru K, Kral BG, Blumenthal RS, et al. The use of high-sensitivity assays for C-reactive protein in clinical practice. *Nat Clin Pract Cardiovasc Med* 2008;5(10):621-35.
51. Wang X, Bao W, Liu J, et al. Inflammatory markers and risk of type 2 diabetes: a systematic review and meta-analysis. *Diabetes Care* 2013;36(1):166-75.
52. Gruppen EG, Kunutsor SK, Kieneker LM, et al. GlycA, a novel pro-inflammatory glycoprotein biomarker is associated with mortality: results from the PREVEND study and meta-analysis. *J Intern Med* 2019;286(5):596-609.
53. Ritchie SC, Würtz P, Nath AP, et al. The Biomarker GlycA Is Associated with Chronic Inflammation and Predicts Long-Term Risk of Severe Infection. *Cell Syst* 2015;1(4):293-301.

For peer review only - http://bmjopen.bmj.com/site/about/guidelines.xhtml

54. Georgakis MK, Malik R, Björkbacka H, et al. Circulating Monocyte Chemoattractant Protein-1 and Risk of Stroke: Meta-Analysis of Population-Based Studies Involving 17 180 Individuals. *Circ Res* 2019;125(8):773-82.
55. Charles BA, Doumatey A, Huang H, et al. The roles of IL-6, IL-10, and IL-1RA in obesity and insulin resistance in African-Americans. *J Clin Endocrinol Metab* 2011;96(12):E2018-22.
56. Leon-Cabrera S, Arana-Lechuga Y, Esqueda-León E, et al. Reduced systemic levels of IL-10 are associated with the severity of obstructive sleep apnea and insulin resistance in morbidly obese humans. *Mediators Inflamm* 2015;2015:493409.
57. Meng D, Liang L, Guo X. Serum interleukin-10 level in patients with inflammatory bowel disease: A meta-analysis. *Eur J Inflamm* 2019;17:1-7.
58. El Amine M, Sohawon S, Lagneau L, et al. Plasma levels of ICAM-1 and circulating endothelial cells are elevated in unstable types 1 and 2 diabetes. *Endocr Regul* 2010;44(1):17-24.
59. Luc G, Arveiler D, Evans A, et al. Circulating soluble adhesion molecules ICAM-1 and VCAM-1 and incident coronary heart disease: the PRIME Study. *Atherosclerosis* 2003;170(1):169-76.
60. Straczkowski M, Lewczuk P, Dzienis-Straczkowska S, et al. Elevated soluble intercellular adhesion molecule-1 levels in obesity: relationship to insulin resistance and tumor necrosis factor-alpha system activity. *Metabolism* 2002;51(1):75-8.
61. Moreno-Navarrete JM, Ortega F, Serino M, et al. Circulating lipopolysaccharide-binding protein (LBP) as a marker of obesity-related insulin resistance. *Int J Obes (Lond)* 2012;36(11):1442-9.
62. de Courten B, Moreno-Navarrete JM, Lyons J, et al. Contrasting association of circulating sCD14 with insulin sensitivity in non-obese and morbidly obese subjects. *Mol Nutr Food Res* 2016;60(1):103-9.
63. Iwase M, Sonoki K, Sasaki N, et al. Lysophosphatidylcholine contents in plasma LDL in patients with type 2 diabetes mellitus: relation with lipoprotein-associated phospholipase A2 and effects of simvastatin treatment. *Atherosclerosis* 2008;196(2):931-6.
64. Steffen BT, Steffen LM, Tracy R, et al. Obesity modifies the association between plasma phospholipid polyunsaturated fatty acids and markers of inflammation: the Multi-Ethnic Study of Atherosclerosis. *Int J Obes (Lond)* 2012;36(6):797-804.
65. Yli-Jama P, Seljeflot I, Meyer HE, et al. Serum non-esterified very long-chain PUFA are associated with markers of endothelial dysfunction. *Atherosclerosis* 2002;164(2):275-81.
66. Davies SS, Roberts LJ, 2nd. F2-isoprostanes as an indicator and risk factor for coronary heart disease. *Free Radic Biol Med* 2011;50(5):559-66.
67. Kim JY, Lee JW, Youn YJ, et al. Urinary levels of 8-iso-prostaglandin f2α and 8-hydroxydeoxyguanine as markers of oxidative stress in patients with coronary artery disease. *Korean Circ J* 2012;42(9):614-7.
68. Milne GL, Dai Q, Roberts LJ, 2nd. The isoprostanes--25 years later. *Biochim Biophys Acta* 2015;1851(4):433-45.
69. van 't Erve TJ. Strategies to decrease oxidative stress biomarker levels in human medical conditions: A meta-analysis on 8-iso-prostaglandin F(2α). *Redox Biol* 2018;17:284-96.
70. Di Minno A, Turnu L, Porro B, et al. 8-Hydroxy-2-Deoxyguanosine Levels and Cardiovascular Disease: A Systematic Review and Meta-Analysis of the Literature. *Antioxid Redox Signal* 2016;24(10):548-55.
71. Kroese LJ, Scheffer PG. 8-hydroxy-2'-deoxyguanosine and cardiovascular disease: a systematic review. *Curr Atheroscler Rep* 2014;16(11):452.
72. Barbosa KB, Volp AC, Hermsdorff HH, et al. Relationship of oxidized low density lipoprotein with lipid profile and oxidative stress markers in healthy young adults: a translational study. *Lipids Health Dis* 2011;10:61.
73. Gao S, Zhao D, Wang M, et al. Association Between Circulating Oxidized LDL and Atherosclerotic Cardiovascular Disease: A Meta-analysis of Observational Studies. *Can J Cardiol* 2017;33(12):1624-32.
74. Il'yasova D, Scarbrough P, Spasojevic I. Urinary biomarkers of oxidative status. *Clin Chim Acta* 2012;413(19-20):1446-53.
75. Svingen GF, Schartum-Hansen H, Pedersen ER, et al. Prospective Associations of Systemic and Urinary Choline Metabolites with Incident Type 2 Diabetes. *Clin Chem* 2016;62(5):755-65.
76. Walford GA, Ma Y, Clish C, et al. Metabolite Profiles of Diabetes Incidence and Intervention Response in the Diabetes Prevention Program. *Diabetes* 2016;65(5):1424-33.
77. Topp H, Fusch G, Schöch G, et al. Noninvasive markers of oxidative DNA stress, RNA degradation and protein degradation are differentially correlated with resting metabolic rate and energy intake in children and adolescents. *Pediatr Res* 2008;64(3):246-50.
78. Sekhar RV, McKay SV, Patel SG, et al. Glutathione synthesis is diminished in patients with uncontrolled diabetes and restored by dietary supplementation with cysteine and glycine. *Diabetes Care* 2011;34(1):162-7.
79. Grizales AM, Patti ME, Lin AP, et al. Metabolic Effects of Betaine: A Randomized Clinical Trial of Betaine Supplementation in Prediabetes. *J Clin Endocrinol Metab* 2018;103(8):3038-49.

For peer review only - http://bmjopen.bmj.com/site/about/guidelines.xhtml

80. Bain MA, Faull R, Fornasini G, et al. Accumulation of trimethylamine and trimethylamine-N-oxide in end-stage renal disease patients undergoing haemodialysis. *Nephrol Dial Transplant* 2006;21(5):1300-4.
81. Chen YM, Liu Y, Zhou RF, et al. Associations of gut-flora-dependent metabolite trimethylamine-N-oxide, betaine and choline with non-alcoholic fatty liver disease in adults. *Sci Rep* 2016;6:19076.
82. Ge X, Zheng L, Zhuang R, et al. The Gut Microbial Metabolite Trimethylamine N-Oxide and Hypertension Risk: A Systematic Review and Dose-Response Meta-analysis. *Adv Nutr* 2020;11(1):66-76.
83. Heianza Y, Ma W, Manson JE, et al. Gut Microbiota Metabolites and Risk of Major Adverse Cardiovascular Disease Events and Death: A Systematic Review and Meta-Analysis of Prospective Studies. *J Am Heart Assoc* 2017;6(7)
84. Schiattarella GG, Sannino A, Toscano E, et al. Gut microbe-generated metabolite trimethylamine-N-oxide as cardiovascular risk biomarker: a systematic review and dose-response meta-analysis. *Eur Heart J* 2017;38(39):2948-56.
85. Yang S, Li X, Yang F, et al. Gut Microbiota-Dependent Marker TMAO in Promoting Cardiovascular Disease: Inflammation Mechanism, Clinical Prognostic, and Potential as a Therapeutic Target. *Front Pharmacol* 2019;10:1360.
86. Yao ME, Liao PD, Zhao XJ, et al. Trimethylamine-N-oxide has prognostic value in coronary heart disease: a meta-analysis and dose-response analysis. *BMC Cardiovasc Disord* 2020;20(1):7.
87. Aragonès G, González-García S, Aguilar C, et al. Gut Microbiota-Derived Mediators as Potential Markers in Nonalcoholic Fatty Liver Disease. *Biomed Res Int* 2019;2019:8507583.
88. Bouatra S, Aziat F, Mandal R, et al. The human urine metabolome. *PLoS One* 2013;8(9):e73076.
89. Palau-Rodriguez M, Tulipani S, Isabel Queipo-Ortuño M, et al. Metabolomic insights into the intricate gut microbial-host interaction in the development of obesity and type 2 diabetes. *Front Microbiol* 2015;6:1151.
90. Psychogios N, Hau DD, Peng J, et al. The human serum metabolome. *PLoS One* 2011;6(2):e16957.
91. Serena C, Ceperuelo-Mallafré V, Keiran N, et al. Elevated circulating levels of succinate in human obesity are linked to specific gut microbiota. *Isme j* 2018;12(7):1642-57.
92. Vojinovic D, Radjabzadeh D, Kurilshikov A, et al. Relationship between gut microbiota and circulating metabolites in population-based cohorts. *Nat Commun* 2019;10(1):5813.
93. Awoyemi A, Trøseid M, Arnesen H, et al. Markers of metabolic endotoxemia as related to metabolic syndrome in an elderly male population at high cardiovascular risk: a cross-sectional study. *Diabetol Metab Syndr* 2018;10:59.
94. Barengolts E, Green SJ, Chlipala GE, et al. Predictors of Obesity among Gut Microbiota Biomarkers in African American Men with and without Diabetes. *Microorganisms* 2019;7(9).
95. Citronberg JS, Curtis KR, White E, et al. Association of gut microbial communities with plasma lipopolysaccharide-binding protein (LBP) in premenopausal women. *Isme j* 2018;12(7):1631-41.
96. Liu X, Lu L, Yao P, et al. Lipopolysaccharide binding protein, obesity status and incidence of metabolic syndrome: a prospective study among middle-aged and older Chinese. *Diabetologia* 2014;57(9):1834-41.

For peer review only

For peer review only - http://bmjopen.bmj.com/site/about/guidelines.xhtml

SUPPLEMENTARY ONLINE MATERIAL 2

Table 1: Target daily amounts of macronutrients and functional ingredients in meals provided to the personalised plan and control groups

		Personalised plan cluster				
**Meal: Breakfast + dinner**	**Control**	**Carbohydrate**	**Microbiota**	**Lipid**	**Inflammation**	**Oxidative stress**
Energy, kcal	1200 - 1500	1200 - 1500	1200 - 1500	1200 - 1500	1200 - 1500	1200 - 1500
Protein, %	10 - 14	10 - 14	10 - 14	10 - 14	10 - 14	10 - 14
Carbohydrate, %	45 - 55	45 - 55	45 - 55	45 - 55	45 - 55	45 - 55
Dietary fibre, g	22 - 28	42 – 52*	47 – 52*	22 - 28	22 - 28	22 - 28
Added sugars, g	0	0	0	0	0	0
Fat, %	30 - 40	30 - 40	30 - 40	30 - 40	30 - 40	30 - 40
**Functional ingredient**						
Fructooligosaccharides and/or Inulin		20 g	20 g			
Sunflower oil				20 g		
Turmeric powder					6-8 g	
Oleic acid enriched sunflower oil						20 g

Values are presented in ranges (minimum-maximum) for both genders.
**Including the functional ingredient*

For peer review only - http://bmjopen.bmj.com/site/about/guidelines.xhtml

Table 2: Example of 3-day menus provided to the personalised plan and control groups during the 10-week intervention

				**Substituted or added ingredient in each cluster of the personalised plan group**				
**Day**	**Breakfast meal**	**Ingredient**	**Control (female/male)**	**Carbohydrate**	**Microbiota**	**Lipid**	**Inflammation**	**Oxidative stress**
**1**	• You Go Ghurt with granola<br>• Kale salad with orange and roasted walnuts<br>• Cucumber and celery with pumpkin seeds juice	Yogurt, You Go Ghurt	160/210 g					
		Roasted walnuts	8/10 g			Not roasted	Not roasted	Not roasted
		Granola	20/25 g	+ 5 g FOS	+ 5 g FOS			
		Orange	1 piece/medium					
		Kale	40 g				100 g coleslaw	150 g red bell pepper
		Cucumber and celery with pumpkin seeds juice	250 mL				+3 g turmeric	
**2**	• Quinoa porridge with baked strawberry compote<br>• Blood orange with pine nuts and chervil<br>• Yogurt smoothie with Mint	Quinoa porridge	40/60 g	35/55 g + 10 g inulin	35/55 g + 10 g inulin	35/55 g + 5 g seed mixture	35/55 g + 5 g seed mixture	
		Oat milk with hibiscus	130/180 mL			+ 5 g sunflower oil		+ 5 g Oleic acid enriched sunflower oil
		Strawberry compote	40 g	30 g + 10 g FOS	30 g + 10 g FOS	+5 g sunflower oil		+5 g oleic oil
		Roasted pine nuts	5 g			Not roasted	Not roasted	Not roasted
		Chervil	5 g					
		Blood orange	1 piece/medium					
		Yogurt smoothie with Mint	250 mL					
**3**	• Pearl barley salad with peanuts vinaigrette topped with roasted peanuts + 1 pear<br>• Yogurt with baked blueberries<br>• Raspberry/chili/and violet cabbage juice	Roasted peanuts	6 g			Not roasted	8 g dark chocolate 70%	8 g dark chocolate 70%
		Mint	5 g					
		Lime	20 g					
		Yogurt/You Go Ghurt	70 g					
		Baked blueberries	10 g					
		Peanut vinaigrette	18/25 g			9/13 g sunflower oil	+ 3 g turmeric	9/13 g oleic oil
		Boiled pearl barley	80/110 g					
		Pear	1 piece/medium	100 g kale	100 g kale	100 g kale		
		Raspberry/chili and violet cabbage juice	250 mL					

Continued.

For peer review only - http://bmjopen.bmj.com/site/about/guidelines.xhtml

Concluded.

				Personalised plan substituted ingredient in each cluster*				
**Day**	**Dinner meal**	**Ingredients**	**Control (female/male)**	**Carbohydrate**	**Microbiota**	**Lipid**	**Inflammation**	**Oxidative stress**
**1**	Vegetarian massaman curry with cilantro/quinoa and baked tomatoes	Massaman curry	230/325 g	220/315 g with 118 g sweet potato + 10 g inulin	220/315 g + 10 g inulin		With 122 g sweet potatoes + 5 g turmeric	Extra baked carrots - 70 g
		Rapeseed oil	1 teaspoon					
		Sesame oil dressing	20 g			6.7 g sunflower oil		6.7 Oleic oil
		Sesame seeds	4 g					
		Baby spinach	5 g					
		Cilantro	5 g					
		Cooked quinoa	110/150 g			105/145 g + 5 g seed mixture	+5 g seed mixture	
		Tomato	90 g					
		Bell pepper/red	1 piece/medium	+30 g kale	+30 g kale	+20 g kale		
**2**	Baked eggplant with green hummus/bulgur salad and Turkish flatbread	Baked eggplant	65/75 g					
		Sunflower oil	1 teaspoon					
		Flatbread with nigella sativa	120/150 g			+3 g hemp seeds	+4 g turmeric	+3 g hemp seeds
		Green hummus	60/80 g	55/75 g + 5 g inulin	55/75 g + 5 g inulin	6/8 g sunflower oil		6/8 g oleic oil
		Mint cream	40 g					
		Lemon dressing	20 g	20 g lemon dressing with 5 g FOS (replaces sirup)	20 g lemon dressing with 5 g FOS (replaces sirup)	10 g sunflower oil (replaces the canola/olive oil in standard recipe)		10 g oleic oil (replaces the canola/olive oil in standard recipe)
		Parsley	5 g					
		Bulgur	30/45 g					
		Tomato	100 g					
		Courgette	150 g	150 g red cabbage	150 g red cabbage	150 g red cabbage		120 g carrot
**3**	Trofie pasta with cauliflower sauce and fried pak-choi + green bell pepper	Cauliflower sauce	140/190 g	135/185 g + 10 g FOS	135/185 g + 10 g FOS			
		Extra virgin olive oil	2.5 g					
		Mint	5 g					
		Marinated split peas	60/80 g				4 g turmeric + 4 g hemp seed	
		Lime dressing	30 g					
		Pak-choi	90 g	100 g broccoli	100 g broccoli	100 g broccoli		100 g Chinese cabbage
		Bell pepper/green	½ piece (100 g)	75 g kale	75 g kale	75 g kale		150 g red bell pepper
		Pasta/Trofie	120/150 g	Whole grain linguine	Whole grain linguine	Whole grain linguine	Whole grain linguine	Whole grain linguine

**Added or substituted ingredients from the control diet.*
Values are presented as 'female/male' unless equal for both genders.
Abbreviations: FOS, fructooligosaccharides.

PREVENTOMICS, OSM2, page 3

For peer review only - http://bmjopen.bmj.com/site/about/guidelines.xhtml

Table 3: Nutritional information of the 3-days menus

**Day 1, Breakfast**	**Control**	**Carbohydrate**	**Microbiota**	**Lipid**	**Inflammation**	**Oxidative stress**
Energy, kcal	507/601	505/596	515/609	507/601	510/604	529/623
Protein, g (%)	14 (11)/16 (10)	13 (10)/16 (10)	14 (10)/16 (10)	14 (11)/16 (10)	13 (10)/15 (10)	14 (10)/16 (10)
Carbohydrate, g (%)	55 (43)/62 (41)	54 (42)/61 (40)	56 (43)/63 (41)	55 (43)/62 (41)	56 (44)/63 (42)	60 (45)/67 (43)
Dietary fibre, g	13/14	17/18	17/18	13/14	13/14	13/14
Sugars, g	29/31	28/29	29/31	29/31	29/30	35/35
Added sugars, g	0	0	0	0	0	0
Fat, g (%)	24 (41)/30 (44)	24 (41)/30 (44)	24 (41)/30 (43)	24 (41)/30 (44)	24 (41)/31 (44)	24 (41)/31 (44)
Saturated fat, g	4/5	4/5	4/5	4/5	4/5	4/5
Monounsaturated fat, g	3	3/4	3	3	3	3
Polyunsaturated fat, g	6/7	6/7	6/7	6/7	6/7	6/7
Salt, g	0.5	0.5	0.5	0.5	0.5	0.5
**Day 2, Breakfast**						
Energy, kcal	501/608	512/618	512/615	505/612	507/614	499/606
Protein, g (%)	12 (10)/15 (10)	12 (9)/15 (9)	12 (9)/15 (10)	12 (10)/15 (10)	13 (10)/16 (10)	12 (10)/15 (10)
Carbohydrate, g (%)	65 (53)/82 (55)	63 (50)/80 (52)	63 (50)/80 (52)	55 (44)/72 (48)	63 (50)/80 (53)	57 (46)/75 (49)
Dietary fibre, g	10/12	27/29	27/29	11/13	11/13	10/12
Sugars, g	22/24	24/33	22/24	20/23	22/24	21/23
Added sugars, g	0	0	0	0	0	0
Fat, g (%)	19 (33)/21 (31)	18 (31)/21 (29)	18 (31)/21 (29)	24 (42)/27 (38)	20 (35)/23 (33)	23 (41)/25 (37)
Saturated fat, g	3	3	3	4	3/4	3/4
Monounsaturated fat, g	2	2	1/2	3	2	3
Polyunsaturated fat, g	3	3	3	7/8	4/5	6/7
Salt, g	1	1	1	1	1	1
**Day 3, Breakfast**						
Energy, kcal	508/608	510/610	510/610	510/610	528/628	518/625
Protein, g (%)	9 (7)/10 (7)	13 (10)/15 (10)	13 (10)/15 (10)	13 (10)/15 (10)	8 (6)/10 (6)	8 (6)/10 (6)
Carbohydrate, g (%)	68 (53)/78 (52)	60 (48)/71 (47)	60 (48)/71 (47)	60 (48)/71 (47)	71 (54)/82 (52)	69 (53)/82 (54)
Dietary fibre, g	8.2/8.8	11/12	11/12	11/12	9.3/9.8	8.6/9.6
Sugars, g	29	21	21	21	31	31
Added sugars, g	0	0	0	0	0	0
Fat, g (%)	21 (37)/27 (38)	22 (38)/28 (39)	22 (38)/28 (39)	22 (38)/28 (39)	22 (37)/28 (39)	22 (37)/27 (38)

Continued.

For peer review only - http://bmjopen.bmj.com/site/about/guidelines.xhtml

Day 3, Breakfast	Control	Carbohydrate	Microbiota	Lipid	Inflammation	Oxidative stress
Saturated fat, g	3/4	3/4	3/4	3/4	5/6	5/6
Monounsaturated fat, g	5/6	5/6	5/6	5/6	4/6	4/6
Polyunsaturated fat, g	8/11	8/11	8/11	8/11	7/10	7/10
Salt, g	1	1	1	1	1	0.5
**Day 1, Dinner**						
Energy, kcal	697/881	712/890	724/907	725/908	729/907	697/881
Protein, g (%)	19 (11)/25 (11)	19 (11)/25 (11)	20 (11)/26 (11)	21 (11)/26 (12)	20 (11)/25 (11)	19 (11)/25 (11)
Carbohydrate, g	86 (50)/114 (52)	84 (48)/110 (50)	87 (48)/114 (50)	86 (48)/114 (50)	86 (48)/112 (50)	86 (50)/114 (52)
Dietary fibre, g	12/14	23/26	22/25	14/16	15/18	12/14
Sugars, g	24/28	27/33	25/29	24/28	27/32	24/28
Added sugars, g	0	0	0	0	0	0
Fat, g (%)	29 (36)/34 (34)	28 (35)/33 (33)	29 (35)/34 (33)	31 (37)/36 (35)	31 (37)/36 (35)	29 (36)/34 (34)
Saturated fat, g	7/9	7/9	7/9	7/9	8/10	7/9
Monounsaturated fat, g	7	7	7	7/8	7/8	7
Polyunsaturated fat, g	11/13	11/13	11/13	12/14	12/14	11/13
Salt, g	2/3	2/3	2/3	2/3	2/3	2/3
**Day 2, Dinner**						
Energy, kcal	704/871	731/897	731/897	742/912	718/884	742/901
Protein, g (%)	21 (12)/26 (12)	21 (11)/26 (12)	21 (11)/26 (12)	22 (12)/28 (12)	21 (12)/27 (12)	21 (11)/26 (12)
Carbohydrate, g	90 (54)/116 (51)	95 (52)/121 (54)	95 (52)/121 (54)	96 (52)/122 (53)	92 (51)/118 (54)	96 (52)/122 (54)
Dietary fibre, g	14/17	25/28	25/28	15/18	15/18	16/18
Sugars, g	5/6	11/12	11/12	11	5/6	12/13
Added sugars, g	0	0	0	0	0	0
Fat, g (%)	27 (33)/31 (30)	25 (30)/29 (28)	25 (30)/29 (28)	28 (32)/29 (31)	27 (33)/31 (30)	28 (33)/32 (30)
Saturated fat, g	3/4	3/4	3/4	3/4	4	4
Monounsaturated fat, g	12/15	12/14	12/14	13/15	12/15	13/15
Polyunsaturated fat, g	8/9	7/8	7/8	9/10	8/9	9/10
Salt, g	3/4	3/4	3/4	3/4	3/4	3/4
**Day 3, Dinner**						
Energy, kcal	699/877	731/909	731/909	722/900	732/911	723/901
Protein, g (%)	23 (13)/28 (13)	25 (14)/31 (14)	25 (14)/31 (14)	25 (14)/31 (14)	24 (13)/30 (13)	24 (13)/30 (13)
Carbohydrate, g (%)	105 (60)/131 (60)	106 (58)/131 (58)	106 (58)/131 (58)	106 (58)/131 (59)	107 (59)/133 (58)	109 (60)/135 (61)

Continued.

For peer review only - http://bmjopen.bmj.com/site/about/guidelines.xhtml

Concluded.

Day 3, Dinner	Control	Carbohydrate	Microbiota	Lipid	Inflammation	Oxidative stress
Dietary fibre, g	11/13	22/25	22/25	14/16	12/14	11/14
Sugars, g	20/23	19/22	19/22	20/23	20/23	24/27
Added sugars, g	0	0	0	0	0	0
Fat, g (%)	19 (24)/24 (24)	19 (22)/25 (23)	19 (22)/25 (23)	19 (23)/25 (24)	21 (25)/26 (25)	19 (23)/25 (24)
Saturated fat, g	3/4	3/4	3/4	3/4	3/4	3/4
Monounsaturated fat, g	7/10	7/9	7/9	7/10	8/10	7/9
Polyunsaturated fat, g	6/8	6/8	6/8	7/9	7/9	6/8
Salt, g	2/3	2/3	2/3	2/3	2/3	2/3

Values are presented as 'female/male' unless equal for both genders.

For peer review only

For peer review only - http://bmjopen.bmj.com/site/about/guidelines.xhtml

SUPPLEMENTARY ONLINE MATERIAL 3: informed consent version 2.0, 18 September 2020

Title of research project: **PREVENTOMICS: Empowering consumers to PREVENT diet-related diseases through OMICS-based personalized nutrition**

Consent from the study participant:

I have obtained written and oral information about the research project and I am informed about the aim, methods, benefits and risks of participating in the study.

I know that it is completely voluntary to participate, and I have the right to withdraw the informed consent at any time and with no loss of benefits to which I am otherwise entitled.

I consent to participate in the research project and that my biological material will be stored in a research biobank. I have received a copy of this informed consent form as well as a copy of the written information.

Study participant name: ____

Date: _______________ Signature: ____

In case new information that has substantial influence on your health emerges from the research project, you will be informed. Would you prefer **not** to be informed about information that has substantial influence on your health, please mark it here _________ (insert X).

Do you wish to be informed about the final result of the research project and the potential consequences for you:

Yes _____ (insert X) No _____ (insert X)

Consent from the study staff that provided the oral information:

I declare, that the participant has received both written and oral information about the research project.

I declare to the best of my knowledge and belief that the participant has received sufficient information to decide to participate in the research project.

Study staff name: ____

Date: _______________ Signature: ____

National project identification: H-20029882

For peer review only

Reporting checklist for protocol of a clinical trial

SPIRIT

STANDARD PROTOCOL ITEMS: RECOMMENDATIONS FOR INTERVENTIONAL TRIALS

		Reporting Item	Page Number
**Administrative information**			
Title	#1	Descriptive title identifying the study design, population, interventions, and, if applicable, trial acronym	1
Trial registration	#2a	Trial identifier and registry name. If not yet registered, name of intended registry	2
Trial registration: data set	#2b	All items from the World Health Organization Trial Registration Data Set	N/A
Protocol version	#3	Date and version identifier	19,20
Funding	#4	Sources and types of financial, material, and other support	20
Roles and responsibilities: contributorship	#5a	Names, affiliations, and roles of protocol contributors	1,20
Roles and responsibilities: sponsor contact information	#5b	Name and contact information for the trial sponsor	20
Roles and responsibilities: sponsor and funder	#5c	Role of study sponsor and funders, if any, in study design; collection, management, analysis, and interpretation of data; writing of the report; and the decision to submit the report for publication, including whether they will have ultimate authority over any of these activities	18-20

For peer review only

For peer review only - http://bmjopen.bmj.com/site/about/guidelines.xhtml

Roles and responsibilities: committees	#5d	Composition, roles, and responsibilities of the coordinating centre, steering committee, endpoint adjudication committee, data management team, and other individuals or groups overseeing the trial, if applicable (see Item 21a for data monitoring committee)	N/A
**Introduction**			
Background and rationale	#6a	Description of research question and justification for undertaking the trial, including summary of relevant studies (published and unpublished) examining benefits and harms for each intervention	4, 5
Background and rationale: choice of comparators	#6b	Explanation for choice of comparators	4, 5
Objectives	#7	Specific objectives or hypotheses	5
Trial design	#8	Description of trial design including type of trial (eg, parallel group, crossover, factorial, single group), allocation ratio, and framework (eg, superiority, equivalence, non-inferiority, exploratory)	6, Figure 1
**Methods: Participants, interventions, and outcomes**			
Study setting	#9	Description of study settings (eg, community clinic, academic hospital) and list of countries where data will be collected. Reference to where list of study sites can be obtained	6
Eligibility criteria	#10	Inclusion and exclusion criteria for participants. If applicable, eligibility criteria for study centres and individuals who will perform the interventions (eg, surgeons, psychotherapists)	6

For peer review only

Interventions: description	#11a	Interventions for each group with sufficient detail to allow replication, including how and when they will be administered	7-13, Tables 1-5, Supplemental material 1 and 2
Interventions: modifications	#11b	Criteria for discontinuing or modifying allocated interventions for a given trial participant (eg, drug dose change in response to harms, participant request, or improving / worsening disease)	6, 18
Interventions: adherance	#11c	Strategies to improve adherence to intervention protocols, and any procedures for monitoring adherence (eg, drug tablet return; laboratory tests)	13
Interventions: concomitant care	#11d	Relevant concomitant care and interventions that are permitted or prohibited during the trial	10-13, Table 4
Outcomes	#12	Primary, secondary, and other outcomes, including the specific measurement variable (eg, systolic blood pressure), analysis metric (eg, change from baseline, final value, time to event), method of aggregation (eg, median, proportion), and time point for each outcome. Explanation of the clinical relevance of chosen efficacy and harm outcomes is strongly recommended	13-17, Table 5
Participant timeline	#13	Time schedule of enrolment, interventions (including any run-ins and washouts), assessments, and visits for participants. A schematic diagram is highly recommended (see Figure)	6, 7, Figure 2
Sample size	#14	Estimated number of participants needed to achieve study objectives and how it was determined, including clinical and statistical assumptions supporting any sample size calculations	18
Recruitment	#15	Strategies for achieving adequate participant enrolment to reach target sample size	6

For peer review only

**Methods: Assignment of interventions (for controlled trials)**			
Allocation: sequence generation	#16a	Method of generating the allocation sequence (eg, computer-generated random numbers), and list of any factors for stratification. To reduce predictability of a random sequence, details of any planned restriction (eg, blocking) should be provided in a separate document that is unavailable to those who enrol participants or assign interventions	9,10
Allocation concealment mechanism	#16b	Mechanism of implementing the allocation sequence (eg, central telephone; sequentially numbered, opaque, sealed envelopes), describing any steps to conceal the sequence until interventions are assigned	9, 10
Allocation: implementation	#16c	Who will generate the allocation sequence, who will enrol participants, and who will assign participants to interventions	9
Blinding (masking)	#17a	Who will be blinded after assignment to interventions (eg, trial participants, care providers, outcome assessors, data analysts), and how	9, 10
Blinding (masking): emergency unblinding	#17b	If blinded, circumstances under which unblinding is permissible, and procedure for revealing a participant's allocated intervention during the trial	N/A
**Methods: Data collection, management, and analysis**			
Data collection plan	#18a	Plans for assessment and collection of outcome, baseline, and other trial data, including any related processes to promote data quality (eg, duplicate measurements, training of assessors) and a description	13-17, Table 5

For peer review only

		of study instruments (eg, questionnaires, laboratory tests) along with their reliability and validity, if known. Reference to where data collection forms can be found, if not in the protocol	
Data collection plan: retention	#18b	Plans to promote participant retention and complete follow-up, including list of any outcome data to be collected for participants who discontinue or deviate from intervention protocols	13-14
Data management	#19	Plans for data entry, coding, security, and storage, including any related processes to promote data quality (eg, double data entry; range checks for data values). Reference to where details of data management procedures can be found, if not in the protocol	18-19
Statistics: outcomes	#20a	Statistical methods for analysing primary and secondary outcomes. Reference to where other details of the statistical analysis plan can be found, if not in the protocol	19
Statistics: additional analyses	#20b	Methods for any additional analyses (eg, subgroup and adjusted analyses)	19
Statistics: analysis population and missing data	#20c	Definition of analysis population relating to protocol non-adherence (eg, as randomised analysis), and any statistical methods to handle missing data (eg, multiple imputation)	19
**Methods: Monitoring**			
Data monitoring: formal committee	#21a	Composition of data monitoring committee (DMC); summary of its role and reporting structure; statement of whether it is independent from the sponsor and competing interests; and reference to where further details about its charter can be found, if not in the protocol. Alternatively, an explanation of why a DMC is not needed	18

For peer review only

Data monitoring: interim analysis	#21b	Description of any interim analyses and stopping guidelines, including who will have access to these interim results and make the final decision to terminate the trial	18
Harms	#22	Plans for collecting, assessing, reporting, and managing solicited and spontaneously reported adverse events and other unintended effects of trial interventions or trial conduct	18
Auditing	#23	Frequency and procedures for auditing trial conduct, if any, and whether the process will be independent from investigators and the sponsor	N/A
**Ethics and dissemination**			
Research ethics approval	#24	Plans for seeking research ethics committee / institutional review board (REC / IRB) approval	19
Protocol amendments	#25	Plans for communicating important protocol modifications (eg, changes to eligibility criteria, outcomes, analyses) to relevant parties (eg, investigators, REC / IRBs, trial participants, trial registries, journals, regulators)	19
Consent or assent	#26a	Who will obtain informed consent or assent from potential trial participants or authorised surrogates, and how (see Item 32)	19
Consent or assent: ancillary studies	#26b	Additional consent provisions for collection and use of participant data and biological specimens in ancillary studies, if applicable	19
Confidentiality	#27	How personal information about potential and enrolled participants will be collected, shared, and maintained in order to protect confidentiality before, during, and after the trial	18, 19

For peer review only

Declaration of interests	#28	Financial and other competing interests for principal investigators for the overall trial and each study site	20
Data access	#29	Statement of who will have access to the final trial dataset, and disclosure of contractual agreements that limit such access for investigators	19
Ancillary and post trial care	#30	Provisions, if any, for ancillary and post-trial care, and for compensation to those who suffer harm from trial participation	N/A
Dissemination policy: trial results	#31a	Plans for investigators and sponsor to communicate trial results to participants, healthcare professionals, the public, and other relevant groups (eg, via publication, reporting in results databases, or other data sharing arrangements), including any publication restrictions	19
Dissemination policy: authorship	#31b	Authorship eligibility guidelines and any intended use of professional writers	19
Dissemination policy: reproducible research	#31c	Plans, if any, for granting public access to the full protocol, participant-level dataset, and statistical code	N/A
**Appendices**			
Informed consent materials	#32	Model consent form and other related documentation given to participants and authorised surrogates	Supplemental Material 3
Biological specimens	#33	Plans for collection, laboratory evaluation, and storage of biological specimens for genetic or molecular analysis in the current trial and for future use in ancillary studies, if applicable	17, 18

The SPIRIT Explanation and Elaboration paper is distributed under the terms of the Creative Commons Attribution License CC-BY-NC. This checklist was completed on 02. March 2021 using https://www.goodreports.org/, a tool made by the EQUATOR Network in collaboration with Penelope.ai

For peer review only
